# Factors affecting the expression and stability of full-length and truncated SRSF3 proteins in human cancer cells

**DOI:** 10.1038/s41598-024-64640-1

**Published:** 2024-06-22

**Authors:** Sung-How Sue, Shu-Ting Liu, Shih-Ming Huang

**Affiliations:** 1 Department of Cardiovascular Surgery, Chung Shan Medical University Hospital, Chung Shan Medical University, Taichung City, 402 Taiwan, Republic of China; 2 Institute of Medicine, Chung Shan Medical University, Taichung City, 402 Taiwan, Republic of China; 3 Department of Biochemistry, National Defense Medical Center, Taipei City, 114 Taiwan, Republic of China

**Keywords:** Alternative splicing, Serine/arginine-rich splicing factors, Truncated protein, Premature termination codon, Palmitic acid, Biochemistry, Molecular biology

## Abstract

Alternative splicing plays a crucial role in increasing the diversity of mRNAs expressed in the genome. Serine/arginine-rich splicing factor 3 (SRSF3) is responsible for regulating the alternative splicing of its own mRNA and ensuring that its expression is balanced to maintain homeostasis. Moreover, the exon skipping of *SRSF3* leads to the production of a truncated protein instead of a frameshift mutation that generates a premature termination codon (PTC). However, the precise regulatory mechanism involved in the splicing of *SRSF3* remains unclear. In this study, we first established a platform for coexpressing full-length SRSF3 (*SRSF3-FL)* and *SRSF3-PTC* and further identified a specific antibody against the SRSF3-FL and truncated SRSF3 (SRSF3-TR) proteins. Next, we found that exogenously overexpressing *SRSF3-FL* or *SRSF3-PTC* failed to reverse the effects of digoxin, caffeine, or both in combination on this molecule and its targets. Endoplasmic reticulum-related pathways, transcription factors, and chemicals such as palmitic acid and phosphate were found to be involved in the regulation of *SRSF3* expression. The downregulation of SRSF3-FL by palmitic acid and phosphate was mediated via different regulatory mechanisms in HeLa cells. In summary, we provide new insights into the altered expression of the SRSF3-FL and SRSF3-TR proteins for the identification of the functions of SRSF3 in cells.

## Introduction

The primary processes involved in eukaryotic gene expression are transcription, splicing, and translation. Due to the presence of multiple splicing sites in pre-mRNAs, alternative splicing generates several splicing isoforms in various cell types or biological processes. This overall function of alternative splicing is essential for increasing the diversity of mRNAs expressed in the genome. Moreover, the splicing programs that regulate different genes can have major physiological consequences^1^. Specific trans-factors, such as serine/arginine-rich splicing factors (SRSFs) and heterogeneous nuclear ribonucleoproteins (hnRNPs), are responsible for alternatively splicing most human pre-mRNA transcripts. However, the role and regulatory mechanisms of alternative splicing in various biological processes are not well established^2–4^. Due to the identification of 12 classic SRSFs, the new nomenclature is now based on the root "SRSF", followed by the numbers 1–12, which was developed in 2010^5^^,^^6^. The modular structure of SRSFs includes one or two RNA recognition motifs (RRMs) at their amino terminus and an arginine/serine-rich (RS) domain that varies in length at the carboxyl terminus. Both domains have the ability to directly interact with RNA, with RRM being responsible for RNA binding specificity. Knockout studies have revealed that SRSF3 plays a crucial role in mouse development, hepatocyte differentiation, metabolic function, and tumor cell proliferation and maintenance^7–10^. Hence, SRSF3 is a potential therapeutic target for various cancers.

SRSF3 is the first known example of a second SRSF antagonizing the autoregulatory activity of a splicing factor. Researchers from the laboratories of Dr. Jumaa and Nielson demonstrated that SRSF3 modifies the splicing of its own mRNA, and this modulation is countered by SRSF1^11^. In oral squamous cell carcinoma cells, polypyrimidine tract binding protein (PTBP) 1 and PTBP2 were shown to bind to exonic splicing suppressors of *SRSF3 exon 4* to prevent its inclusion, leading to the upregulation of full-length *SRSF3* (*SRSF3-FL*). mRNAs containing a premature termination codon (PTC) are degraded via the nonsense-mediated mRNA decay (NMD) pathway under normal conditions^12^. However, serum starvation or oxidative stress can induce the inclusion of exon 4 during *SRSF3* pre-mRNA splicing. SRSF3 regulates its own expression by promoting the inclusion of exon 4 (*SRSF3-PTC*), which carries a stop codon and results in the expression of truncated SRSF3 (SRSF3-TR). The knockout of *SRSF3-PTC* led to a significant reduction in arsenite-induced JUN protein expression, the binding and promoter activity of the transcription factor JUN in the *IL-8* promoter, and, consequently, the expression of *IL-8*^12^. Four mechanisms that control *SRSF3* exon 4 splicing have been identified^13,14^. First, SRSF3 can promote the inclusion of exon 4 and establish negative feedback regulation. Second, several other splicing factors can also regulate exon 4 splicing. Third, cell proliferation and stress are associated with exon 4 splicing. Finally, chemicals/drugs that regulate exon 4 splicing, such as digoxin and caffeine, have also been reported. However, the specific regulatory mechanism of SRSF3 splicing remains unclear.

SRSF3 is responsible for regulating the alternative splicing of its own mRNA and autoregulating expression to maintain homeostasis^11^. The autoregulation of *SRSF3* was first reported, and the overexpression of SRSF3 led to a reduction in the level of *SRSF3-FL* and activated the production of *SRSF3-PTC*. Therefore, an increase in the inclusion of exon 4 in *SRSF3* mRNA splicing can lead to a reduction in the expression of the SRSF3-FL protein and potentially induce the expression of SRSF3-TR proteins. Our previous research revealed that caffeine, digoxin, theophylline, and amiodarone can induce the downregulation of *SRSF3-FL* expression and the upregulation of *SRSF3-PTC* expression^15–18^. Palmitic acid–induced oxidative stress causes the conjugation of the NEDD8 protein to SRSF3 for proteasome-mediated degradation^19^. Sodium arsenite increased *SRSF3-PTC* mRNA levels without affecting *SRSF3-FL* mRNA levels in a human colon cancer cell line^12^. A recent study demonstrated that SRSF3 regulates cellular senescence via alternative splicing of p53 to switch the alpha isoform to the beta isoform^20^. Many studies have shown that metformin, cupric sulfate, ascorbate, phosphate ion (Pi), and all-trans retinoic acid (atRA) modulate the expression of p53 proteins in cancer cells^21–23^. In this study, we aimed to investigate the mRNA and protein expression levels of *SRSF3-FL* and *SRSF3-PTC* under normal conditions or under stress in human cervical cancer cells. We utilized overexpression systems, such as *SRSF3-FL* and *SRSF3-PTC* expression vectors and the *SRSF3* promoter reporter system, to elucidate the complexities of endogenous *SRSF3* gene regulation and protein stability. We applied different ratios of *SRSF3-FL* and *SRSF3-PTC* expression plasmids to verify the potential of the SRSF3 antibody because of the large difference in affinity between the SRSF3-FL and SRSF3-TR proteins. Additionally, we examined the regulatory mechanisms of these *SRSF3* mRNAs, including transcription factors and drugs. Our findings provide new insights into the characteristics of full-length and truncated SRSF3 proteins in cancer cells.

## Results

### Establishing an analytic platform for the expression of full-length and truncated SRSF3 proteins in HeLa cells

**Figure 1:**
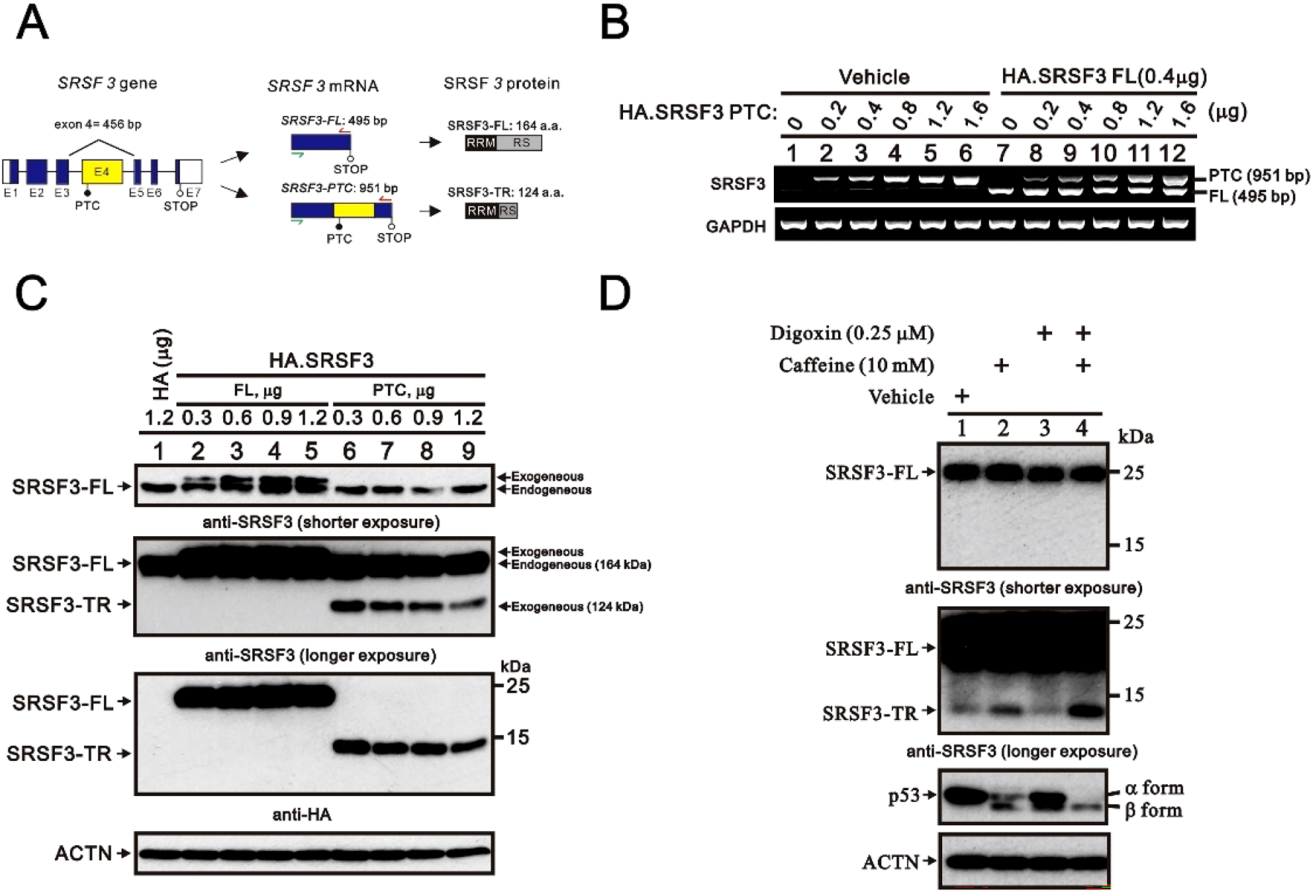
The SRSF3-FL and SRSF3-TR proteins were verified via exogenous transfection of the *SRSF3-FL* and *SRSF3-PTC* mRNAs in HeLa cells. (**A**) Schematic diagram of the *SRSF3* gene and structures of SRSF3 proteins. The functional SRSF3 protein (SRSF3-FL) is translated from the *SRSF3-FL* mRNA isoform. The exon 4-containing mRNA isoform (*SRSF3-PTC*) is translated into a truncated protein, SRSF3-TR. E: exon; RRM, RNA recognition motif; RS, arginine/serine-rich domain. The green and red arrows represent the 5′ and 3′ primers for *SRSF3-FL* and SRSF3*-PTC*, according to RT‒PCR analysis. (**B**) HeLa cells were transiently transfected with 1.6 µg of pSG5.HA vector and the indicated amount of *pSG5.HA.SRSF3–PTC* or 0.4 µg of *pSG5.HA.SRSF3–FL*. These cells were collected and subjected to RT‒PCR for the detection of *SRSF3-FL, SRSF3-PTC* and *GAPDH* (a loading control). HeLa cells were (**C**) transiently transfected with 1 µg of the pSG5.HA vector and the indicated amount of *pSG5.HA.SRSF3–FL* and *–PTC*; (**D**) treatment with the indicated amount of caffeine or/and digoxin. These cells were collected and subjected to immunoblot analysis for the detection of SRSF3 (purchased from the Sigma), HA, p53, and ACTN (a loading control) (supplementary file 1).

We first constructed two HA-tagged *SRSF3* expression vectors, one for full-length *SRSF3* mRNA *(SRSF3-FL*; 495 bp) and one with a PTC of *SRSF3* mRNA (*SRSF3–PTC*; 951 bp), which encoded the full-length (164*.SRSF3–PTC*, alone or in combination, with 0.4 μg HA.*SRSF3-FL* for 24 h in HeLa cells. Our RT‒PCR analysis revealed that the 951 bp *SRSF3–PTC* mRNA bands showed a dose-dependent pattern or were coexpressed with the constant expression of the 495 bp *SRSF3-FL* mRNA bands (Fig. 1B). The exogenous *SRSF3* mRNA expression was greater than the endogenous *SRSF3* mRNA expression in HeLa cells.

Based on our two current SRSF3 constructs, it is important to find a suitable commercial antibody that can identify both proteins in the same gel. One SRSF3 antibody, purchased from Sigma, was screened, and we verified its ability to recognize both SRSF3 proteins (SRSF3-FL and SRSF3-TR) on the same blot using the HA-tag strategy in HeLa cells (Fig. 1C). We observed endogenous and exogenous SRSF3-FL and exogenous SRSF3-TR proteins via one specific SRSF3 antibody and further verified these findings using a specific HA antibody. We further identified the inductive effects of caffeine, digoxin, and both in combination on endogenous SRSF3-TR proteins in HeLa cells using a Sigma commercial antibody (Fig. 1D). With Western blotting and antibodies, we identified far fewer endogenous truncated SRSF3 fragments than full-length SRSF3 fragments, accompanied by the switch of the p53α form to the β form. Caffeine had a better effect than digoxin, and the combination of caffeine and digoxin had a better effect than caffeine alone on the induction of the truncated SFSF3 protein and the switch of p53α to the β form.

### Characterization of the effects of digoxin and/or caffeine on full-length and truncated SRSF3 proteins in HeLa cells

**Figure 2:**
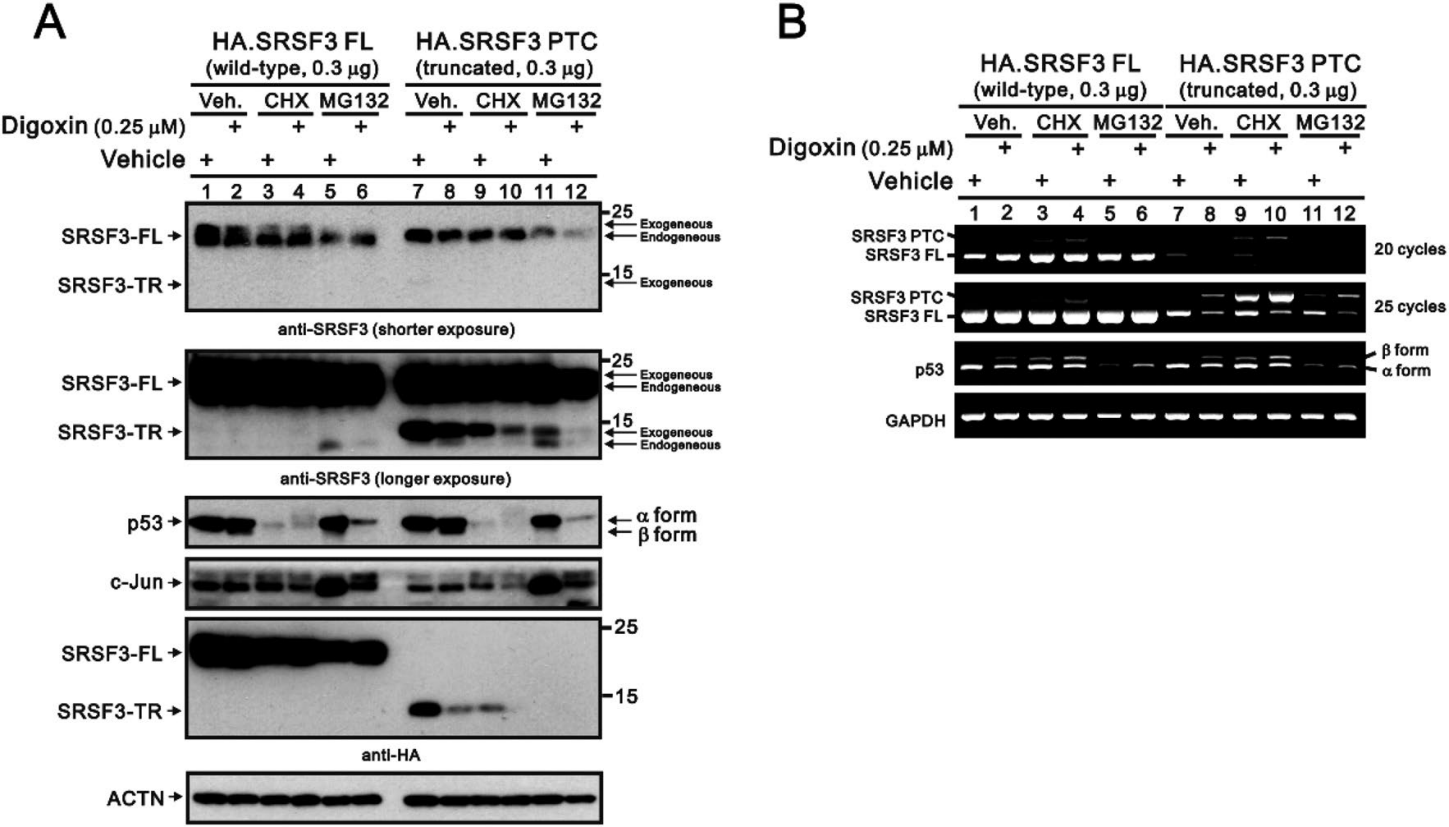
The effects of digoxin on the protein and mRNA stability of SRSF3-FL and SRSF3-TR in HeLa cells. HeLa cells were transiently transfected with 0.3 µg of *pSG5.HA. SRSF3–FL* and *–PTC*. After 16 h, the transfected cells were treated with 0.25 µM digoxin for 16 h and pretreated with the vehicle, either 50 µg/ml CHX or 10 µM MG132, for 2 h. (**A**) These cells were collected and subjected to immunoblot analysis for the detection of SRSF3, p53, c-Jun, and ACTN (a loading control). (**B**) mRNA was extracted and analyzed via RT‒PCR (with *GAPDH* as a loading control). The results are representative of two independent experiments.

In addition to the splicing of the *SRSF3* genes, the downregulation of SRSF3-FL and the increase in SRSF3-TR proteins that occurs at the translation level might be addressed via the addition of a de novo protein synthesis inhibitor, cycloheximide (CHX), and the proteasome-dependent degradation inhibitor MG132, respectively. CHX did not show different effects on the exogenous and endogenous SRSF3-FL proteins (Fig. 2A, columns 3 and 4 or columns 9 and 10), whereas digoxin treatment reduced the stability of exogenous SRSF3-TR proteins (Fig. 2A, columns 9 and 10). Downregulation of the endogenous SRSF3-FL protein and an increase in the endogenous SRSF3-TR protein were observed when HeLa cells were pretreated with MG132, revealing the effects of MG132, caffeine and digoxin (Fig. 2A, columns 5 and 6 and columns 11 and 12). Our current data suggest that SRSF3 might be the primary target of the NMD pathway because of the increased stability of endogenous SRSF3-TR proteins in the presence of MG132 (Fig. 2A). We monitored the p53 and c-Jun proteins as positive controls for CHX and MG132 treatment, respectively, because p53 has a short half-life of approximately 5 min, and the degradation of c-Jun is primarily mediated through the ubiquitin-dependent proteasome pathway. According to the mRNA analysis, CHX treatment induced the expression of endogenous and exogenous *SRSF3* in *PTCs* (Fig. 2B). The *β* form of *p53* mRNA increased with CHX treatment, but MG132 reduced the *α* form of *p53* mRNA, and digoxin induced the *β* form of *p53* mRNA.

**Figure 3:**
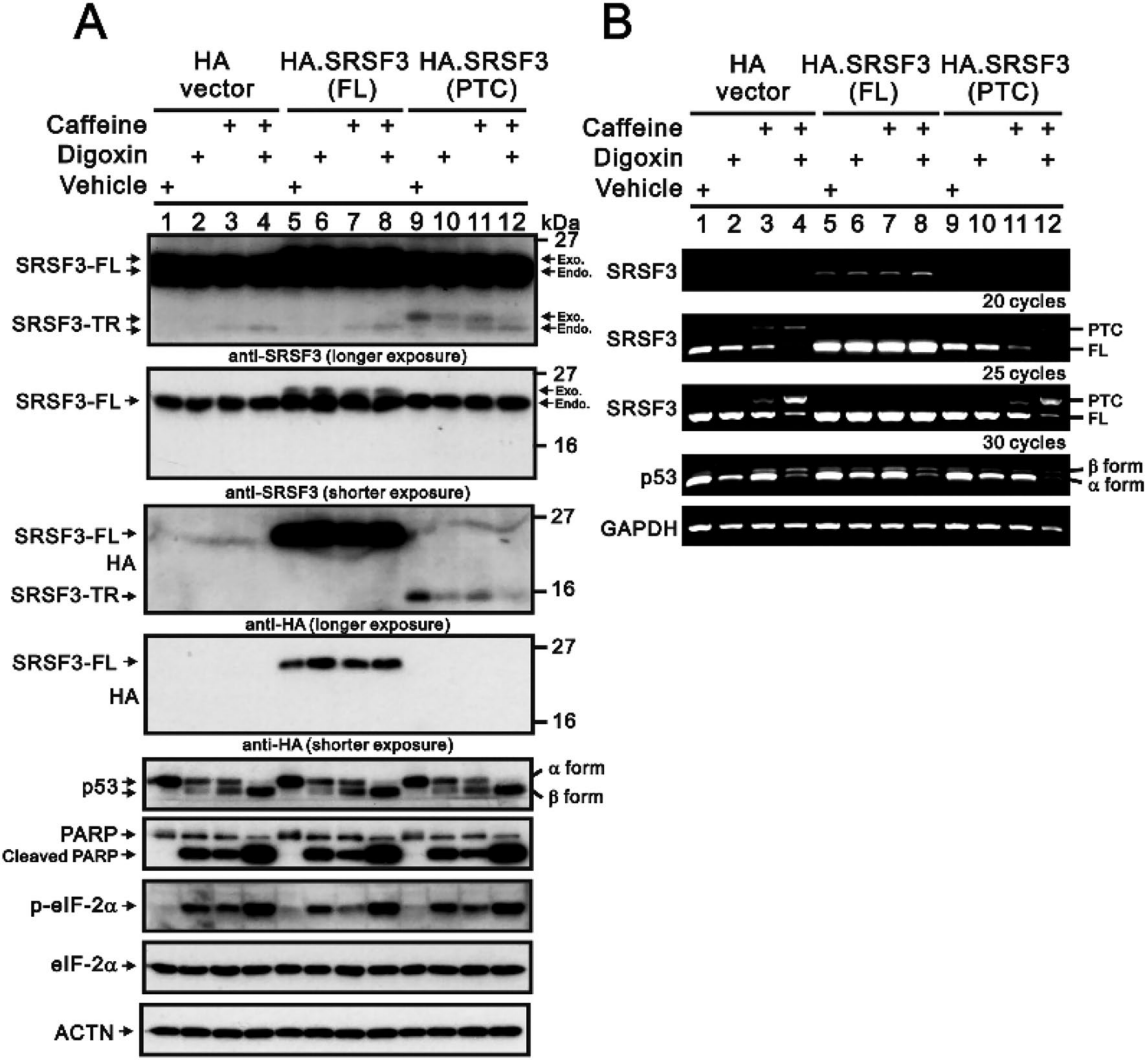
The effects of exogenously transfected *SRSF3-FL* and *SRSF3-PTC* mRNAs on endogenous SRSF3-FL and SRSF3-TR proteins were examined in HeLa cells. HeLa cells were transiently transfected with 0.5 µg of the pSG5.HA vector, *pSG5.HA.SRSF3–FL*, or *pSG5.HA.SRSF3–PTC*. After 16 h, the transfected cells were treated with 0.25 µM digoxin, 10 mM caffeine, or both in combination for 24 h. (**A**) These cells were collected and subjected to immunoblot analysis for the detection of the indicated proteins and ACTN (a loading control). Exo: exogenous; Endo: endogenous. (**B**) These cells were collected and subjected to RT‒PCR for the detection of the indicated mRNAs and *GAPDH* (a loading control).

Figure 1C shows the inductive effects of caffeine, digoxin, and both in combination on endogenous SRSF3-TR proteins in HeLa cells. Here, we examined the effects of caffeine, digoxin, and both in combination on the effects of exogenously transfected *SRSF3-FL* and *SRSF3-PTC* in HeLa cells (Fig. 3). In the Western blot analysis, we first observed similar effects of caffeine, digoxin, and both in combination on the endogenous SRSF-FL and SRSF3-TR proteins using a specific antibody against the SRSF3 protein in HA-transfected HeLa cells (Fig. 3A). Using an HA-tag antibody, we found that the expression of exogenously transfected SRSF3-FL proteins was increased and that the expression of exogenously transfected SRSF3-TR proteins was decreased by the combination of caffeine, digoxin, and both. Other assessed proteins, such as p53 α/β, cleaved PARP, and the p-eIF2α/eIF2α ratio, were differentially affected by caffeine, digoxin, and both in combination, but no further effect was found regarding the exogenous expression of the SRSF3-FL and SRSF3-TR proteins in HeLa cells. According to the RT‒PCR analysis, exogenously transfected *SRSF3-FL* inhibited the effects of the caffeine and digoxin combinations on endogenous *SRSF3-PTC* but not on the switch from *p53α* mRNA to *p53β* mRNA in HeLa cells (Fig. 3B). Exogenously transfected *SRSF3-PTC* repressed the effects of the caffeine and digoxin combinations on endogenous *SRSF3-PTC* and *p53α* and* β* mRNAs in HeLa cells.

### Screening the effects of target proteins and drugs on full-length and truncated SRSF3 proteins in HeLa cells

**Figure 4:**
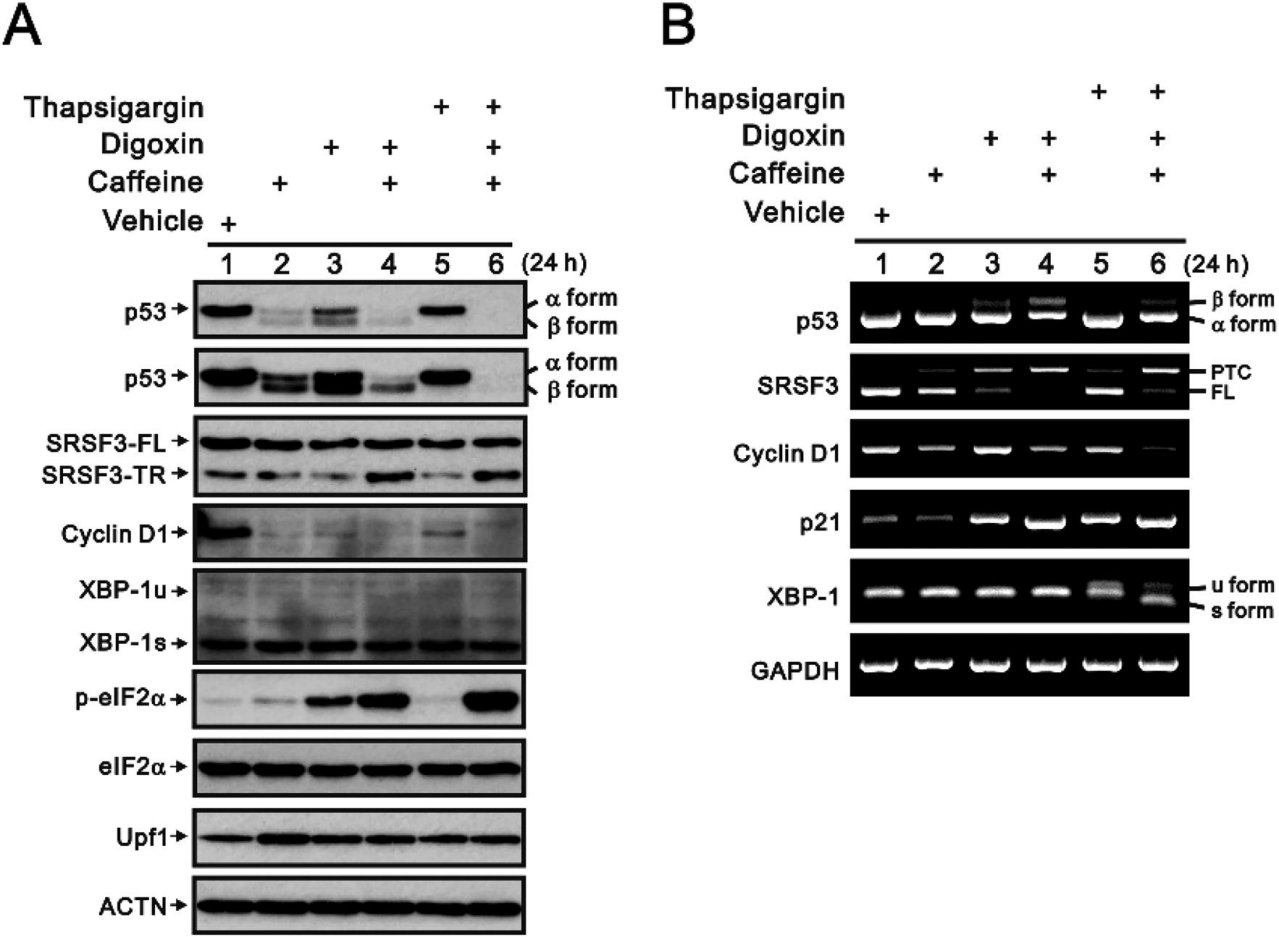
The effects of digoxin on the protein and mRNA stability of SRSF3-FL and SRSF3-TR in HeLa cells. HeLa cells were treated with 0.25 µM digoxin, 10 mM caffeine, 100 nM thapsigargin, or combinations of two or all three components for 24 h. (**A**) These cells were collected and subjected to immunoblot analysis for the detection of the indicated proteins and ACTN (a loading control). (**B**) mRNA was extracted and analyzed via RT‒PCR (with *GAPDH* as a loading control).

Dr. You's laboratory demonstrated that cardiac glycosides can exert inhibitory effects on the NMD pathway by binding to and inhibiting the sodium–potassium ATPase on the plasma membrane. This, in turn, leads to an increase in intracellular calcium levels. Their study utilized a reporter system that screened hundreds of clinical drugs [22]. The list of positive drugs, which included caffeine (positive control in their screening system) and digoxin (the top drug in their screening list), suggested that the increase in the intracellular cyclic AMP (cAMP) level may play an important role in the inhibitory effects on the NMD pathway. In this study, we used thapsigargin, a calcium and endoplasmic reticulum (ER) stress inducer, to compare the effects of digoxin and caffeine on HeLa cells. We first examined whether digoxin and caffeine, which are well-known inhibitors of NMD activity, can induce ER stress. Our data confirmed that digoxin, caffeine, or both may induce ER stress via the PERK pathway to phosphorylate e-IF2α in HeLa cells (Fig. 4A); we also found that thapsigargin activated the IRE1 pathway to splice *XBP-1* (Fig. 4B). In contrast, we observed upregulation of the UPF1 protein, the key protein of the NMD system (Fig. 4A).

**Figure 5:**
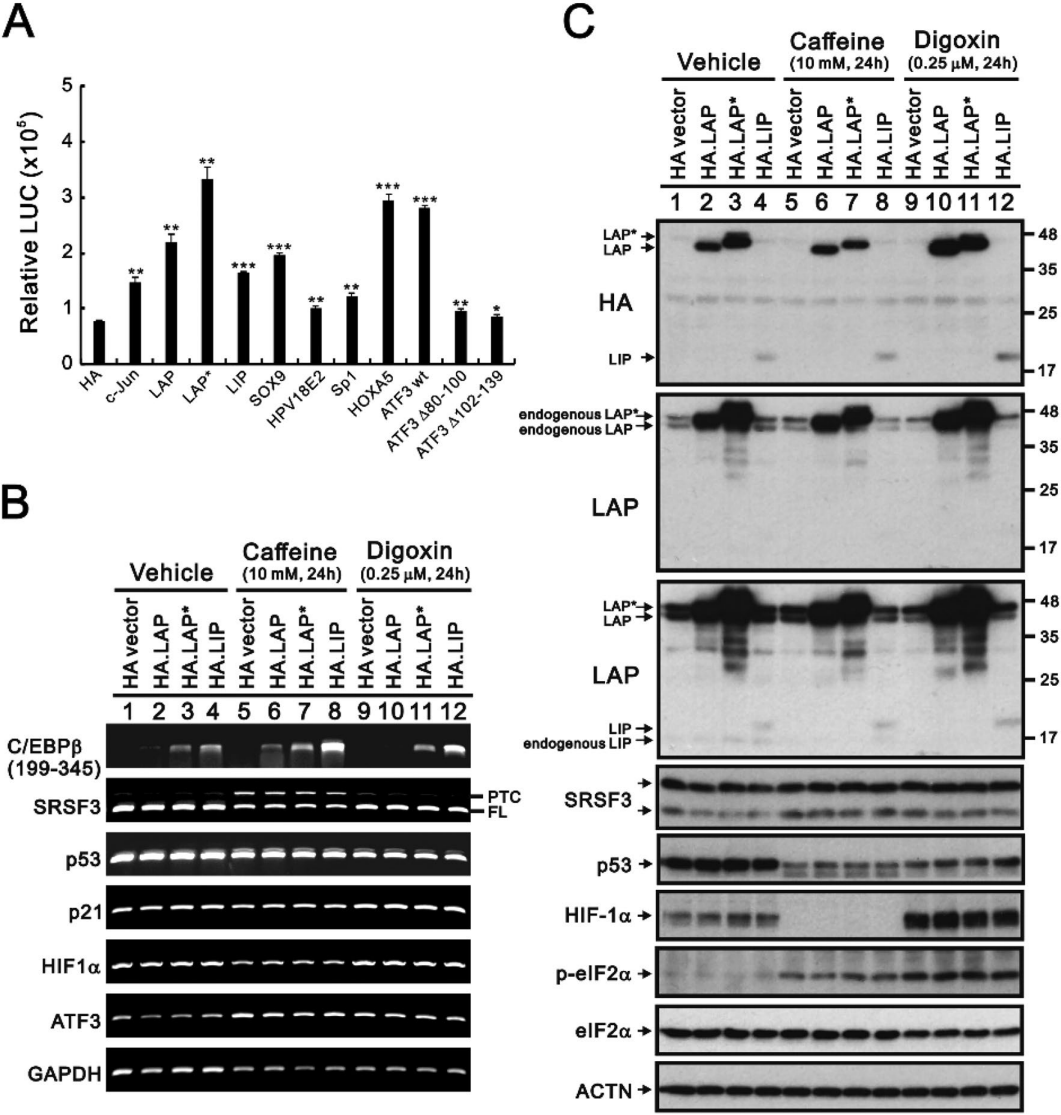
Effects of LAP*, LAP, and LIP on the expression of *SRSF3* in HeLa cells. (**A**) HeLa cells were transfected with 0.25 μg of *SRSF3–LUC* and 0.25 μg of the indicated genes. Luciferase activities in the transfected cell extracts were then determined (RLU indicates relative light units). These data are the average of three independent experiments (mean ± S.D.; *n* = 3). Student’s t tests were performed, and the results were compared with those obtained with the HA vector alone. * *p* < 0.05, ** *p* < 0.01, and *** p < 0.001. HeLa cells were transiently transfected with 0.5 μg of *pSG5.HA.LAP*, LAP*, and *LIP*. HeLa cells were treated with 10 mM caffeine or 0.25 μM digoxin for 24 h. (**B**) mRNA was extracted and analyzed via RT‒PCR (with *GAPDH* as a loading control). (**C**) These cells were collected and subjected to immunoblot analysis for the indicated proteins and ACTN (a loading control). The results are representative of two independent experiments.

Based on our published and preliminary results, we constructed an *SRSF3* promoter to further elucidate the kinds of responsive elements and transcription factors involved in *SRSF3* gene expression. Here, using transient transfection analysis, we tested our constructed *SRSF3* (− 1650/ + 171) promoter reporter construct with many transcription factors. Our data suggested that many transcription factors might be involved in the regulation of *SRSF3* gene expression. Our findings showed that c-Jun, CCAAT-enhancer-binding protein beta (C/EBP β), SOX9, Sp1, HOXA5, and ATF3 play a positive role in SRSF3 promoter activity (Fig. 5A).

C/EBP β is composed of three isoforms: the liver-enriched activator protein LAP*, LAP, and the liver-enriched inhibitory protein LIP. A difference of 23 amino acids in the transactivation regions of LAP* and LAP gives rise to their distinct physiological roles [31,32]. Compared with the exogenously transfected *LAP*, LAP*, and *LIP* mRNAs, which were detected via RT‒PCR analysis, endogenous and exogenous LAP, LAP*, and LIP proteins were detected via Western blotting analysis in HeLa cells (Fig. 5B and C). Differential expression levels of *LAP*, LAP*, and *LIP* mRNAs were found, and caffeine further increased the expression of these three mRNAs, a finding consistent with the previously identified inhibitory effect on *HIF-1α* mRNA (Fig. 5B). Digoxin decreased the expression of *LAP* mRNA but increased the expression of *LIP* mRNA in HeLa cells (Fig. 5B). Endogenous LIP protein expression was suppressed in caffeine- or digoxin-treated HeLa cells (Fig. 5C). Caffeine decreased exogenous LAP* protein expression, and digoxin increased exogenous LAP protein expression. Consistent effects of caffeine and digoxin on the p53, HIF-1α, and p-eIF2α proteins were observed; however, there was no apparent change in the exogenous expression of the *LAP*, LAP*, and *LIP* mRNAs for the expression of *SRSF3* and its related targets (Fig. 5B and C).

**Figure 6:**
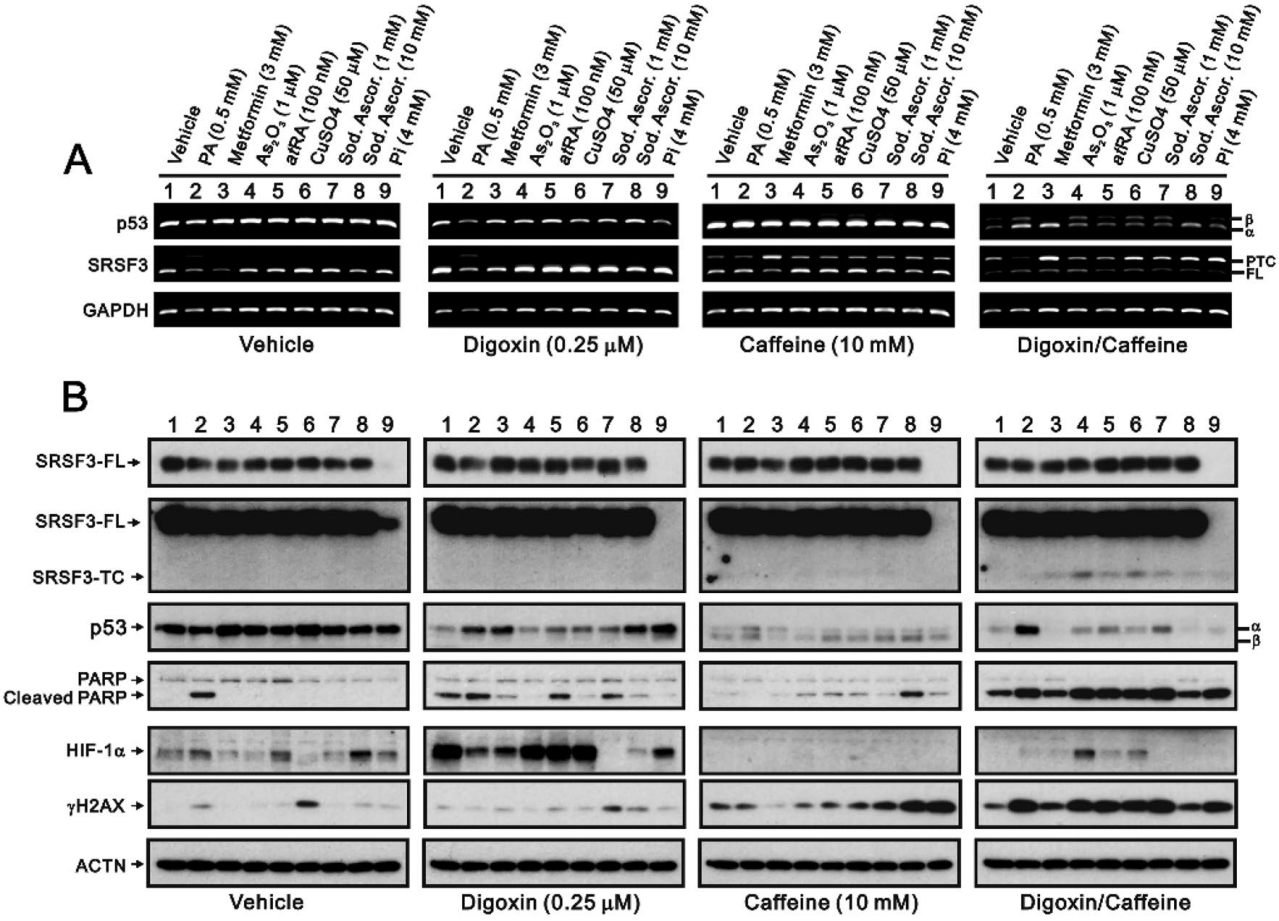
The effects of digoxin/caffeine combined with potential drugs on target genes and proteins in HeLa cells. HeLa cells were treated with 10 mM caffeine, 0.25 µM digoxin, or both in combination with the indicated drugs (PA: palmitic acid, metformin, As_2_O_3_: arsenic trioxide, atRA: all-trans retinoic acid, CuSO_4_: cupric sulfate, sod. Ascor: sodium ascorbate, Pi: phosphate ions) for 24 h. (**A**) mRNA was extracted and analyzed via RT‒PCR (with *GAPDH* as a loading control). (**B**) These cells were collected and subjected to immunoblot analysis for the indicated proteins and ACTN (a loading control). The results are representative of two independent experiments.

In addition to digoxin and caffeine, we screened many drugs of interest in the abovementioned luciferase reporter system. Primarily, we combined these drugs with digoxin and caffeine and then reconfirmed the effects of digoxin, caffeine, and both in combination on the isoform expression of *p53* and *SRSF3* mRNAs and proteins using RT‒PCR and Western blotting analysis (Fig. 6). Treatment with palmitic acid, metformin, or a pharmacological concentration of sodium ascorbate affected the *p53* and *SRSF3* mRNAs, and these effects were dependent on the presence of digoxin, caffeine, or a combination of both in HeLa cells (Fig. 6A). Western blot analysis revealed that phosphate ions completely suppressed the expression of the SRSF3 protein; we also revealed that the p53 protein was suppressed by digoxin (Fig. 6B). We further focused on the induction of cleaved PARP fragments (an apoptosis biomarker) and HIF-1α by digoxin and the induction of cleaved PARP fragments (an apoptosis biomarker) and γH2AX (a DNA damage biomarker; serine phosphorylation at residue 139) via the combination of digoxin and caffeine in HeLa cells. The induction of cleaved PARP fragments by digoxin was suppressed by metformin, arsenic trioxide, cupric sulfate, pharmacologically concentrated sodium ascorbate, and phosphate ions; moreover, the induction of HIF-1α by digoxin was differentially suppressed by palmitic acid, metformin, sodium ascorbate, and phosphate ions. The induction of cleaved PARP fragments and γH2AX by the combination of digoxin and caffeine was differentially suppressed by palmitic acid, metformin, arsenic trioxide, atRA, cupric sulfate, sodium ascorbate, and phosphate ions.

### The effects of phosphate ions and palmitic acid on the alternative splicing of full-length SRSF3 mRNA in HeLa cells

**Figure 7:**
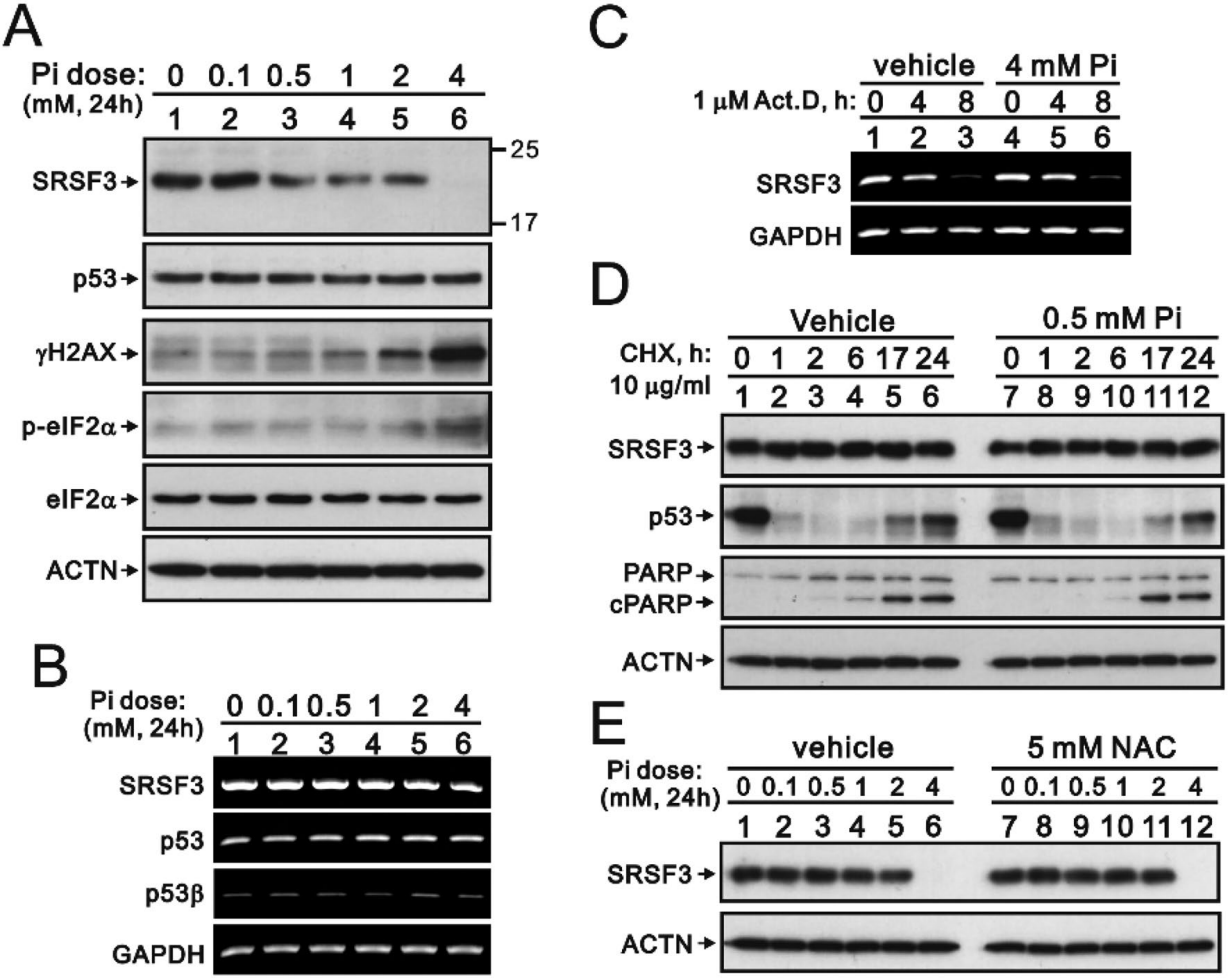
The effects of phosphate ions on the protein and mRNA stability of SRSF3 and p53 in HeLa cells. HeLa cells were treated with the indicated concentrations of phosphate ions for 24 h. (**A**) These cells were collected and subjected to immunoblot analysis for the detection of the indicated proteins and ACTN (a loading control). (**B**) mRNA was extracted and analyzed via RT‒PCR (with *GAPDH* as a loading control). (**C**) HeLa cells were treated with 4 mM phosphate ions plus 1 μM ActD for the indicated time for a total of 24 h. The mRNA was extracted and analyzed via RT‒PCR (with *GAPDH* as a loading control). (**D**) HeLa cells were treated with 0.5 mM phosphate ions plus 10 μg/ml CHX for the indicated times. (**E**) HeLa cells were pretreated with 5 mM NAC for 2 h and then treated with the indicated amount of phosphate ions for 24 h. These cells (**D** and **E**) were collected and subjected to immunoblot analysis for the detection of the indicated proteins and ACTN (a loading control).

We further reconfirmed the suppressive effect of phosphate ions on the expression of SRSF3 proteins. Our Western blotting data indicated that a reduction in the SRSF3 protein level was accompanied by an increase in the γH2AX protein level and the ER stress p-eIF2α/eIF2α ratio in a phosphate ion concentration-dependent manner (Fig. 7A). No apparent change in *SRSF3* mRNA expression was detected via RT‒PCR analysis (Fig. 7B). The protein and mRNA expression of p53 in HeLa cells was not disrupted by the addition of phosphate ions. We applied 1 μM of the de novo mRNA synthesis inhibitor actinomycin D (ActD) and 10 μg/ml CHX to assess the effect of phosphate ions on the stability of *SRSF3* mRNA and proteins, respectively (Fig. 7C and D). Our data showed that the stabilities of *SRSF3* mRNA and protein were not affected by the presence of phosphate ions in the HeLa cells. The effectiveness of CHX treatment was verified via the short half-life of the p53 protein and the induction of cleaved PARP protein. We further applied the reactive oxygen species (ROS) scavenger N-acetylcysteine (NAC) to confirm the effect of phosphate ions on the SRSF3 protein. Our data showed that NAC failed to reverse the effect of phosphate ions on the SRSF3 protein (Fig. 7E).

**Figure 8:**
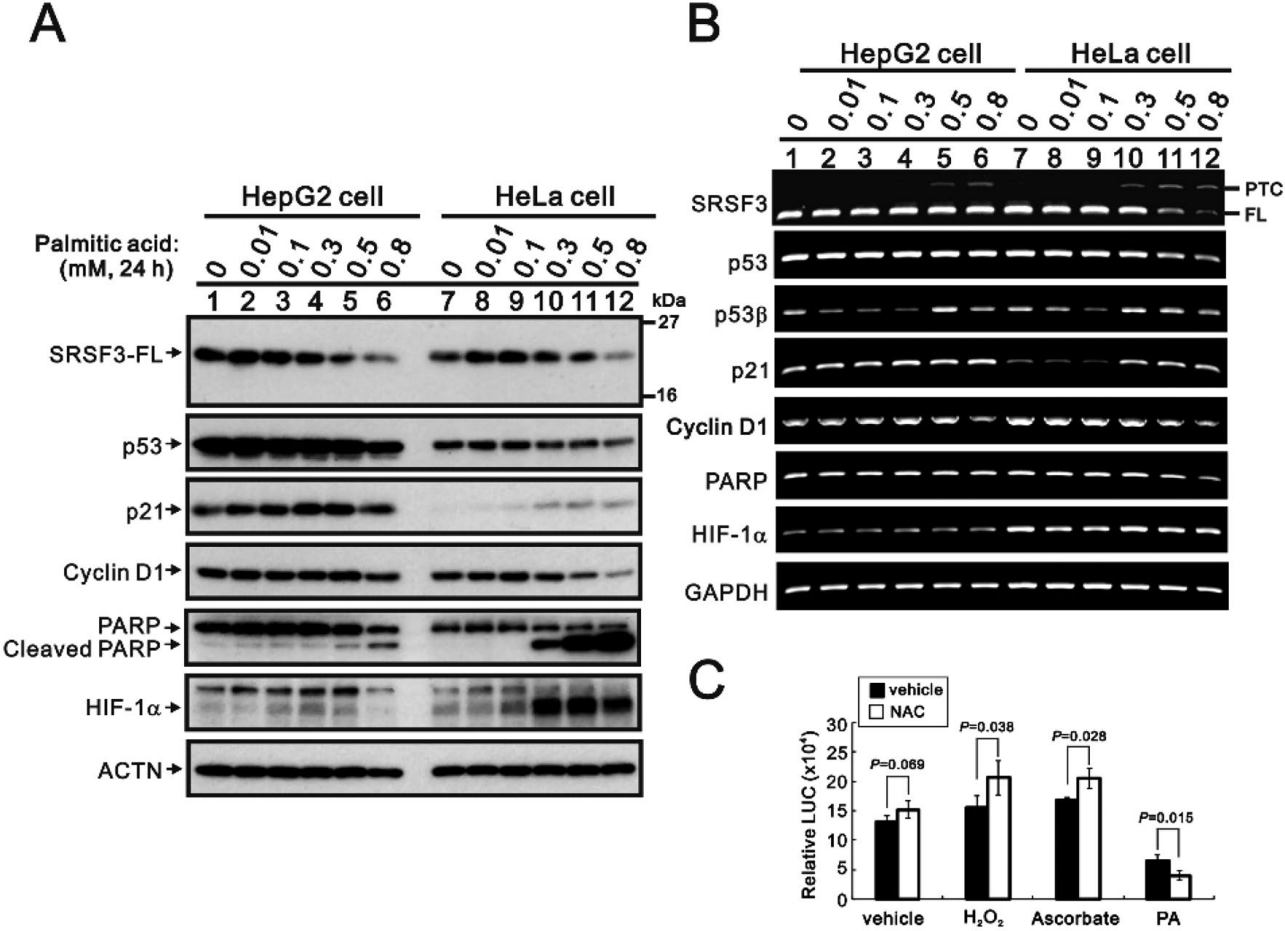
The effects of palmitic acid on the protein and mRNA stability of SRSF3-FL and SRSF3-TR in HepG2 and HeLa cells. HepG2 and HeLa cells were treated with the indicated concentrations of palmitic acid for 24 h. (**A**) These cells were collected and subjected to immunoblot analysis for the detection of the indicated proteins and ACTN (a loading control). (**B**) mRNA was extracted and analyzed via RT‒PCR (with *GAPDH* as a loading control). (**C**) HeLa cells were transfected with 0.5 μg of *SRSF3–LUC*; after 16 h, the cells were further treated with the indicated drugs for 24 h. Luciferase activities in the transfected cell extracts were then determined (RLU indicates relative light units). These data are the average of three independent experiments (mean ± S.D.; *n* = 3). The results of Student’s t tests were analyzed, and NAC was compared with vehicle for each group.

Palmitic acid has been reported to suppress the expression of SRSF3 proteins in hepatocytic carcinoma cells [30]. Here, we compared the effects of palmitic acid on the protein and mRNA levels of SRSF3, p53, p21, cyclin D1, and HIF-1α in HepG2 and HeLa cells. Palmitic acid decreased the protein expression of SRSF3, p53, and cyclin D1 and induced the cleavage of PARP fragments in a dose-dependent manner (Fig. 8A). However, palmitic acid induced the maximal expression of p21 and HIF-1α proteins at 0.5 mM but not at 0.8 mM. In the RT‒PCR analysis, we detected two *SRSF3* isoform mRNAs, both *full-length* and *PTC* mRNAs, in the HepG2 and HeLa cells treated with more than 0.3 mM palmitic acid (Fig. 8B). No *α-* or *β*-switched *p53* mRNAs were observed when either cell line was treated with palmitic acid. The regulation of *p21* and *cyclin D1* was found at the transcriptional level in both cell lines, but the regulation of *p53* at the transcriptional level was found only in HeLa cells (Fig. 8B). Compared with the change in *HIF-1α* mRNA, the increase in HIF-1α protein might be mediated via protein stability in HepG2 and HeLa cells (Fig. 8A and B). The *SRSF3* promoter activity was elevated by NAC treatment in the control, hydrogen peroxide, and sodium ascorbate groups (Fig. 8C). NAC failed to reverse the palmitic acid-induced suppression of *SRSF3* promoter activity.

## Discussion

SRSF3 is the smallest protein among the 12 classic SRSFs. This protein is responsible for regulating the alternative splicing of its own mRNA and autoregulating expression to maintain homeostasis^6,13,14^. SRSF3 has been shown to regulate the splicing of fibronectin^24^, pyruvate kinase M^25^, p53^15,16,20^, and glucose-6-phosphate dehydrogenase^26^. In addition to its role in RNA splicing regulation, SRSF3 is involved in almost all aspects of RNA biogenesis and processing, including gene transcription, RNA polyadenylation, export, stability, translation, and miRNA biogenesis. SRSF3 plays a role in a wide array of complex biological processes and is involved in cancer, aging, and neurological and cardiac disorders^7,26–29^. As a multifunctional RNA-binding protein and splicing factor, SRSF3 is subject to autoregulatory and balanced regulatory mechanisms that are essential for maintaining the proper levels of expression of the SRSF3-FL and SRSF3-TR proteins. Unfortunately, this balance is often disrupted in cancer. Therefore, the truncated protein must be produced under certain conditions and must fulfill a specific role. In this study, we first established a platform for coexpressing *SRSF3-FL* and *SRSF3-PTC* and further identified one specific antibody against the SRSF3-FL and SRSF3-TR proteins on the same gel. Next, we found that exogenously overexpressing *SRSF3-FL* or *SRSF3-PTC* failed to reverse the effects of digoxin, caffeine, or both in combination on its target genes, suggesting that the functions of the SRSF3-FL and SRSF3-TR proteins are distinct in HeLa cells. The proteasome inhibitor MG132 suppressed the exogenous and endogenous expression of SRSF3-FL and the exogenous expression of SRSF3-TR but induced endogenous SRSF3-TR expression. ER-related pathways, transcription factors such as LAP, and chemicals such as palmitic acid and phosphate ions were found to affect the regulation of *SRSF3* expression. The downregulation of SRSF3-FL by palmitic acid and phosphate ions was mediated via different regulatory mechanisms in HeLa cells. This study provides new insights into the altered expression of the SRSF3-FL and SRSF3-TR proteins for the identification of the functions of SRSF3 in cells.

The role of SRSF3 protein stability in cells is different. The stability of SRSF3 proteins is regulated via ubiquitination or neddylation for degradation by the proteasome complex^30^. Cellular stress can promote the neddylation of SRSF3 at Lys11 via palmitic acid, which targets it for degradation by the proteasome complex. SRSF3 has at least two ubiquitinated sites, Lys23 and Lys85, and three neddylation sites, Lys11, Lys23, and Lys85. Neddylation in SRSF3 Lys85 is also responsible for stress granule assembly^31^. We found different results for the involvement of SRSF3-FL and SRSF3-TR protein stability in cells. One ubiquitin-dependent proteasome inhibitor, MG132, stabilized the SRSF3-TR protein but not the SRSF3-FL protein in our current study. Compared with the functions of individual SRSF3-FL and SRSF3-TR proteins, it is difficult to demonstrate the exact functions of SRSF3 proteins at various ratios of SRSF3-FL/SRSF3-TR. However, it would be interesting to study the functional roles of various ratios of SRSF3-FL/SRSF3-TR in the so-called SFSF3 functions of cancer cells. SRSF3 has been identified as a polyfunctional protein that is involved in multiple physiological and pathological processes^32^. The overexpression of SRSF3 or a degradation-resistant mutant was sufficient to prevent progressive liver disease in mice receiving a nonalcoholic steatohepatitis (NASH)-inducing diet^30^. A recent study showed that the downregulation of SRSF3 via an antisense strategy sensitized oral squamous cell carcinoma and breast cancer cells to the chemotherapeutic drug paclitaxel^33^.

A recent study conducted by Dr. You's laboratory revealed that cardiac glycosides can exert inhibitory effects on the NMD pathway by binding to and inhibiting sodium–potassium ATPase on the plasma membrane^34^. While NMD can be beneficial in preventing the production of C-terminally truncated proteins with a dominant negative function, there are cases where the truncated protein encoded by the PTC-containing mRNA still has some residual function, and the NMD-mediated reduction in mRNA abundance results in more severe clinical problems^35–37^. Mechanistically, NMD is often self-regulated, and the presence of multiple elements that render NMD factor-encoding mRNAs natural substrates of NMD suggests that several central factors of NMD are regulated by NMD^38^. However, correlating NMD efficacy with the levels of transcripts that encode the main NMD factors is a challenge^39^. ER stress can reversely regulate NMD such that, when ER stress becomes too severe, NMD loses its ability to inhibit the stress response, ultimately leading to NMD inhibition^40^. For confirmation that high levels of cytoplasmic calcium inhibit NMD, an ionophore was used to induce calcium release from the ER into the cytoplasm in human U2OS cells^34^. Although the exact mechanism by which calcium interferes with the NMD machinery is yet to be understood, the failure of NMD leads to the accumulation of aberrant mRNAs, which provides cancer cells with a selective advantage. The long SRSF3 isoform, which contains exon 4, is primarily degraded by NMD, and only a fraction of this isoform encodes a SRSF3-TR protein that survives NMD. The autoregulatory properties of SRSF3 indicate that accounting for both the SRSF3-FL and SRSF3-TR proteins is crucial for elucidating the final functions of SRSF3 in physiological and pathological processes^12,30^. Additionally, the clinical drugs that modulate the exact ratio of SRSF3-FL and SRSF3-TR proteins might do so through autoregulation, NMD, ER stress, calcium hemostasis, transcription factors, and unidentified factors. In this study, when cells were treated with digoxin, caffeine, or other chemicals utilized in our other publications, we detected the stabilization of *SRSF3-PTC* mRNA and an increase in the amount of SRSF3-TR protein^15–18^. Given the critical roles of alternative splicing in cancer biology, pharmacologically modulating SRSF3-mediated splicing might be an essential therapeutic strategy.

SRSF3 can shuttle between the nucleus and cytoplasm, facilitating mRNA export and stability in the cytoplasm. When SRSF3 remains in the nucleus, it primarily participates in RNA splicing at active sites of RNA polymerase II transcription. Any disturbance of the expression and modification of the SRSF3-FL and SRSF3-TR proteins can contribute to the disruption of normal cell functions and lead to pathological outcomes. However, most publications demonstrate the functional role of the SRSF3-FL protein while neglecting the function of the SRSF3-TR protein mediated via genetic and pharmacological strategies^29,30,41–44^. In our study, we aimed to elucidate the inclusion mechanism of exon 4 in *SRSF3* mRNA splicing and found that phosphate ions decreased the amount of SRSF3-FL protein without increasing the inclusion of exon 4 in *SRSF3* mRNA (*SRSF3-PTC*). In contrast, palmitic acid increased the amount of *SRSF3-PTC* and decreased the amount of SRSF3-FL protein in HeLa cells. Our findings suggest that the amount of SRSF3-FL protein is not the only factor involved in analyzing the inclusion of exon 4 in *SRSF3* mRNA splicing. Therefore, the status of autoregulation and NMD might determine the fate of *SRSF3-PTC* and further indicate whether functional SRSF3-TR protein is produced in cells.

In summary, SRSF3 is involved in regulating the alternative splicing of its own mRNA and autoregulating its expression to maintain homeostasis. The exon skipping of *SRSF3* leads to the synthesis of a truncated protein rather than a PTC-generating frameshift mutation for degradation by NMD. In our study, we first established a platform for coexpressing *SRSF3-FL* and *SRSF3-PTC*, and exogenously overexpressing *SRSF3-FL* and *SRSF3-PTC* failed to preserve the effects of digoxin, caffeine, or a combination of both on SRSF3 and its targets. The proteasome inhibitor MG132 suppressed exogenous and endogenous SRSF3-FL expression and exogenous SRSF3-TR expression but induced endogenous SRSF3-TR expression. In ER-related pathways, transcription factors such as LAP and chemicals such as palmitic acid and phosphate ions are involved in regulating *SRSF3* expression. Hence, our study provides new insights into the use of switched expression of the SRSF3-FL and SRSF3-TR proteins to determine the functions of SRSF3 proteins in cells.

## Materials and methods

### Cell culture, transfection, luciferase reporter assay, and reagents

HeLa and HepG2 cells were obtained from the American Type Culture Collection (ATCC; Manassas, VA, USA) and were cultured in DMEM (Corning, Corning, NY, USA) supplemented with 10% FBS and 1% penicillin–streptomycin (Invitrogen, Waltham, MA, USA). HeLa cells were plated into 6-well culture plates and transiently transfected with jetPEI (PolyPlus Transfection, Inc., New York, NY) following the manufacturer's protocol. The total amount of transfected DNA was adjusted to 1 µg via the addition of the empty vector. For the luciferase reporter assay, HeLa cells were plated into 24-well culture plates, and the Promega Luciferase Assay Kit was used to perform the assay. The values are expressed numerically as relative light units (RLUs). The luciferase activity is presented as the mean ± SD of three transfected wells and is representative of at least three independent experiments. Act D, caffeine, CHX, cupric sulfate, digoxin, MG132, NAC, palmitic acid, and sodium ascorbate were purchased from Sigma-Aldrich (St. Louis, MO, USA).

### Western blotting analysis

Cells were lysed with lysis buffer containing 100 mM Tris–HCl (pH 8.0), 150 mM NaCl, 0.1% SDS, and 1% Triton 100 at 4 °C. The protein concentrations in the lysates were measured using the Bio-Rad Protein Assay Dye Reagent Concentrate (Bio-Rad Laboratories, USA). The protein lysate was then prepared with 4 × protein loading dye, denatured at 95 °C for 10 min, separated via 12% SDS‒PAGE, and blotted onto PVDF membranes (Immobilon-P; Millipore, Bedford, MA, USA) using a Bio-Rad Semi-Dry Transfer Cell. The blots were then incubated with primary antibodies against α-actinin (ACTN, H-2, sc-17829), p53 (DO-1, sc-125), c-Jun (H-79, sc-1694), XBP-1 (F-4, sc-8015), and C/EBPβ (H-7, sc-7962) (Santa Cruz Biotechnology, USA); cleaved PARP (#9546), p-eIF2α (#9721), eIF-2α (#9722), Upf-1 (#12,040), and HIF-1α (#14,179) (Cell Signaling, Danvers, MA, USA); cyclin D1 (ab134175), p21 (ab109520), and γH2AX (ab81299) (Abcam, Cambridge, UK); SRSF3 (WH0006428M8, Sigma‒Aldrich); and HA (clone 3F10, Roche). The blots were subsequently incubated with HRP-conjugated secondary antibodies (anti-mouse IgG, AP192P; and anti-rabbit IgG, AP132P; Merck-Millipore). The immunoreactive proteins were detected using the ECL^TM^ Western Blotting Detection Reagent and Amersham Hyperfilm^TM^ ECL (GE Healthcare, USA).

### Construction of plasmids

The *SRSF3-FL* (wild-type) and *SRSF3-PTC* (truncated form) coding regions were synthesized via polymerase chain reaction (PCR) and subcloned and inserted into the EcoRI and SalI sites of pSG5.HA vector. The DNA sequences of the *SRSF3-FL* PCR primers (495 bp) used were as follows: forward primer, 5′-AAGAATTCATGCATCGTGATTCCTGTCCATTG-3′; reverse primer, 5′-AAGTCGACCTATTTCCTTTCATTTGACCTAGATC-3′; and *SRSF3-PTC* PCR primers (375 bp), forward primer, 5′-AAGAATTCATGCATCGTGATTCCTGTCCATTG-3′; reverse primer, 5′-AAGTCGACTCAGAGGGTGGTGAGAAGAGACATG-3′. The promoter of *SRSF3* (-1650/ + 171) was synthesized via PCR and subcloned and inserted into the KpnI and HindIII sites of the pGL3-LUC vector (Promega Co., Madison, WI, U.S.A.). The sequences of the *SRSF3-LUC* PCR primers used were as follows: forward, 5′-AAGGTACCAGAGGAGGGGAACAAATCAAGGC-3′; reverse, 5′-AAAAGCTTTTCGAGATCTAGGGTTAAAAAATGC-3′. The *LAP*, LAP*, and *LIP* coding regions were synthesized via PCR and subcloned and inserted into the EcoRI and XhoI sites of pSG5.HA vector. The DNA sequences of the *LAP*, LAP*, and *LIP* PCR primers used were as follows: LAP forward primer: 5′-AAGAATTCATGCAACGCCTGGTGGCCTGGGA-3′; LAP* forward primer: 5′-AAGAATTCATGGAAGTGGCCAACTTCTACTACG-3′; LIP forward primer: 5′-AAGAATTCATGGCGGCGGGCTTCCCGTACG-3′; and *LAP*, LAP*, and *LIP* reverse primer: 5′-AACTCGAGCTAGCAGTGGCCGGAGGAGGCG-3′. The *SOX9* coding regions were synthesized via PCR and subcloned and inserted into the EcoRI and XhoI sites of the pSG5.HA vector. The DNA sequence of the *SOX9* PCR primers was as follows: forward primer, 5′-AAGAATTCATGAATCTCCTGGACCCCTTCATG-3′; reverse primer, 5′-AACTCGAGTCAAGGTCGAGTGAGCTGTGTGTAG-3′. The *HOXA5* coding regions were synthesized via PCR and subcloned and inserted into the EcoRI and XhoI sites of pSG5.HA vector. The DNA sequence of the *HOXA5* PCR primers was as follows: forward primer, 5′-AAGAATTCATGAGCTCTTATTTTGTAAACTCATTTT-3′; reverse primer, 5′-AACTCGAGTCAGGGACGGAAGGCCCC-3′.

### Reverse transcriptase–polymerase chain reaction (RT‒PCR)

Total RNA was extracted from the HeLa and HepG2 cells using TRI Reagent Solution (Invitrogen, Thermo Fisher Scientific) following the manufacturer's instructions. Total RNA (1 μg) was then subjected to reverse transcription for 60 min at 42 °C using M-MuLV reverse transcriptase (Protech Technology Enterprise Co., Ltd.). The PCRs were run on a Veriti Thermal Cycler (Applied Biosystems, USA), and the PCR sequences of primers used are shown in Table 1.

**Table 1:** Primers used in this study.

Gene name	Primer sequence (5′→3′)
*SRSF3*	Forward: 5′-ATGCATCGTGATTCCTGTCCATTG-3′ Reverse: 5′-CTATTTCCTTTCATTTGACCTAGATC-3′
*GAPDH*	Forward: 5′-CTTCATTGACCTCAACTAC-3′ Reverse: 5′-GCCATCCACAGTCTTCTG-3′
*p53*	Forward: 5′-CTCTGACTGTACCACCATCCACTA-3′ Reverse: 5′-GAGTTCCAAGGCCTCATTCAGCTC-3′
*p21*	Forward: 5′-CTGAGCCGCGACTGTGATGCG-3′ Reverse: 5′-GGTCTGCCGCCGTTTTCGACC-3′
*Cyclin D1*	Forward: 5′-ATGGAACACCAGCTCCTGTGCTGC-3′ Reverse: 5′-TCAGATGTCCACGTCCCGCACGTCGG-3′
*HIF-1α*	Forward: 5′-GAACCTGATGCTTTAACT-3′ Reverse: 5′-CAACTGATCGAAGGAACG-3′
*XBP-1*	Forward: 5′-CCTGGTTGCTGAAGAGGAGG-3′ Reverse: 5′-CCATGGGGAGATGTTCTGGAG-3′
*C/EBPβ*	Forward: 5′-ATGGCGGCGGGCTTCCCGTACG-3′ Reverse: 5′-CTAGCAGTGGCCGGAGGAGGCG-3′
*p53β*	Forward: 5′-ATGGAGGAGCCGCAGTCAGAT-3′ Reverse: 5′-TTTGAAAGCTGGTCTGGTCCTGA-3′
*PARP*	Forward: 5′-CAGAAGTACGTGCAAGGGGT-3′ Reverse: 5′-GGCACTTGCTGCTTGTTGAAG-3′

### Statistical analyses

The values are expressed as the mean ± SD of at least three independent experiments. All comparisons between groups were conducted using Student’s *t* tests with SPSS 20.0 for Windows (SPSS, Chicago, IL). The statistical significance was set at *p* < 0.05.

### Supplementary Information

Supplementary Figures.

## Data Availability

The data generated during the current study are available from the corresponding author upon reasonable request.
