# Supplementary material for: Factors affecting the expression and stability of full-length and truncated SRSF3 proteins in human cancer cells
Source: Sci Rep. 2024 Jun 22;14:14397. doi: 10.1038/s41598-024-64640-1 (PMC11193772; doi:10.1038/s41598-024-64640-1)
Supplement: Supplementary file 1 — Supplementary Figures. [file 41598_2024_64640_MOESM1_ESM.pdf]

Figure 1B:

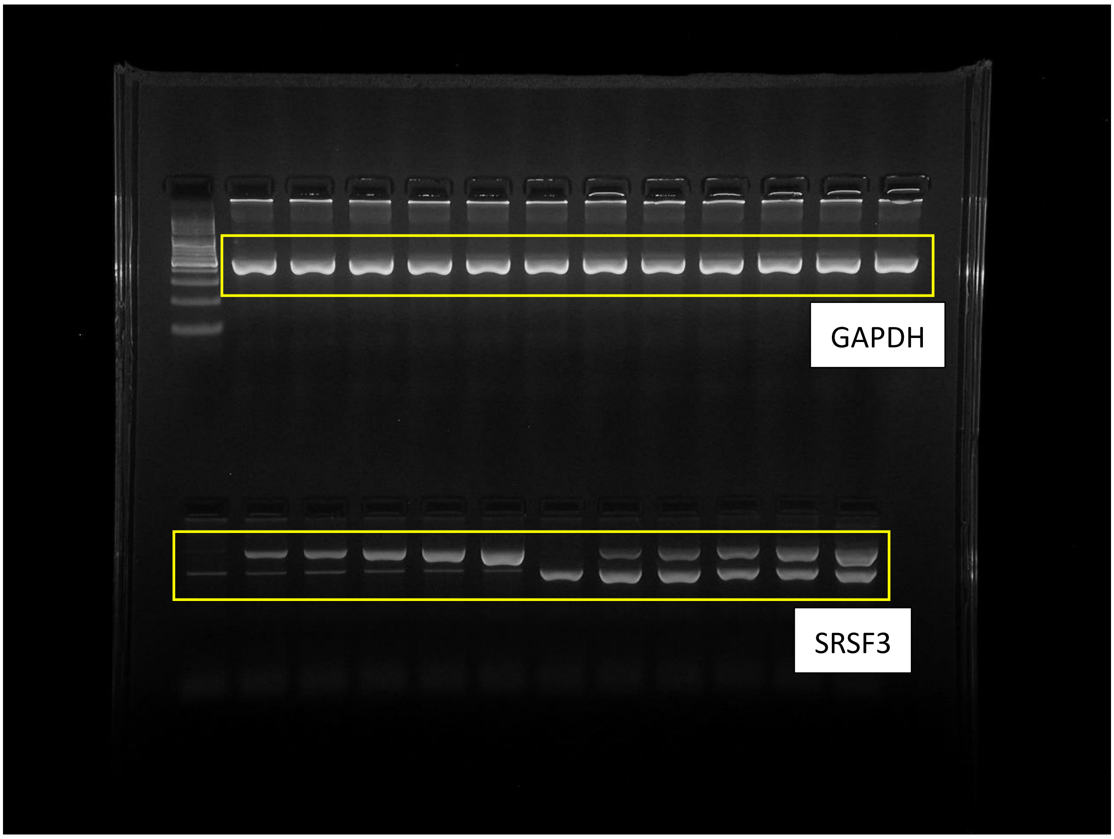

Figure 1C:

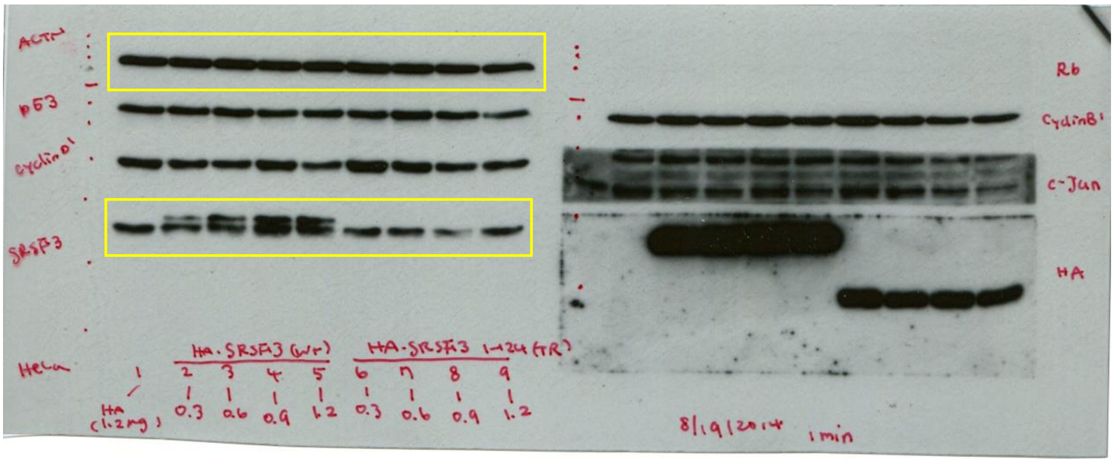

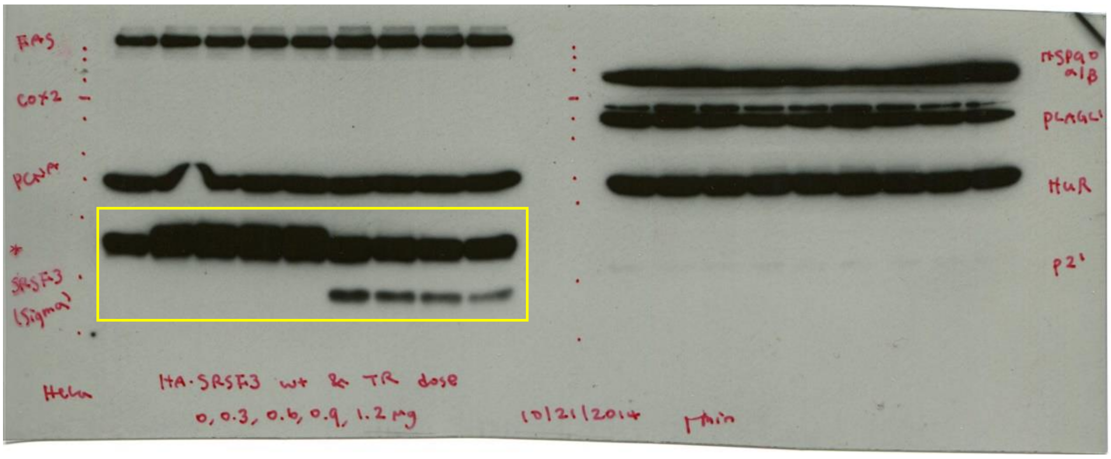
FAS
Cox2
PCNA
SRSF3 (Sigma)
HSP90 α/β
PLAGL1
HuR
p21
Hela
HA-SRSF3 wt & TR dose
0, 0.3, 0.6, 0.9, 1.2 μg
10/21/2014

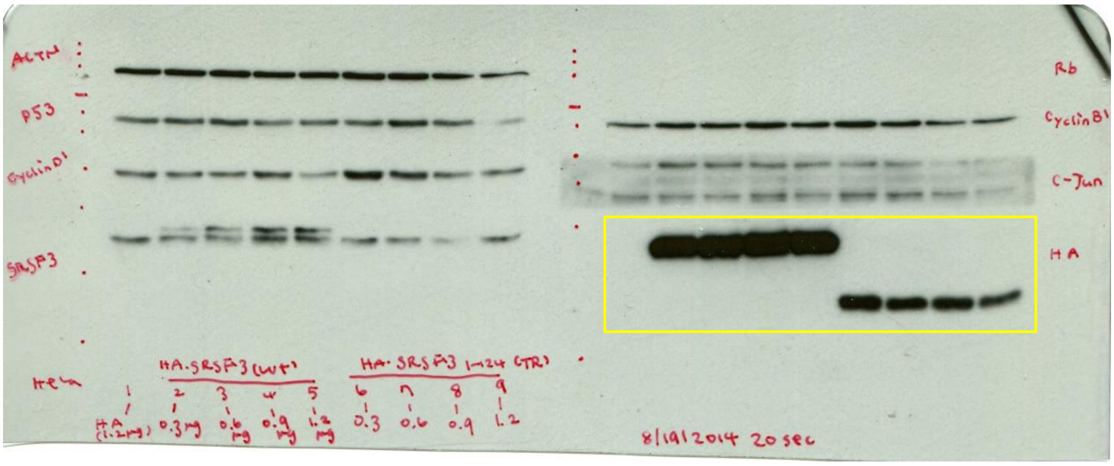
ACTN
P53
CyclinD1
SRSF3
Rb
CyclinB1
C-Jun
HA
Hela
HA-SRSF3 (WT)
HA-SRSF3 1-124 (TR)
1 2 3 4 5 6 7 8 9
8/19/2014 20 sec

Figure 1D:

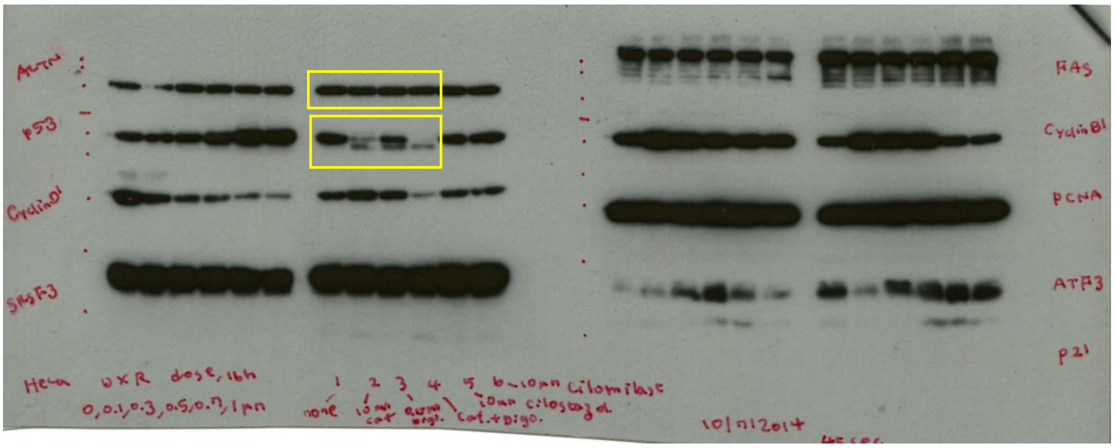
ACTN
p53
CyclinD1
SRSF3
FAS
CyclinB1
PCNA
ATF3
p21
Hela
10/7/2014

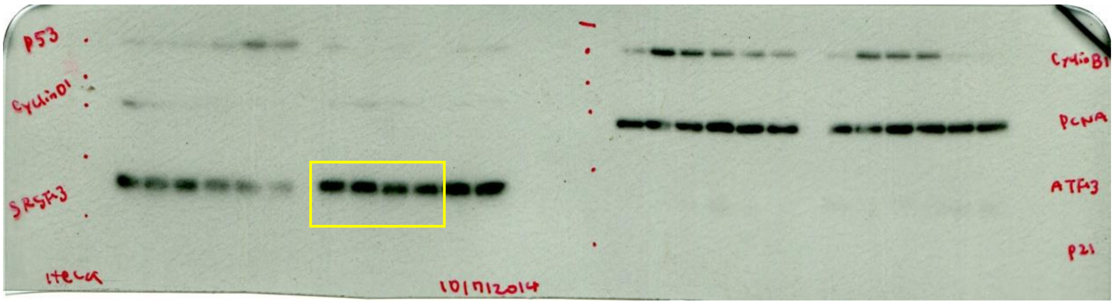
P53
CyclinD1
SRSF3
CyclinB1
PCNA
ATF3
p21
Hela
10/7/2014

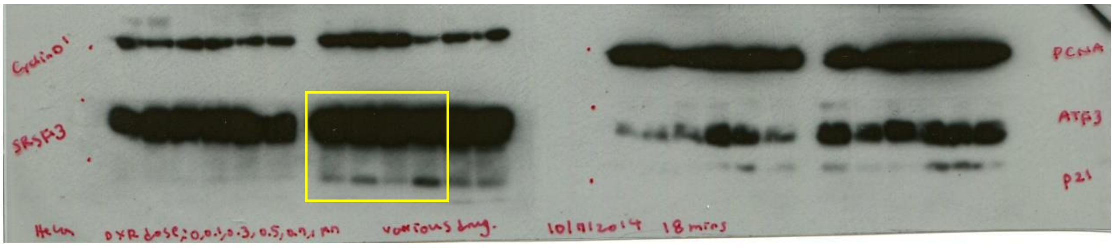
Cyclin D1
SRSF3
PCNA
ATF3
p21
HeLa DXR dose; 0, 0.1, 0.3, 0.5, 0.7, 1 nM various drug.
10/9/2014 18 mins

Figure 2A:

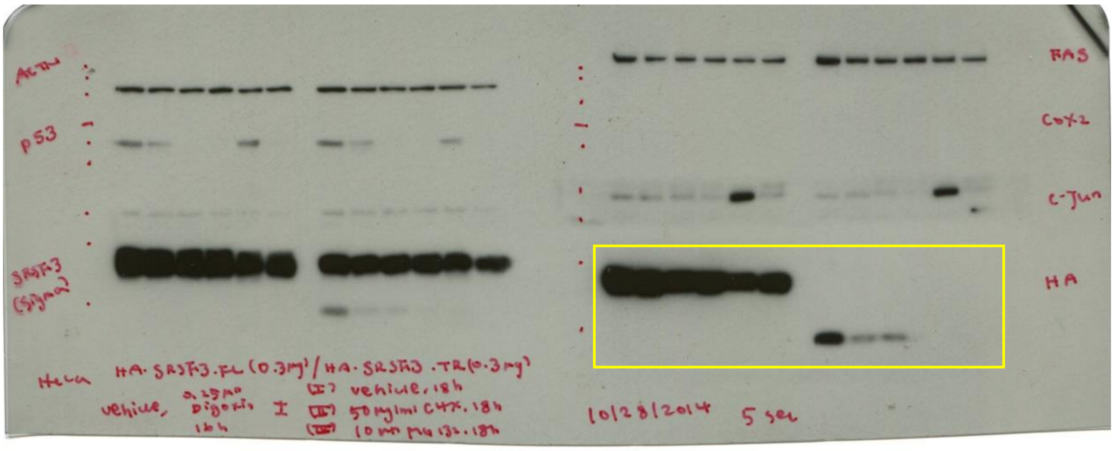
ACTN
p53
SRSF3 (Sigma)
FAS
Cox-2
C-Jun
HA
HeLa HA-SRSF3-FL (0.3 mg) / HA-SRSF3-TR (0.3 mg)
vehicle, 0.25 nM Digoxin 16 h ± (I) vehicle, 18 h (II) 50 mg/ml CHX, 18 h (III) 10 nM MG132, 18 h
10/28/2014 5 sec

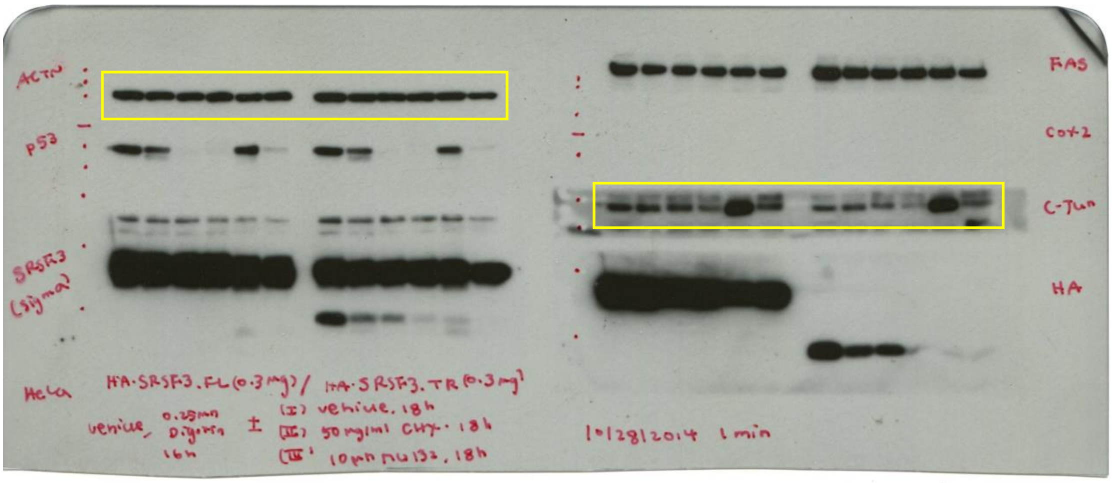
ACTN
p53
SRSF3 (Sigma)
FAS
Cox-2
C-Jun
HA
HeLa HA-SRSF3-FL (0.3 mg) / HA-SRSF3-TR (0.3 mg)
vehicle, 0.25 nM Digoxin 16 h ± (I) vehicle, 18 h (II) 50 mg/ml CHX, 18 h (III) 10 nM MG132, 18 h
10/28/2014 1 min

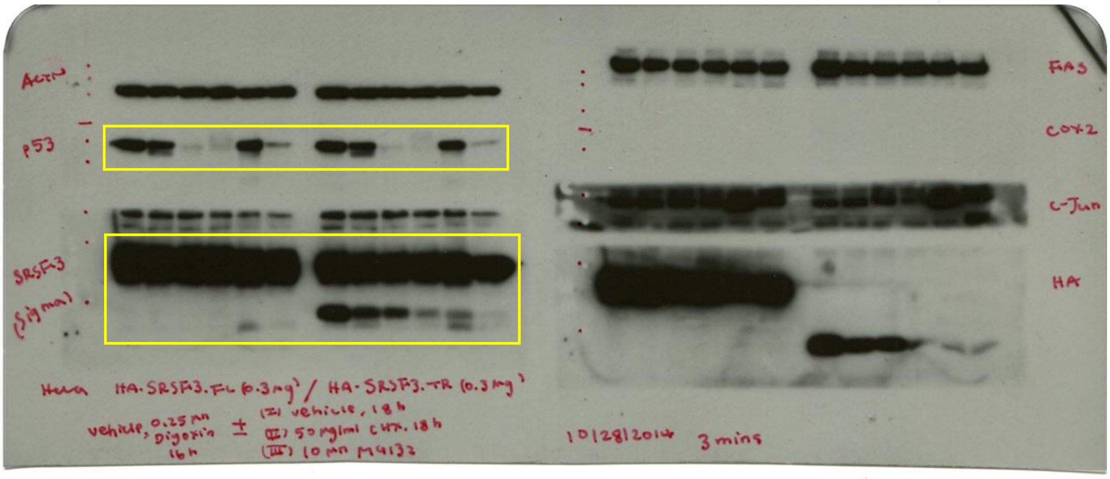
ACTN
p53
SRSF3 (Sigma)
FAS
COX-2
C-Jun
HA
HeLa HA-SRSF3-FL (0.3 mg) / HA-SRSF3-TR (0.3 mg)
vehicle, 0.25 nM Digoxin 16 h ± (I) vehicle, 18 h (II) 50 mg/ml CHX, 18 h (III) 10 nM MG132
10/28/2014 3 mins

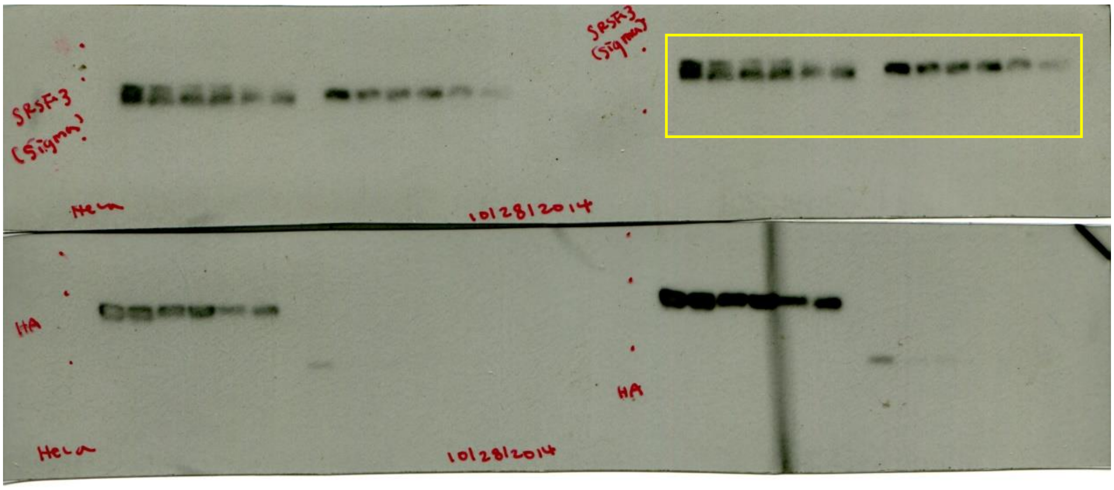
SRSF3 (Sigma)
HeLa
10/28/2014
SRSF3 (Sigma)
HA
HeLa
10/28/2014
HA

Figure 2B:

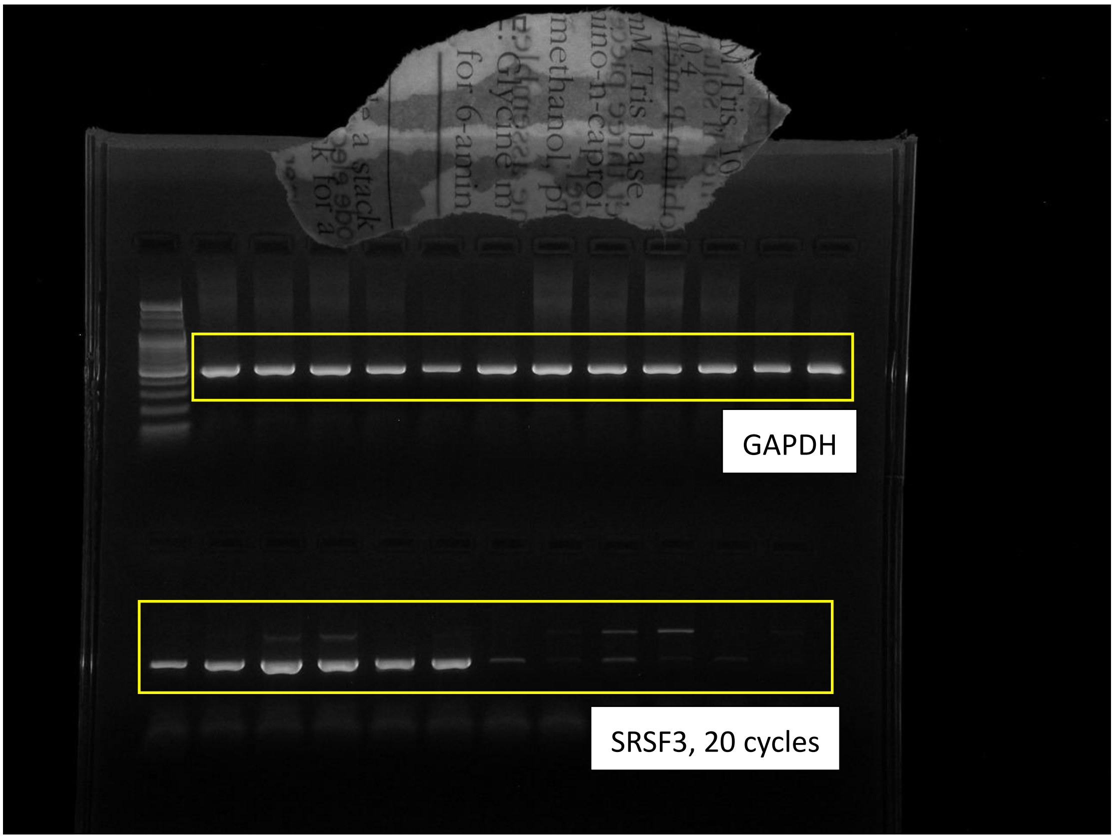
GAPDH
SRSF3, 20 cycles

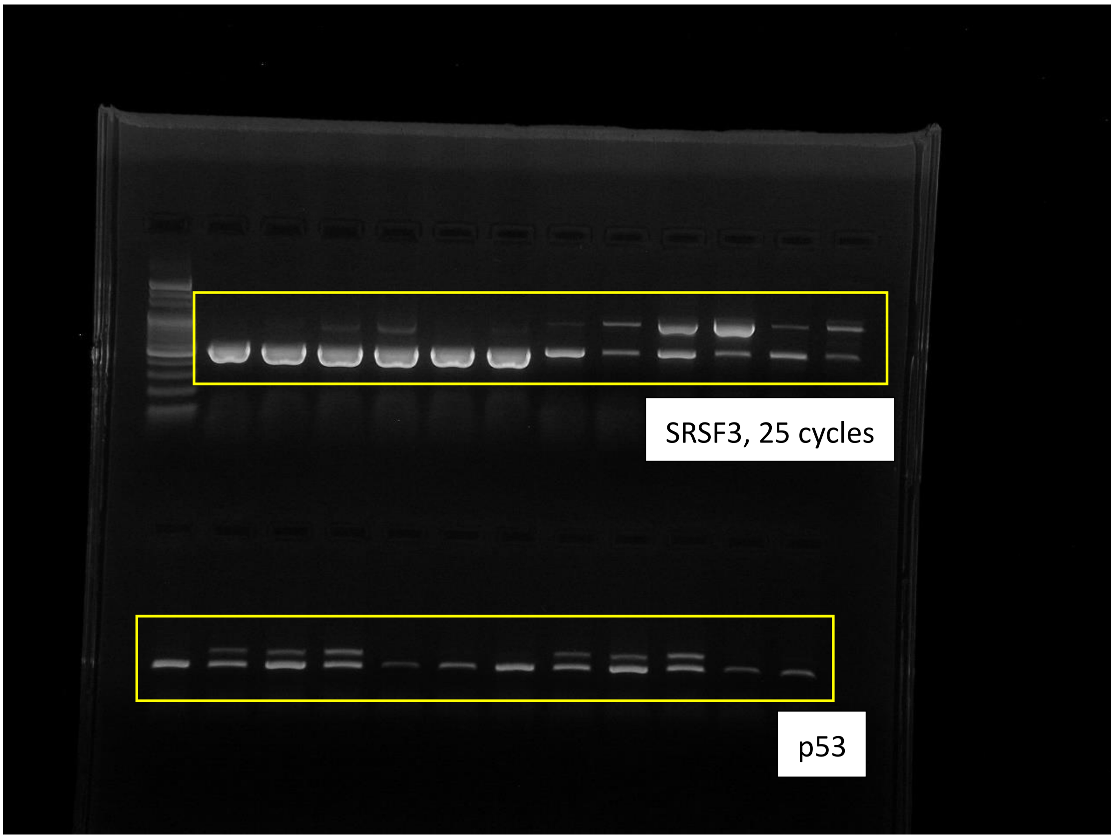
SRSF3, 25 cycles
p53

Figure 3A:

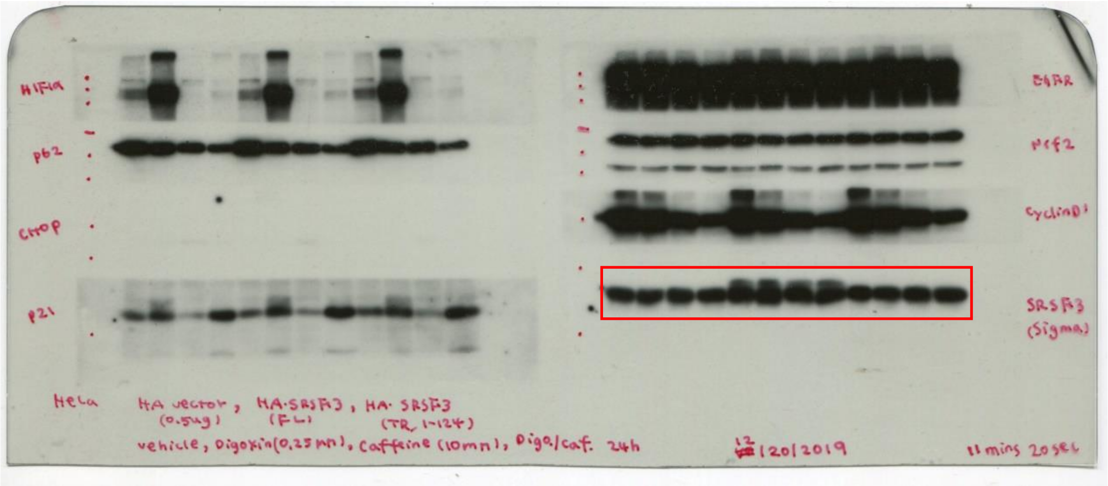
p62
CHOP
p21
Nrf2
SRSF3
(Sigma)
HeLa
HA vector,
(0.5ug)
HA-SRSF3,
(FL)
HA-SRSF3
(TR, 1-124)
vehicle, Digoxin(0.25 mM), Caffeine (10mM), Digo/caf. 24h
12/20/2019

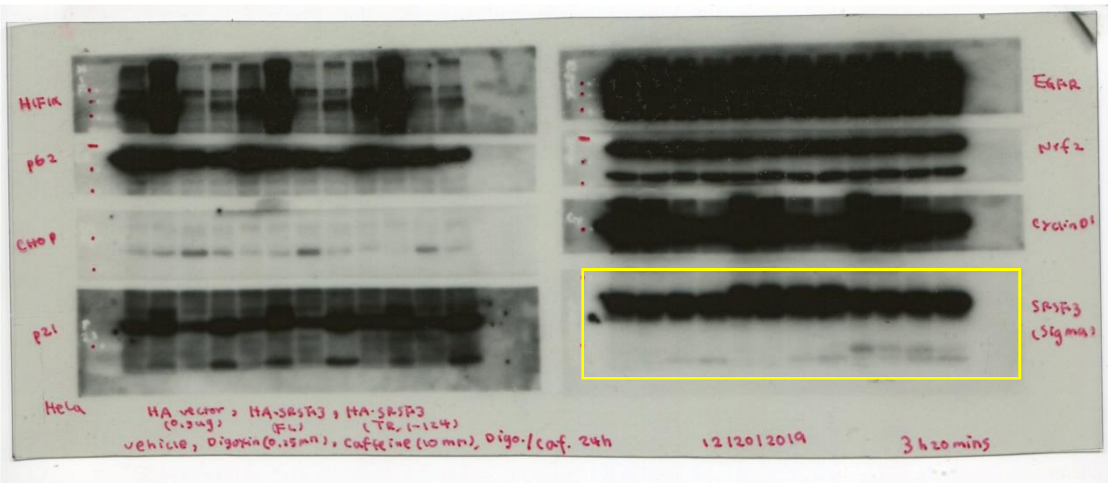
HIF1α
p62
CHOP
p21
EGFR
Nrf2
cyclinD1
SRSF3 (Sigma)
Hela
HA vector, HA-SRSF3, HA-SRSF3
(0.5ug) (FL) (TR, 1-124)
vehicle, Digoxin (0.25mM), caffeine (10mM), Digo./caf. 24h
12/20/2019
3h20mins

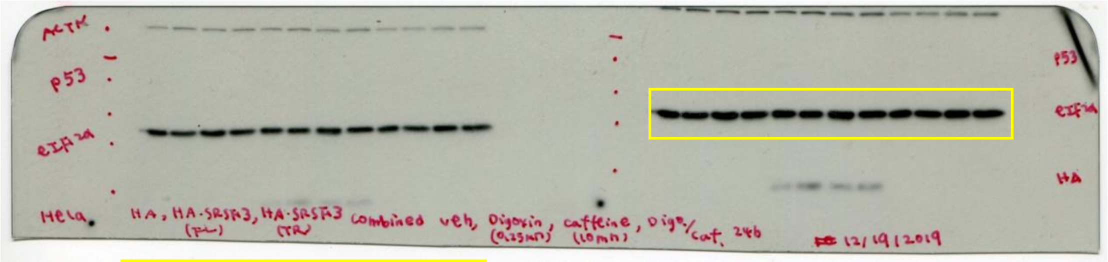
ACTN
p53
eIF2α
p53
eIF2α
HA
Hela
HA, HA-SRSF3, HA-SRSF3 combined veh, Digoxin, caffeine, Digo./caf. 24h
(FL) (TR) (0.25mM) (10mM)
12/19/2019

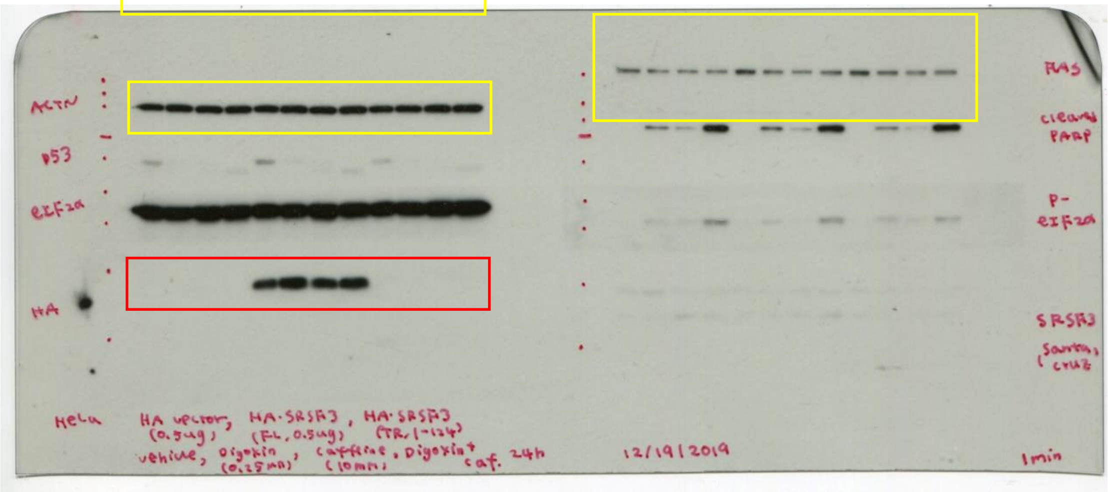
ACTN
p53
eIF2α
HA
FAS
cleaved PARP
P-eIF2α
SRSF3 (Santa cruz)
Hela
HA vector, HA-SRSF3, HA-SRSF3
(0.5ug) (FL, 0.5ug) (TR, 1-124)
vehicle, Digoxin, caffeine, Digoxin+caf. 24h
(0.25mM) (10mM)
12/19/2019
1min

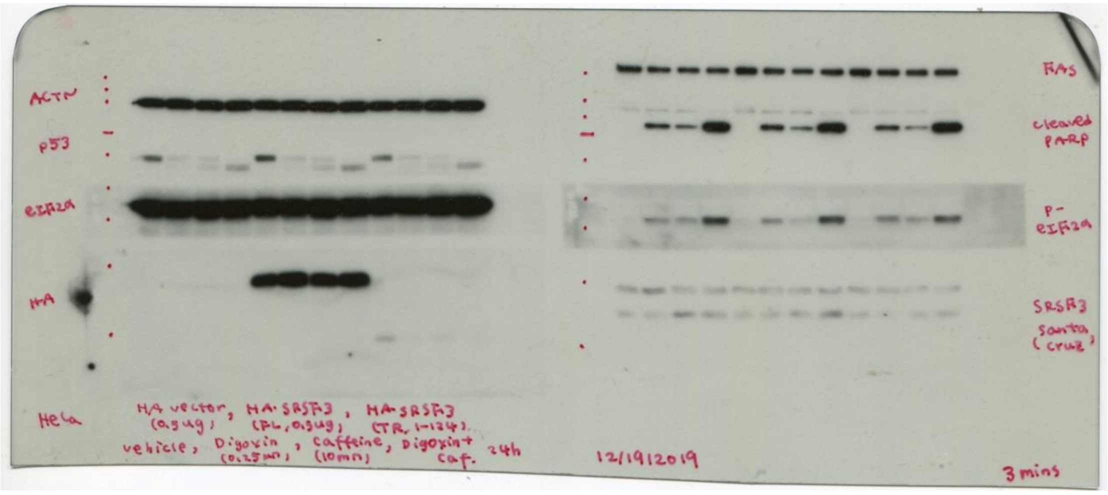
ACTN
p53
eIF2α
HA
FAS
cleaved PARP
P-eIF2α
SRSF3 Santa cruz
Hela
HA vector, HA-SRSF3, HA-SRSF3
(0.5ug) (FL, 0.5ug) (TR, 1-124)
vehicle, Digoxin, caffeine, Digoxin+caf. 24h
(0.25mM) (10mM)
12/19/2019
3mins

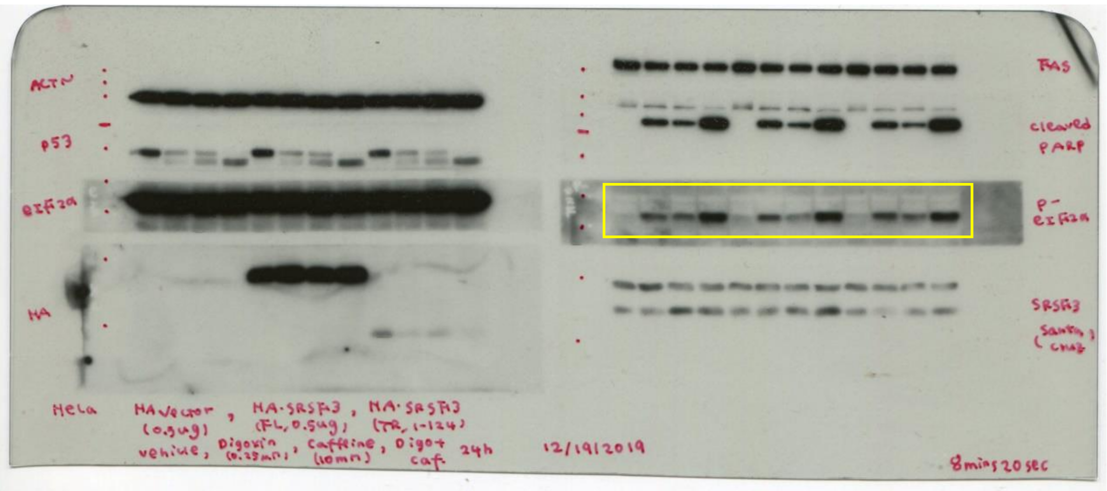
ACTN
p53
eEF2α
HA
FAS
cleaved PARP
p-eEF2α
SRSF3 (Santa Cruz)
HeLa
HA vector (0.5ug), HA-SRSF3 (FL, 0.5ug), HA-SRSF3 (TR, 1-124)
vehicle, Digoxin (0.25uM), Caffeine (10mM), Digo+caf. 24h
12/19/2019
8mins 20 sec

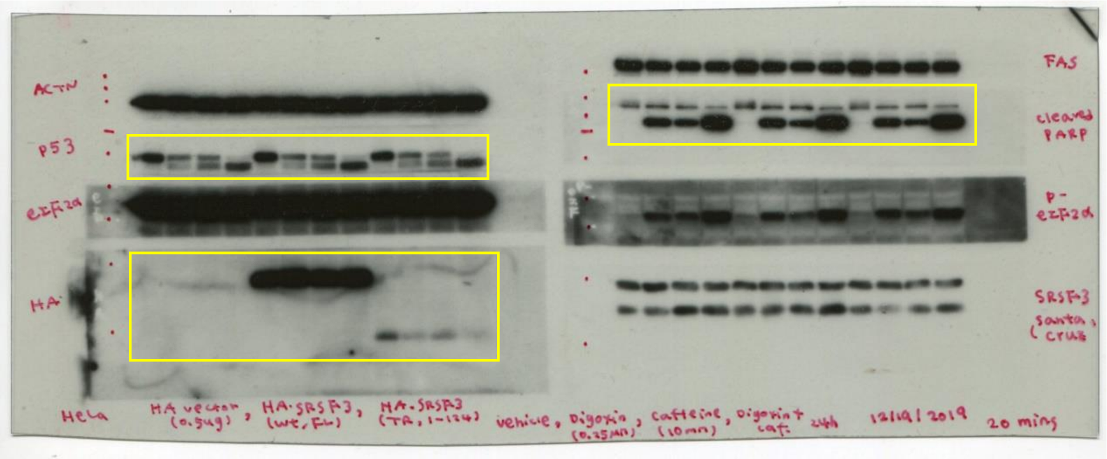
ACTN
p53
eEF2α
HA
FAS
cleaved PARP
p-eEF2α
SRSF3 (Santa Cruz)
HeLa
HA vector (0.5ug), HA-SRSF3 (wt, FL), HA-SRSF3 (TR, 1-124)
vehicle, Digoxin (0.25uM), Caffeine (10mM), Digoxin+caf. 24h
12/19/2019
20 mins

Figure 3B:

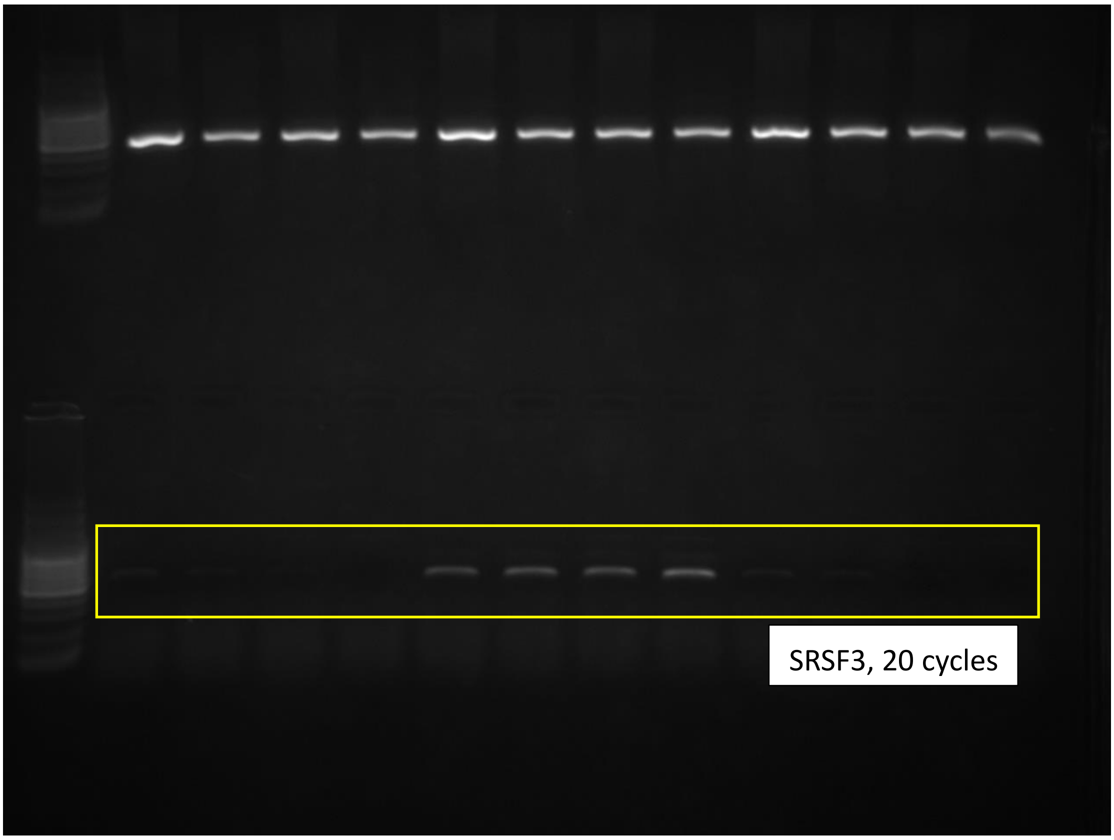
SRSF3, 20 cycles

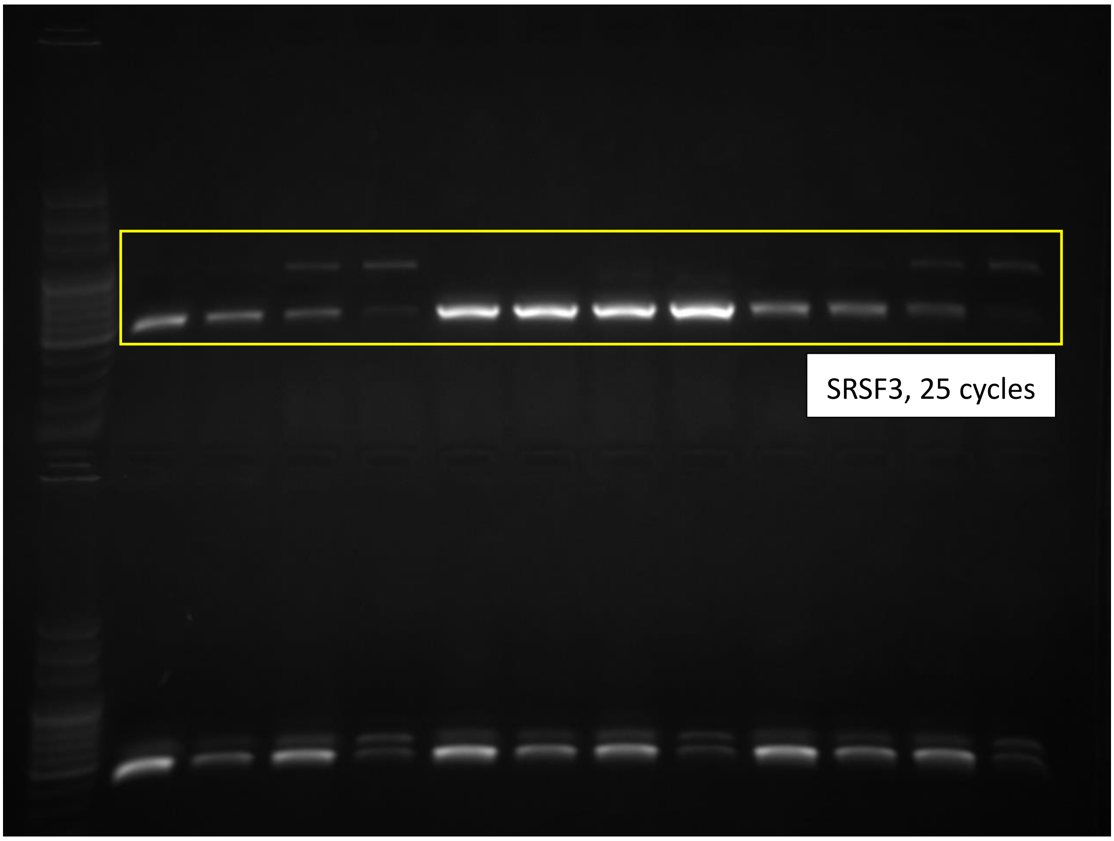
SRSF3, 25 cycles

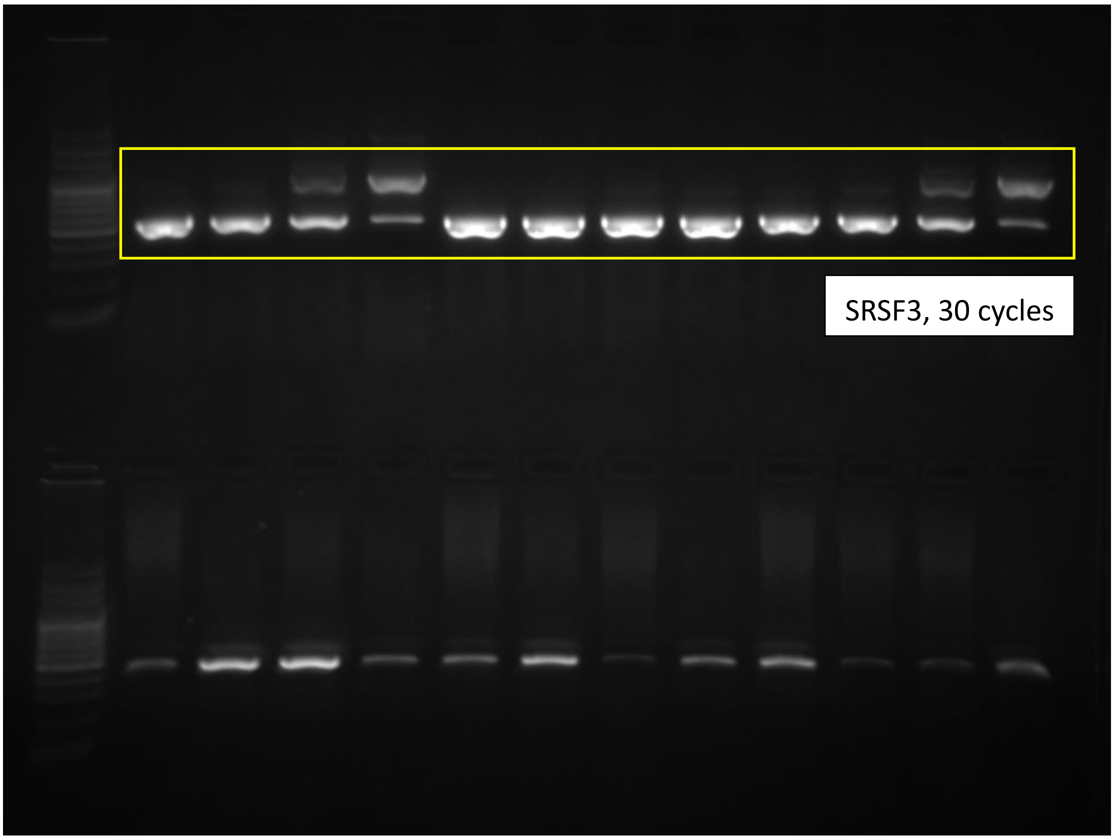
SRSF3, 30 cycles

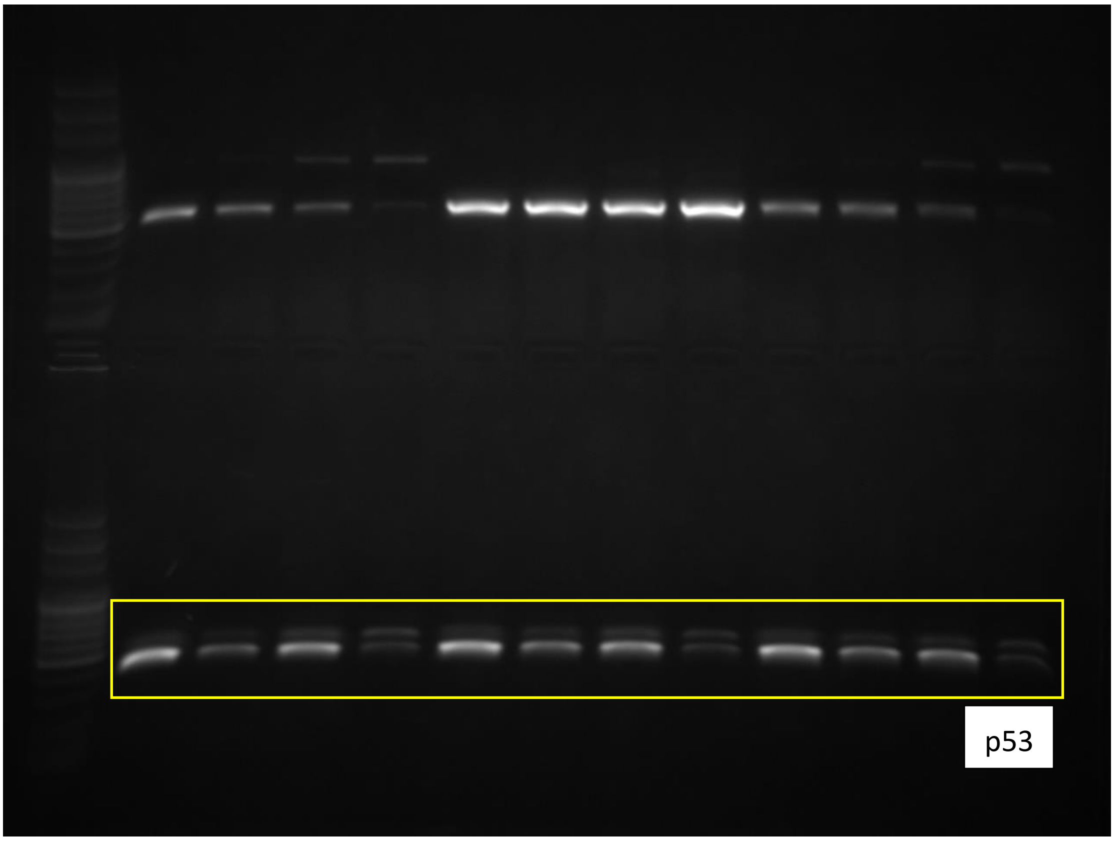
p53

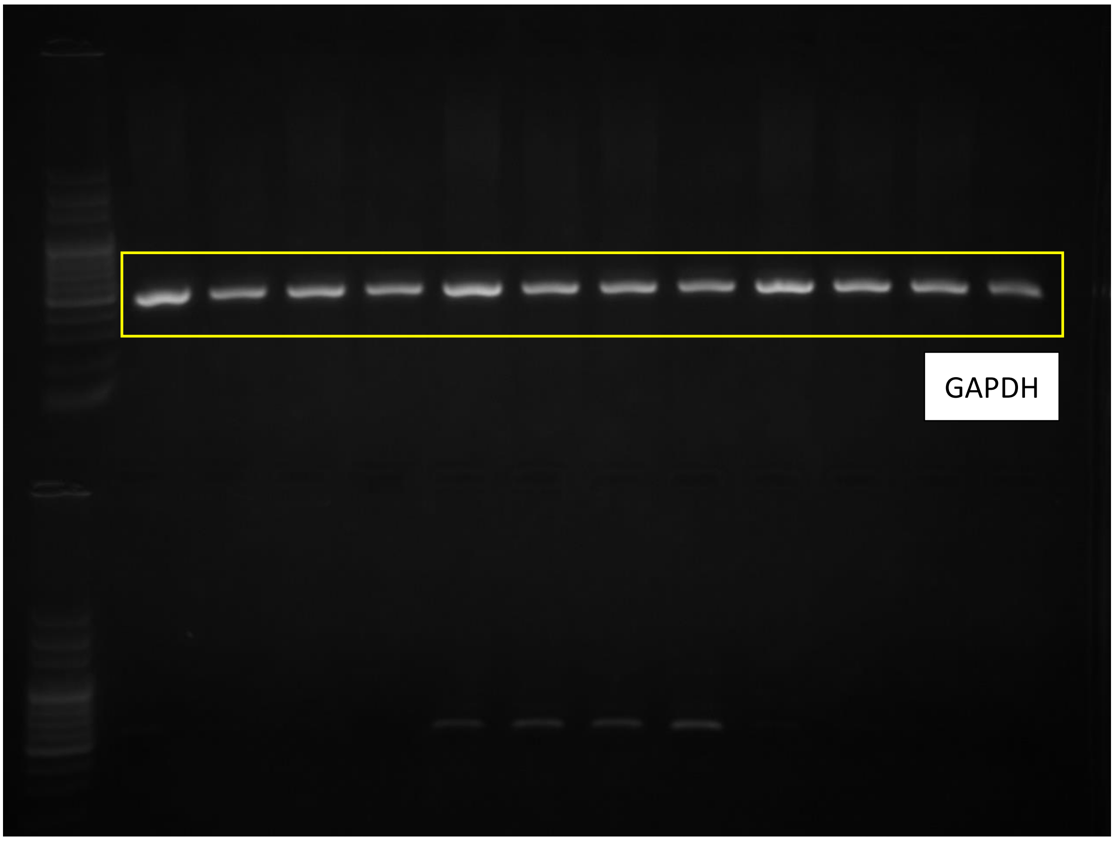
GAPDH

Figure 4A:

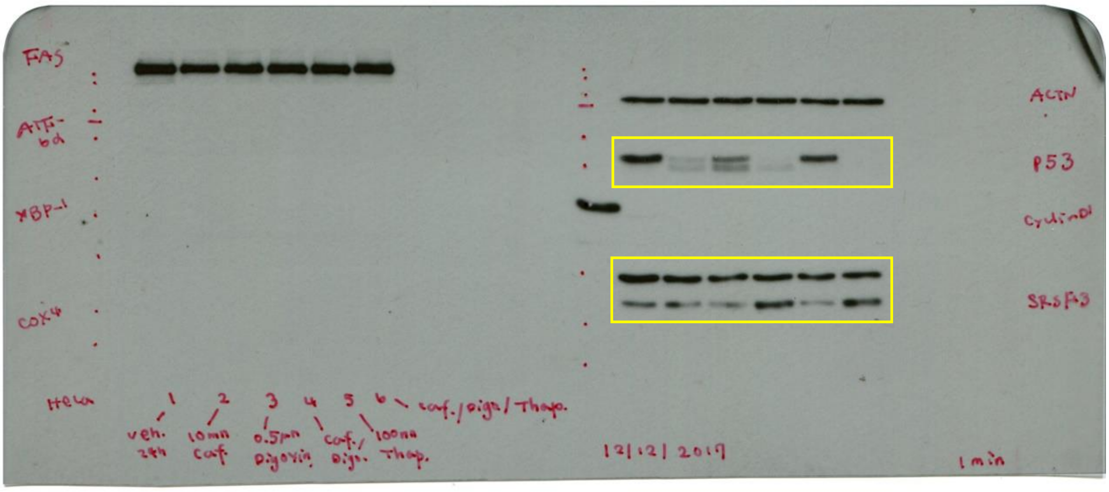
FAS
ACTN
P53
XBP-1
SRSF3
COX4
HeLa
1 2 3 4 5 6
12/12/2017
1 min

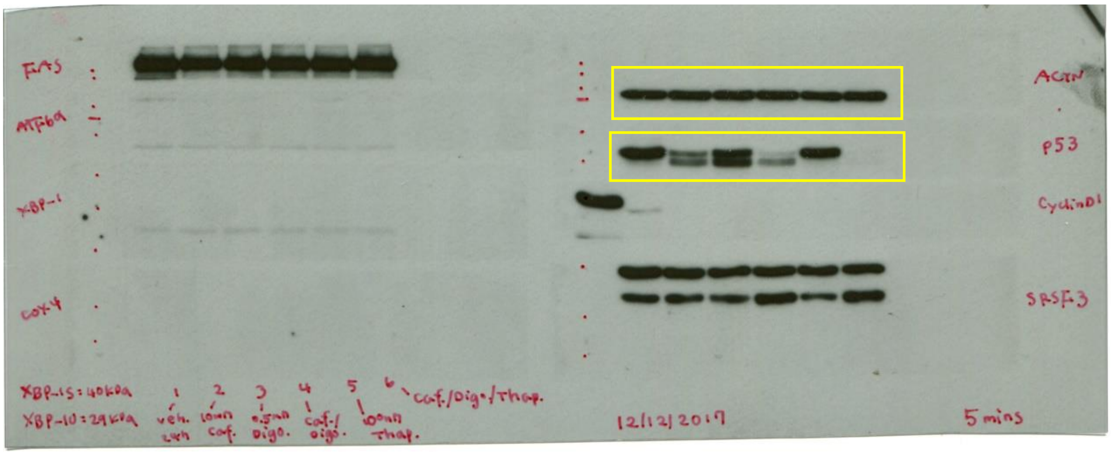
FAS
ATF6α
XBP-1
COX-4
ACTN
P53
CyclinD1
SRSF3
XBP-1S = 40kDa
XBP-1U = 29kDa
1 veh. 24h
2 10mM caf.
3 0.5nM Digo.
4 caf./digo.
5 100nM Thap.
6 caf./Digo./Thap.
12/12/2017
5 mins

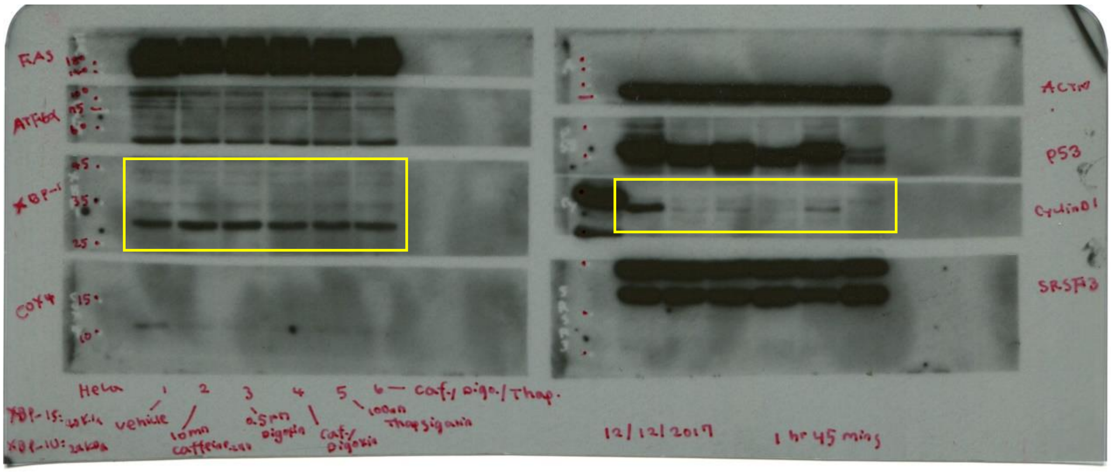
FAS
ATF6α
XBP-1
COX4
ACTN
P53
CyclinD1
SRSF3
Hela
1 vehicle
2 10mM caffeine
3 0.5nM digoxin
4 caf./Digoxin
5 100nM Thapsigargin
6 caf./digo./Thap.
12/12/2017
1 hr 45 mins

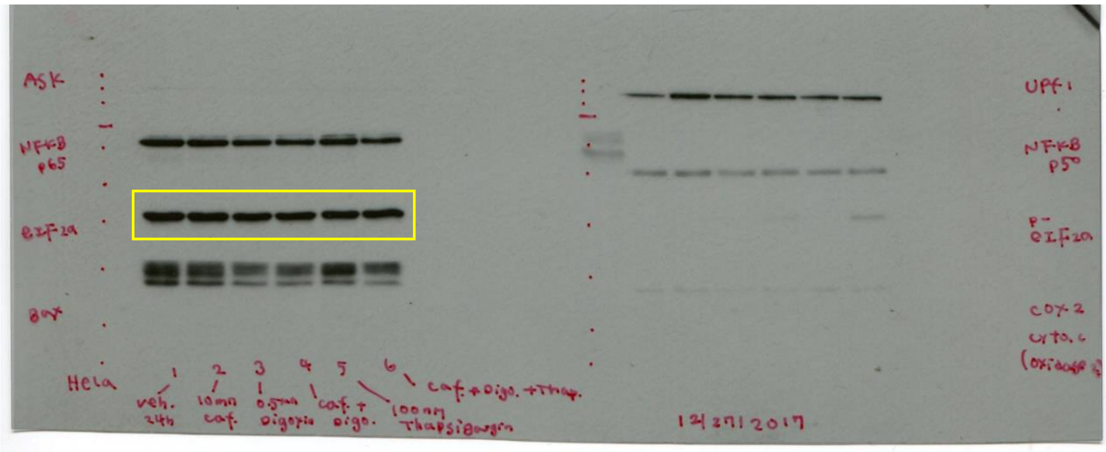
ASK
NFKB p65
eIF2α
Bax
UPF1
NFKB p50
p-eIF2α
COX-2
Cyto. c
Hela
1 veh. 24h
2 10mM caf.
3 0.5nM Digoxin
4 caf. + Digo.
5 100nM Thapsigargin
6 caf. + Digo. + Thap.
12/27/2017

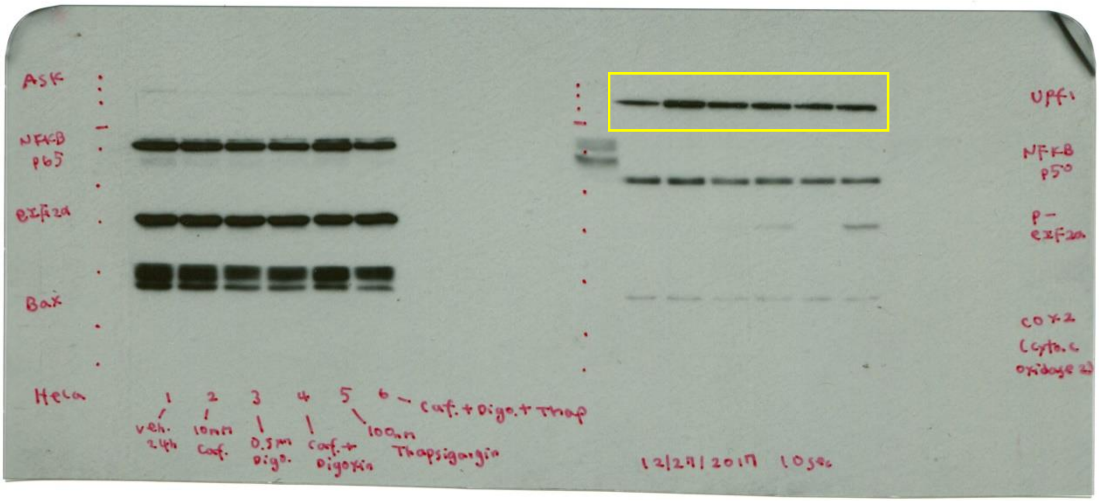
ASK
NFκB p65
eIF2α
Bax
Hela
Upf1
NFκB p50
p-eIF2α
cox-2 (cyto.c oxidase 2)
12/29/2017 10sec

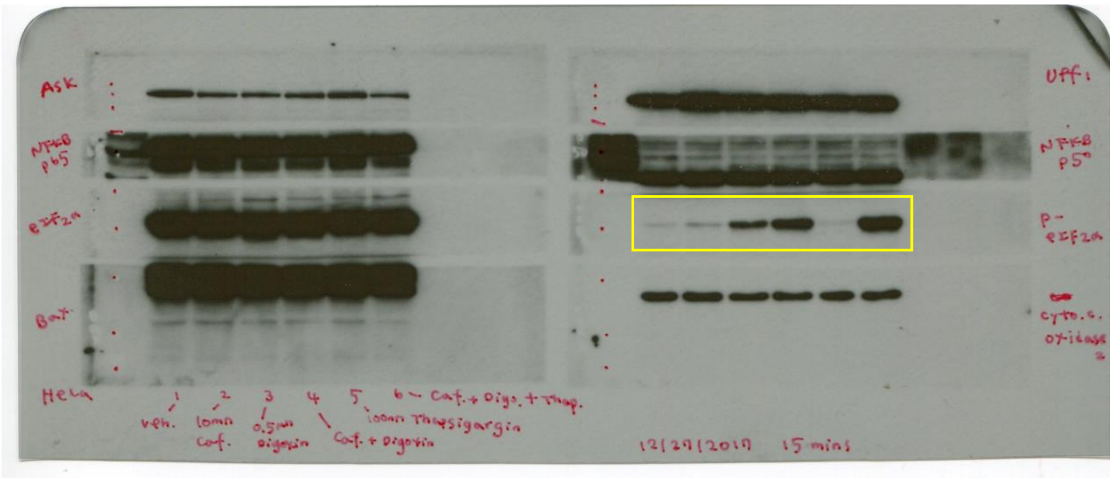
ASK
NFκB p65
eIF2α
Bax
Hela
Upf1
NFκB p50
p-eIF2α
cyto.c oxidase 2
12/29/2017 15 mins

Figure 4B:

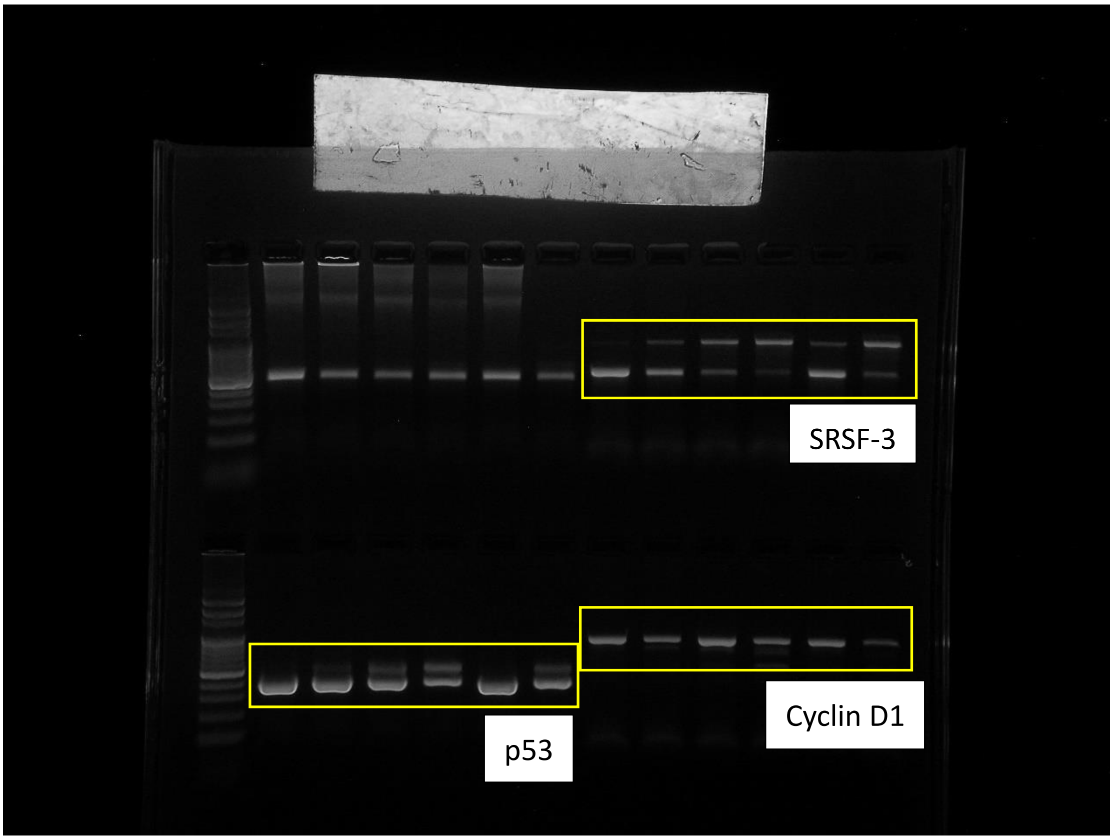

SRSF-3
Cyclin D1
p53

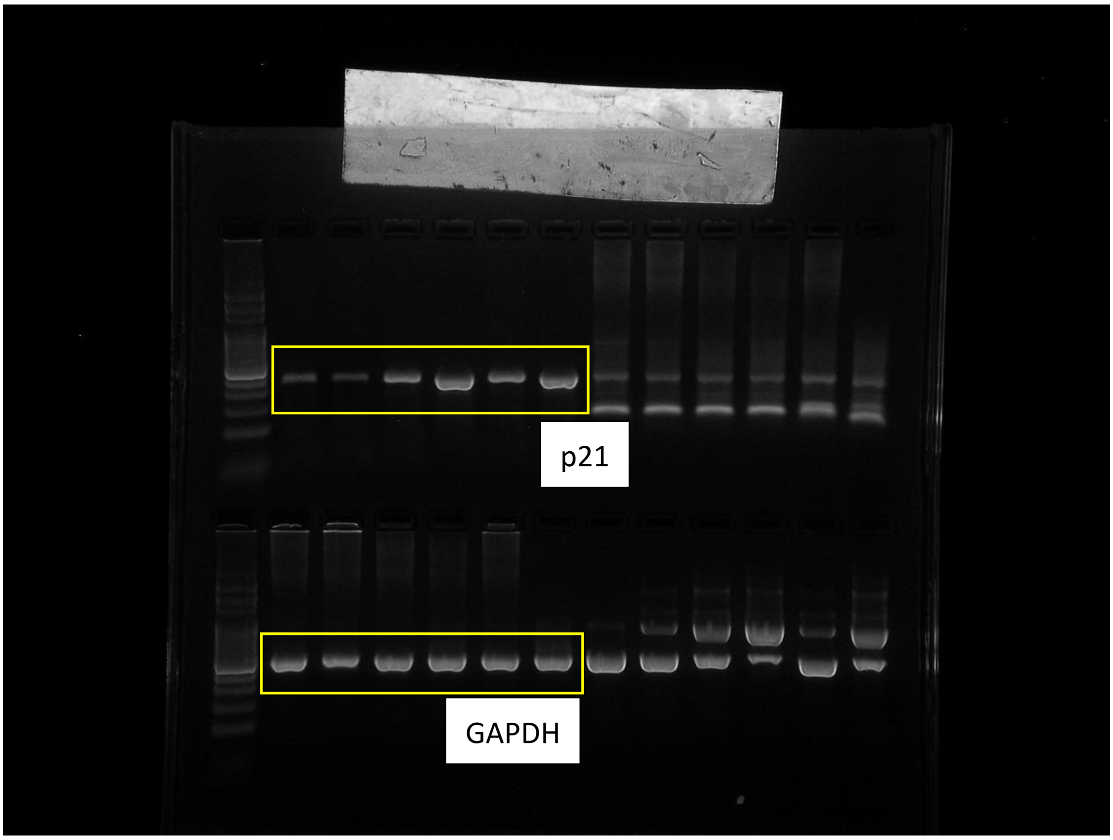

p21
GAPDH

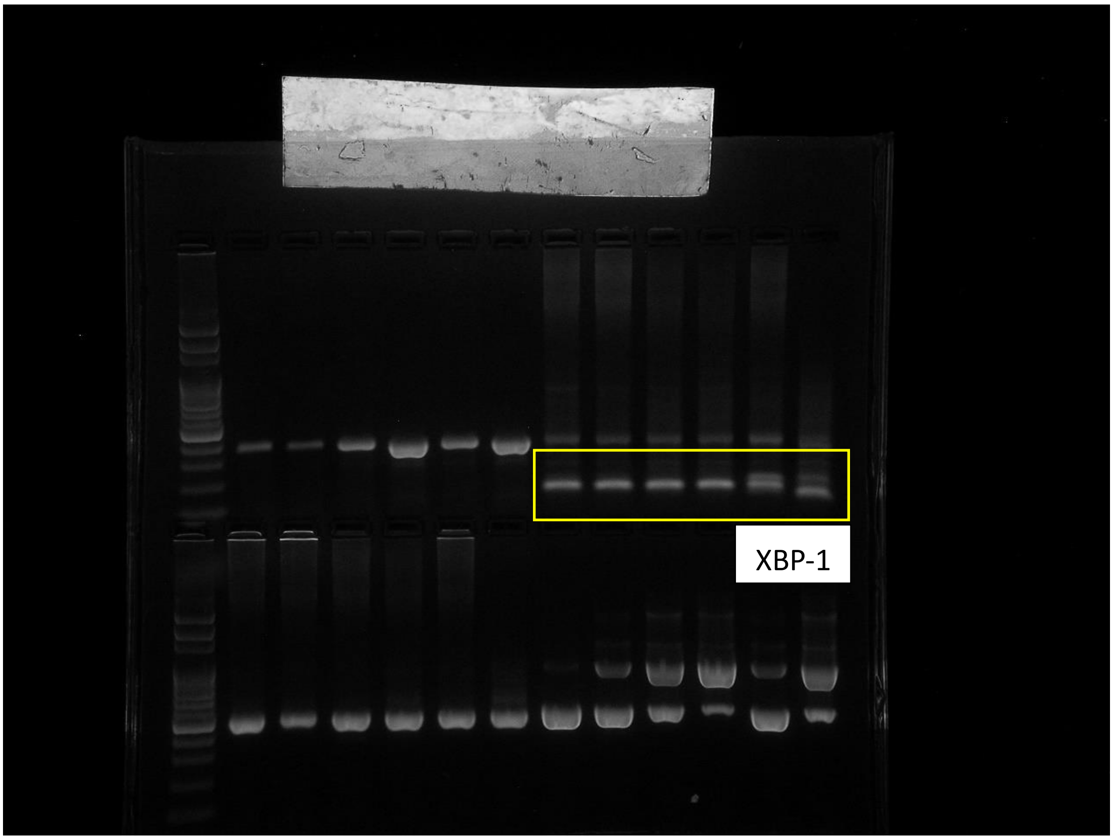

Figure 5B:

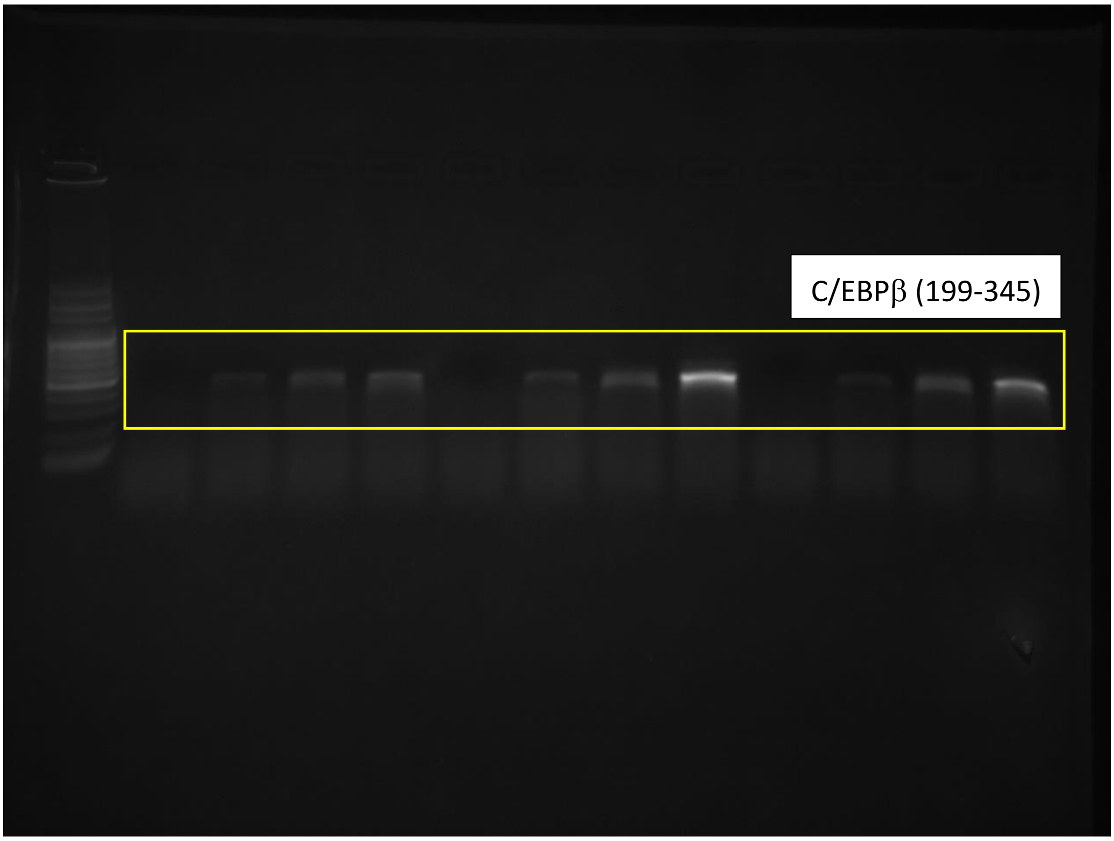

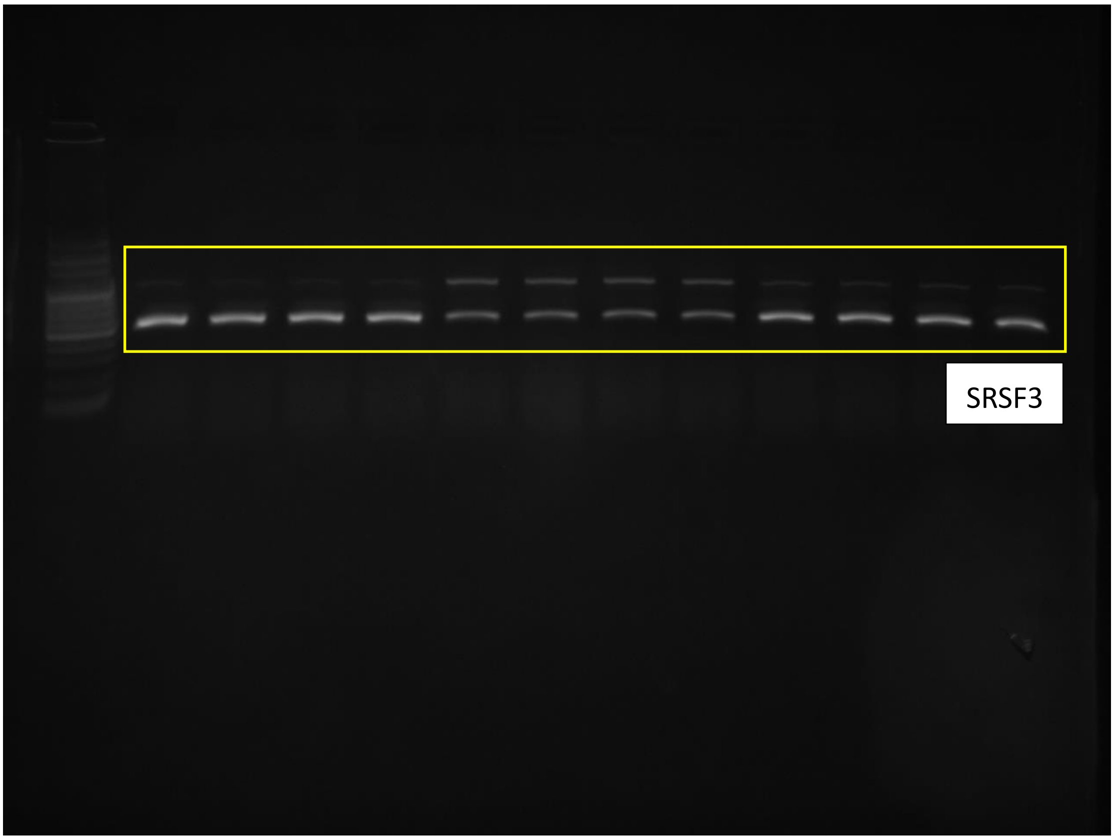
SRSF3

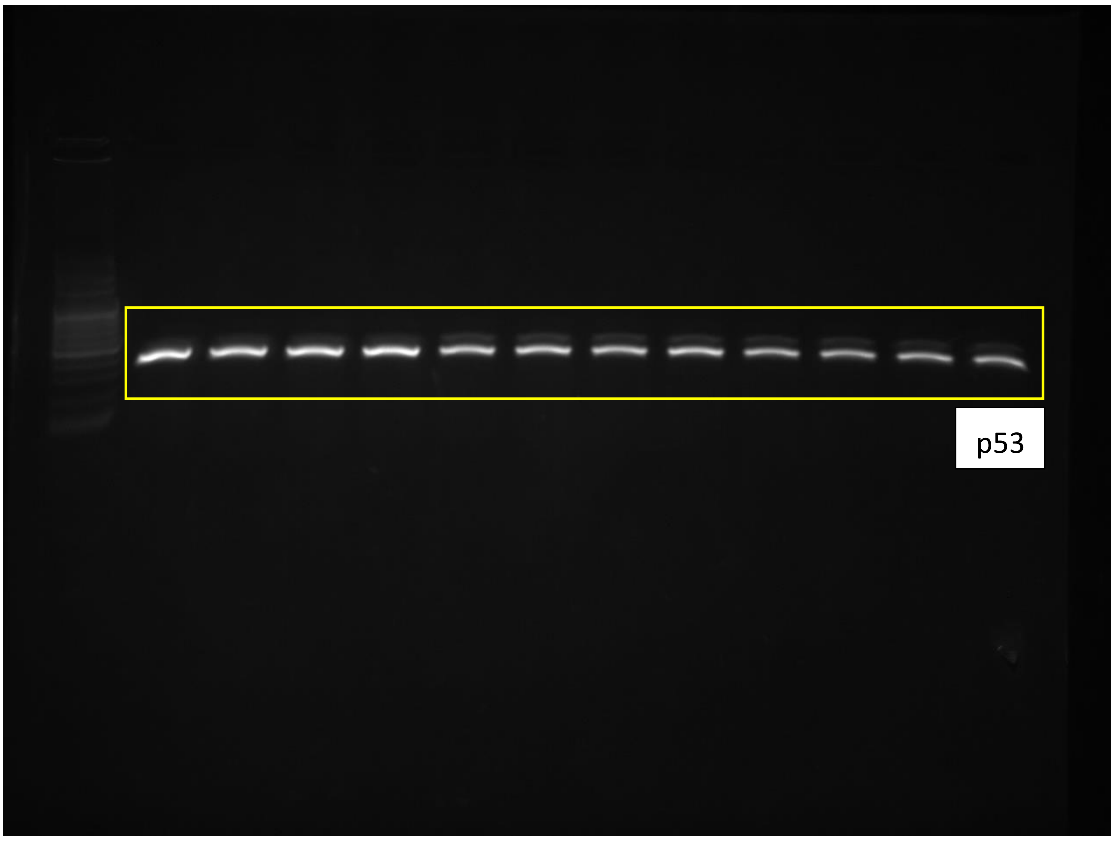
p53

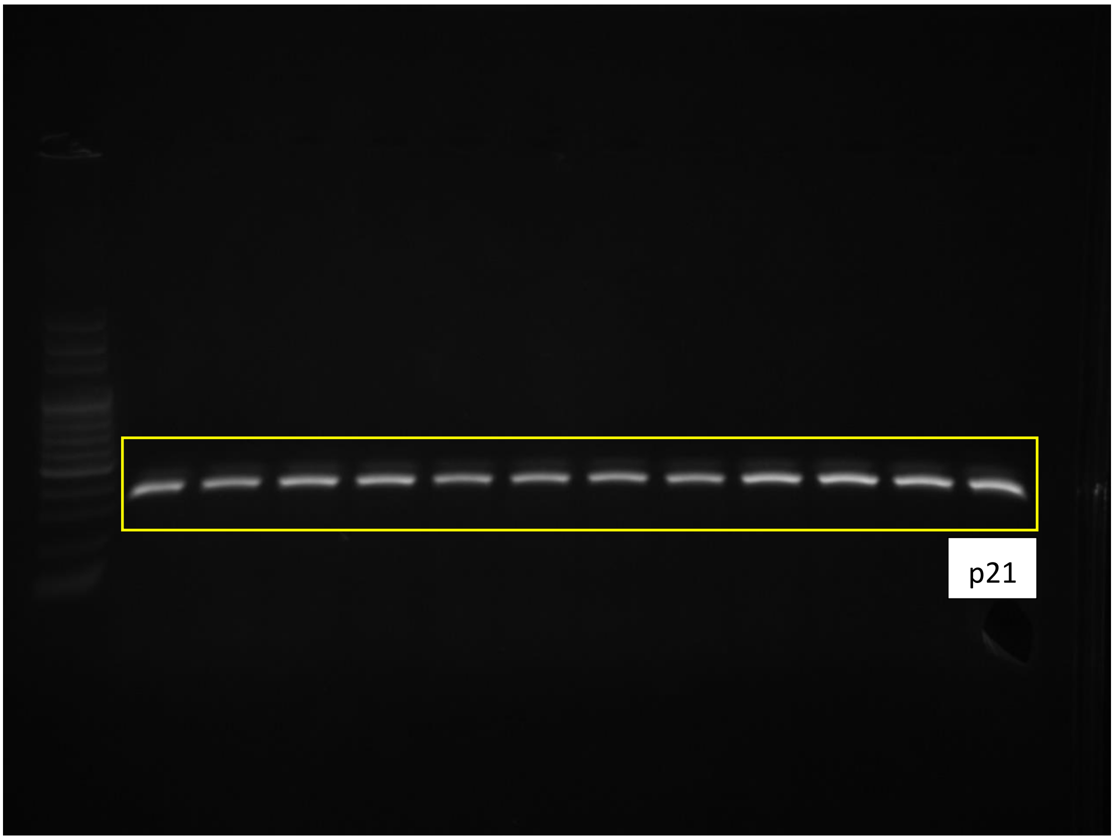
p21

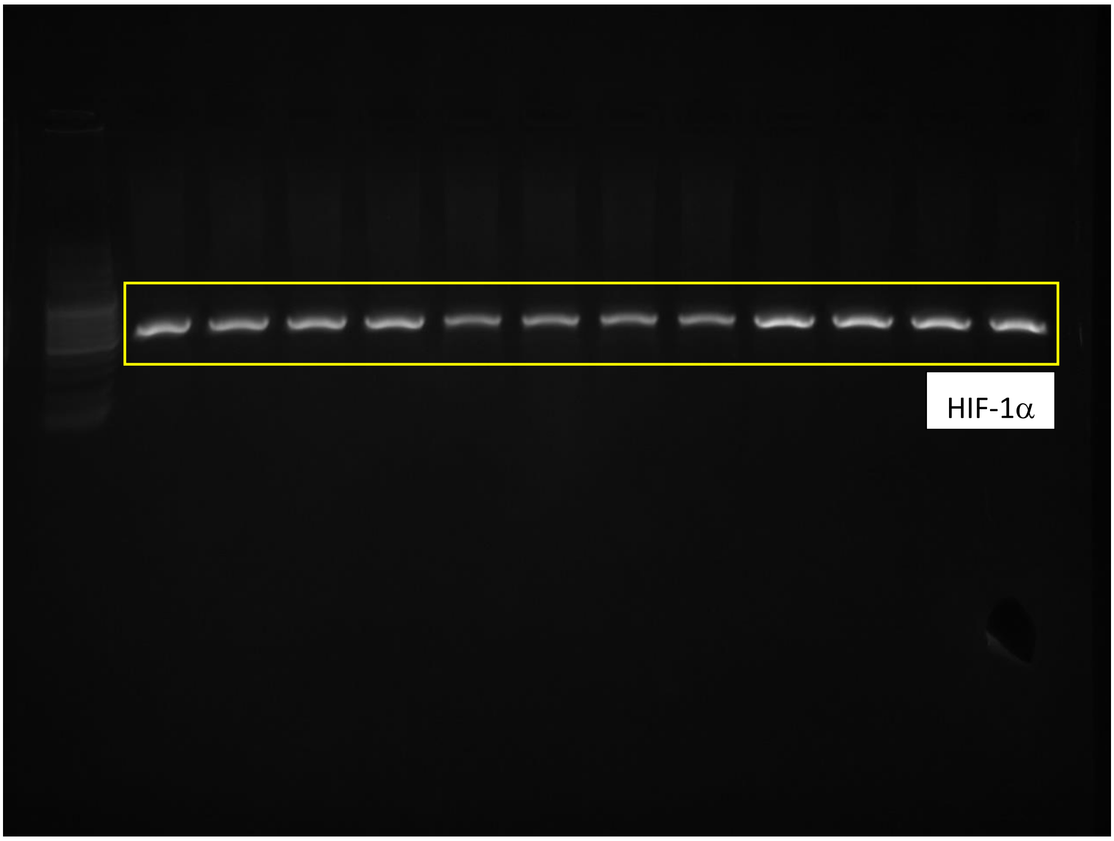
HIF-1α

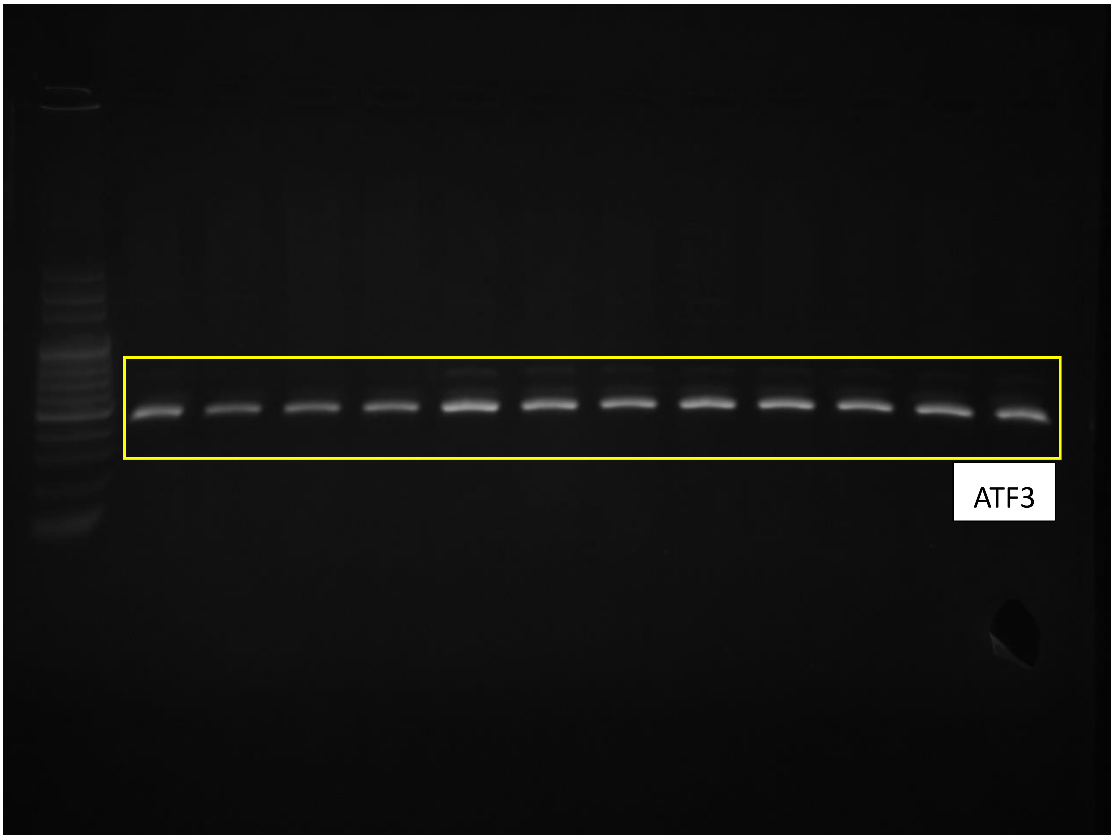

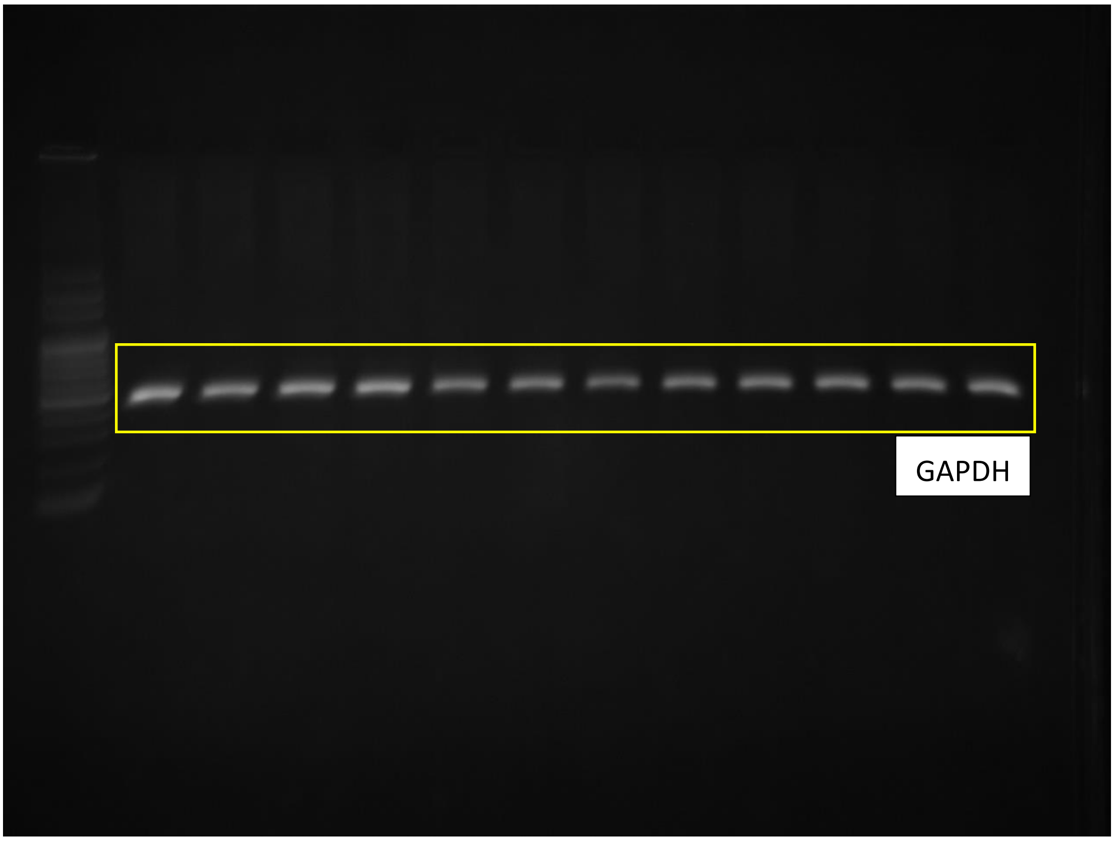

Figure 5C:

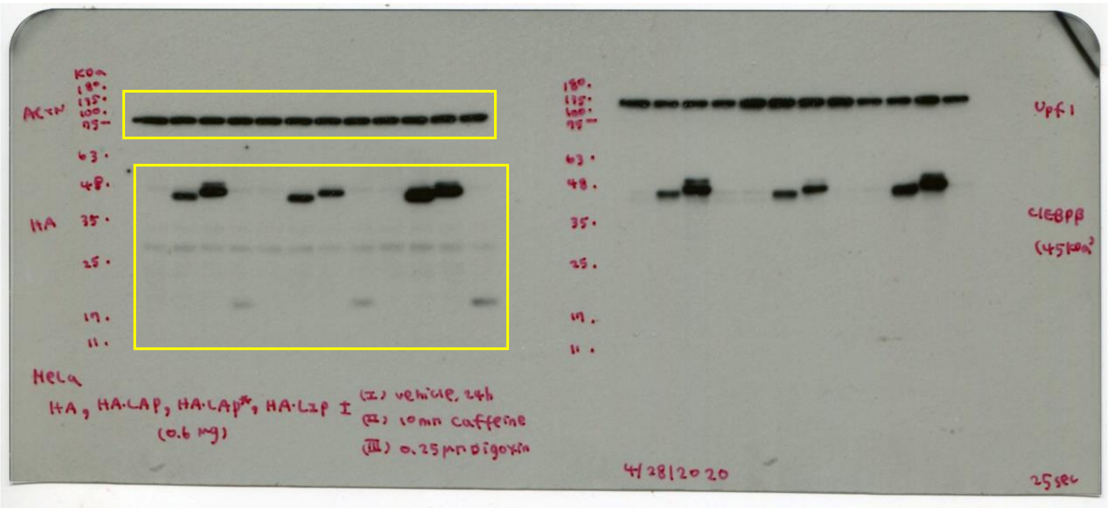
KDa
180
135
100
75
63
48
35
25
17
11
ACTN
HA
Upf1
C/EBPβ
(45kDa)
HeLa
HA, HA-LAP, HA-LAP*, HA-LIP ± (I) vehicle, 24h
(0.6 μg)
(II) 10mM Caffeine
(III) 0.25μM Digoxin
4/28/2020
25sec

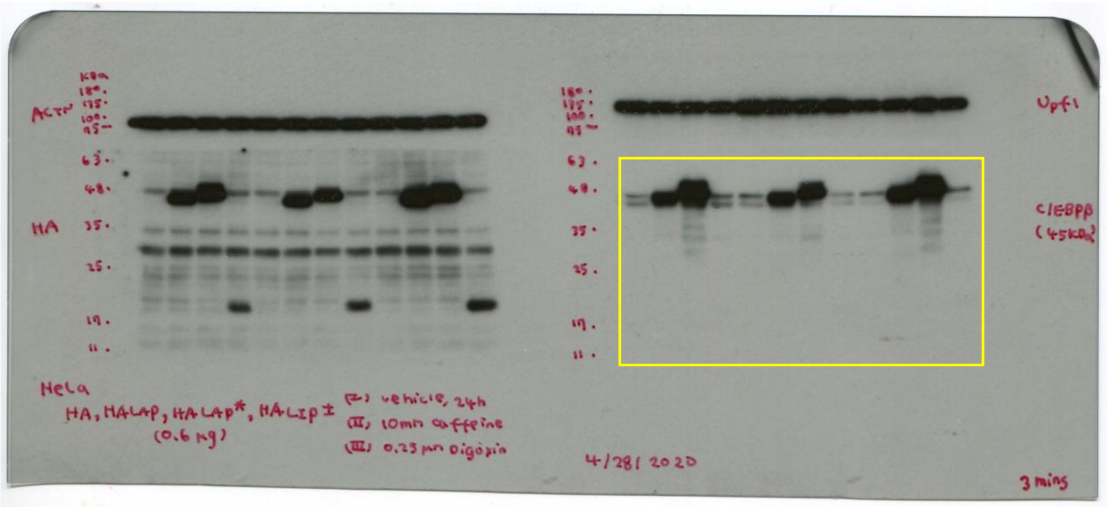
KDa
180
135
100
75
63
48
35
25
17
11
ACTN
HA
Upf1
C/EBPβ
(45kDa)
HeLa
HA, HALAP, HA-LAP*, HA-LIP ± (I) vehicle, 24h
(0.6 μg)
(II) 10mM Caffeine
(III) 0.25μM Digoxin
4/28/2020
3 mins

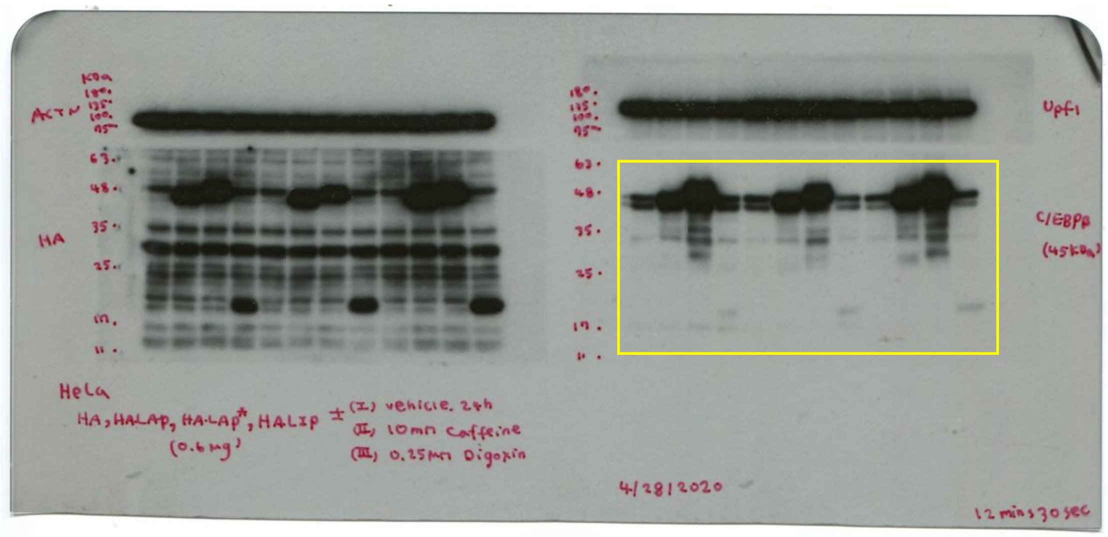
KDa
180
135
100
75
63
48
35
25
17
11
ACTN
HA
Upf1
C/EBPβ
(45kDa)
HeLa
HA, HALAP, HA-LAP*, HALIP ± (I) vehicle, 24h
(0.6 μg)
(II) 10mM Caffeine
(III) 0.25μM Digoxin
4/28/2020
12 mins 30 sec

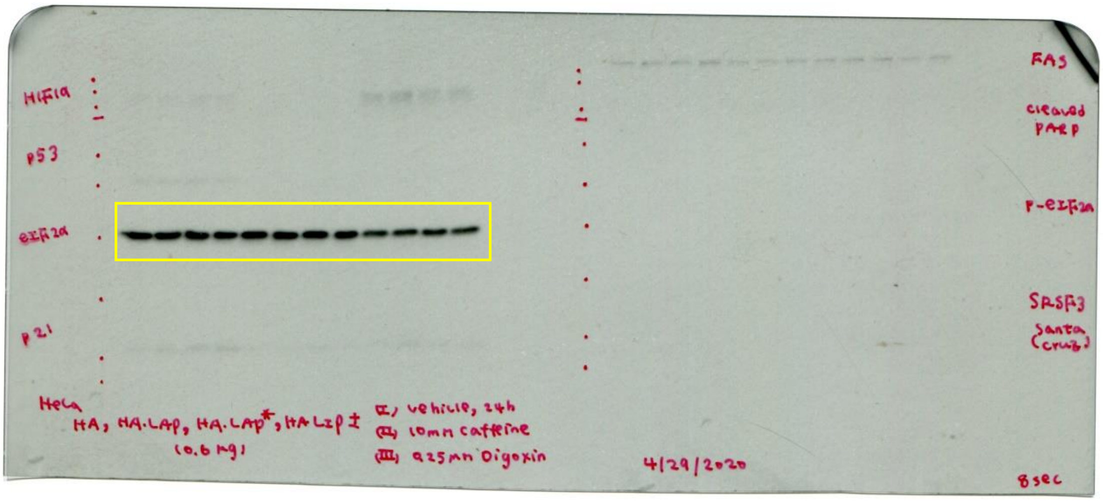
HIF1α
p53
eIF2α
p21
HeLa
HA, HA-LAP, HA-LAP*, HA-LIP ±
(0.6 μg)
(I) vehicle, 24h
(II) 10mM caffeine
(III) 0.25mM Digoxin
4/29/2020
FAS
cleaved PARP
p-eIF2α
SRSF3 (Santa Cruz)
8sec

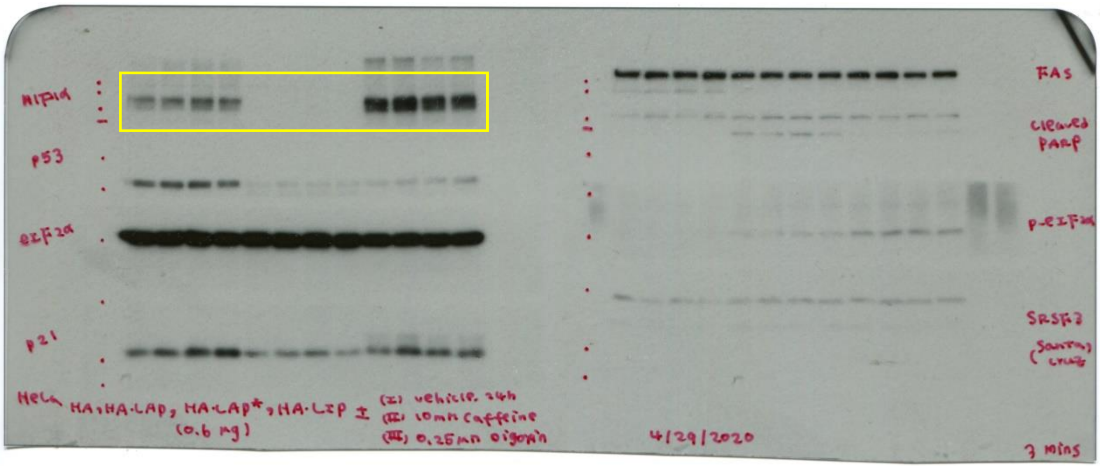
HIF1α
p53
eIF2α
p21
HeLa
HA, HA-LAP, HA-LAP*, HA-LIP ±
(0.6 μg)
(I) vehicle, 24h
(II) 10mM caffeine
(III) 0.25mM Digoxin
4/29/2020
FAS
cleaved PARP
p-eIF2α
SRSF3 (Santa Cruz)
3 mins

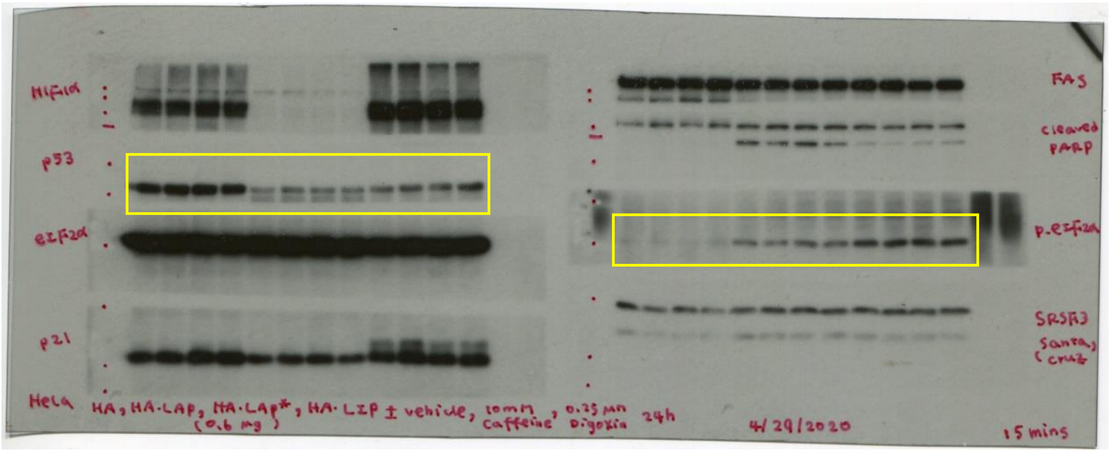
HIF1α
p53
eIF2α
p21
HeLa
HA, HA-LAP, HA-LAP*, HA-LIP ± vehicle, 10mM caffeine, 0.25mM Digoxin 24h
(0.6 μg)
4/29/2020
FAS
cleaved PARP
p-eIF2α
SRSF3 (Santa Cruz)
15 mins

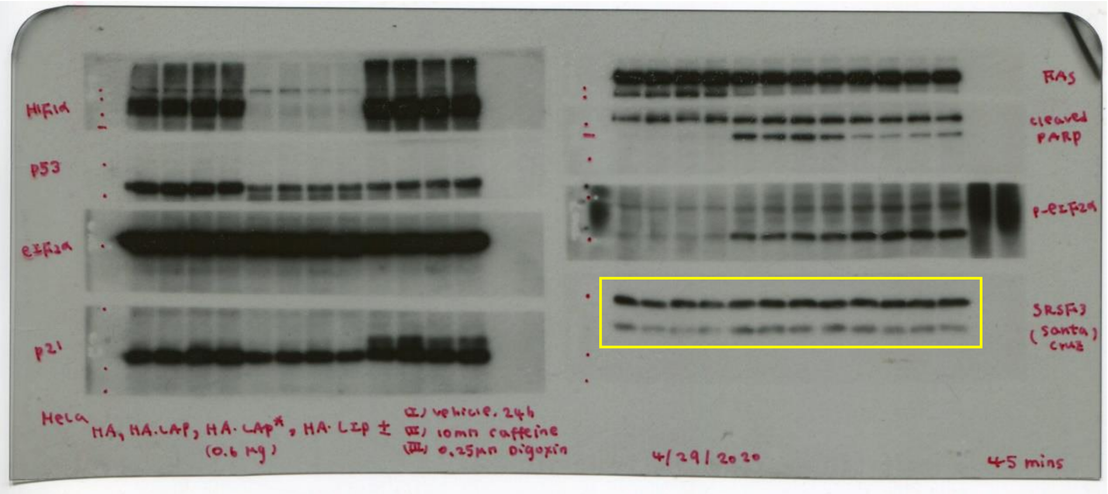

Figure 6A:

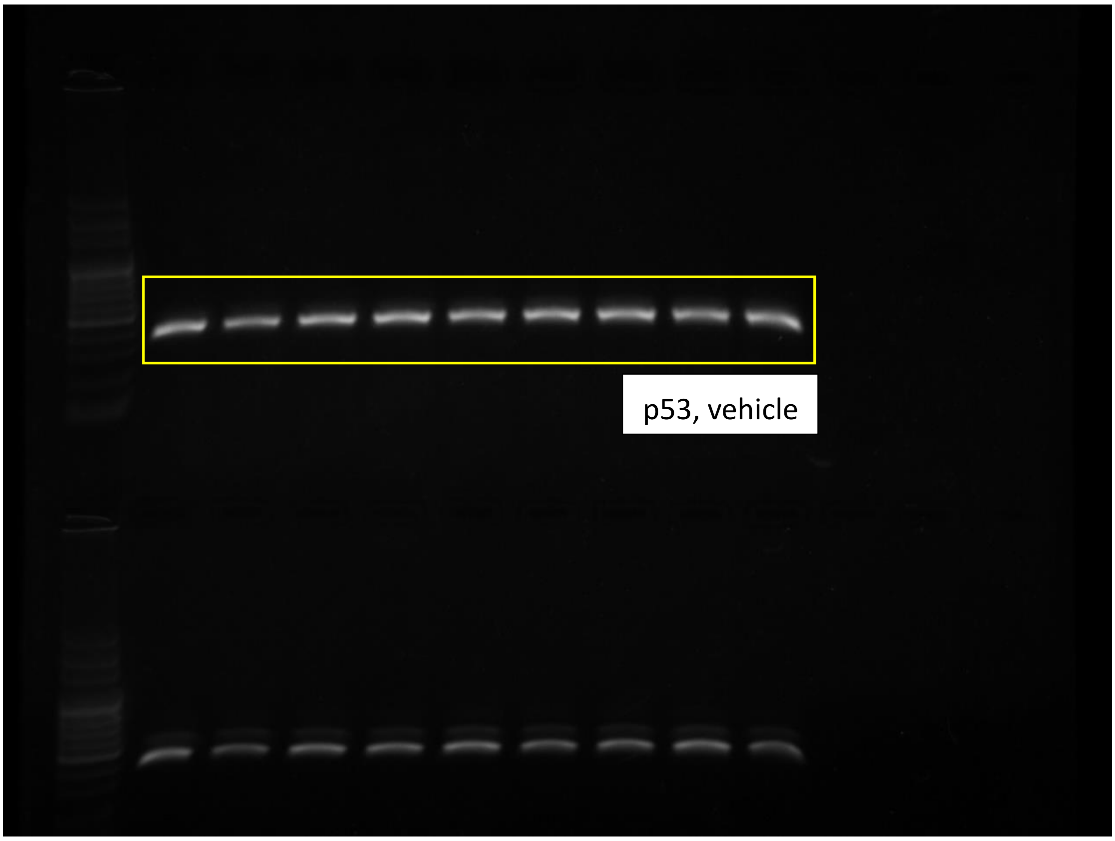

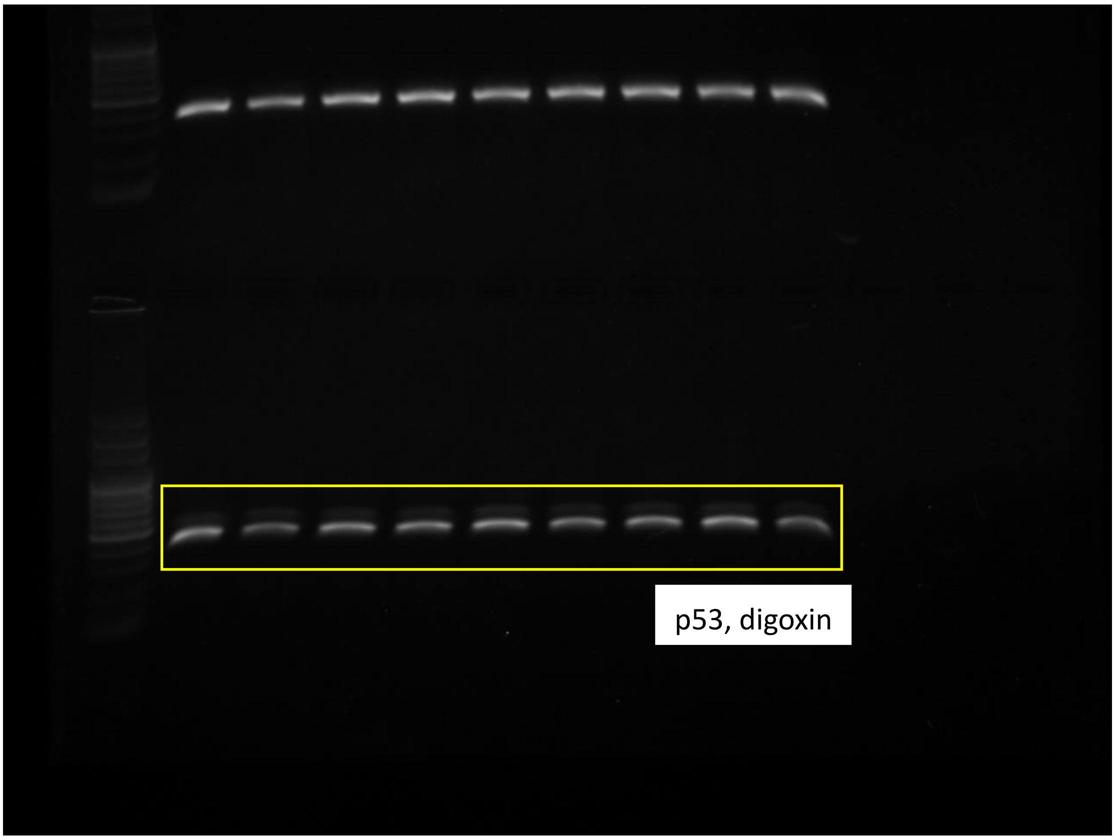
p53, digoxin

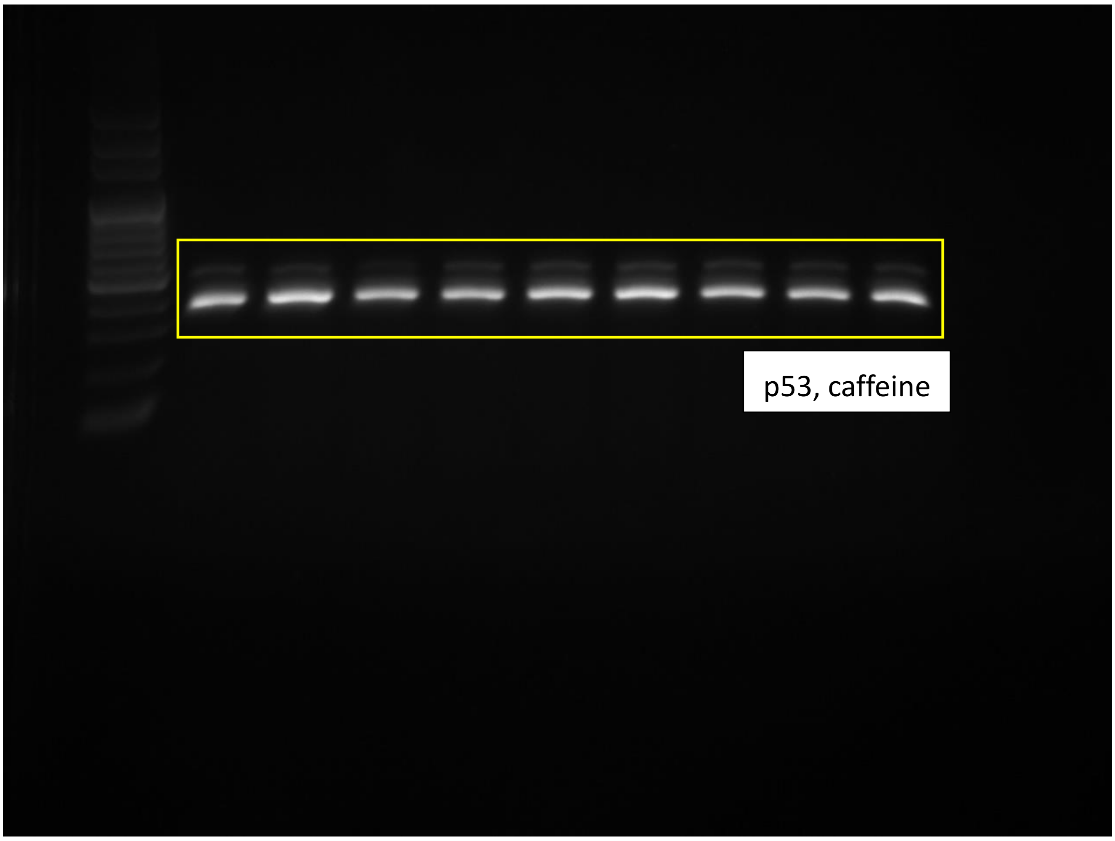
p53, caffeine

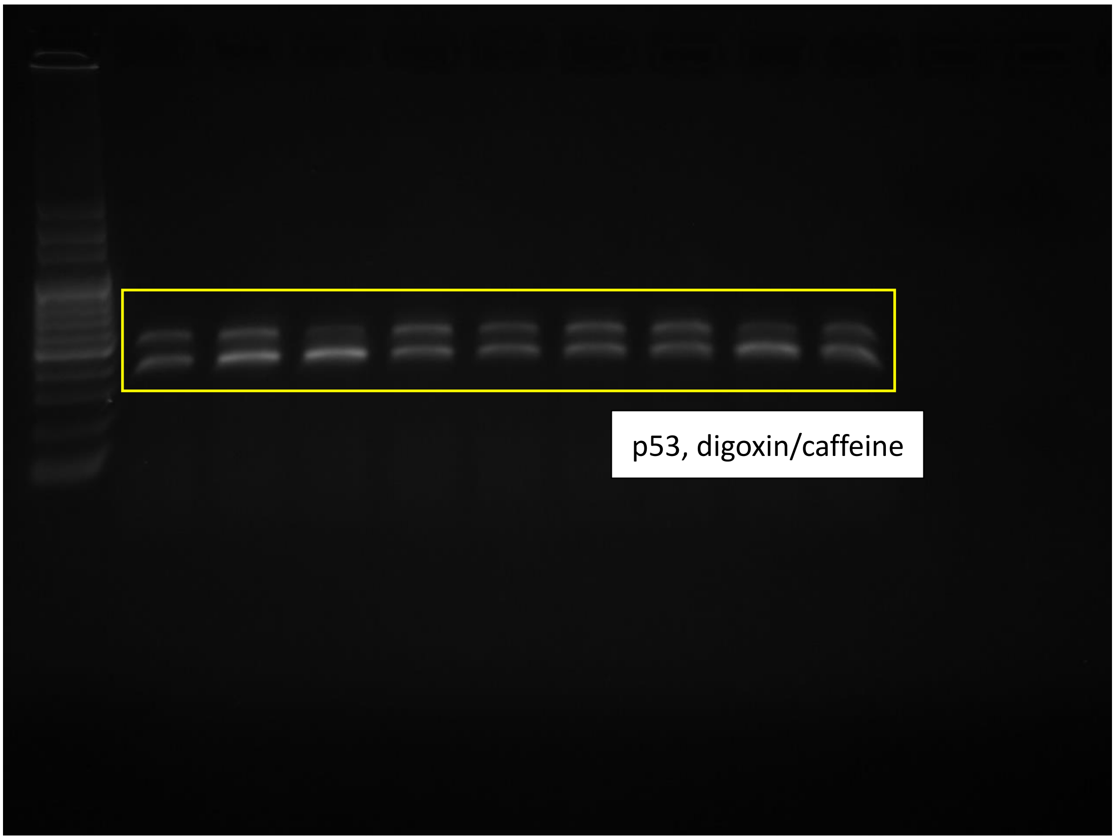
p53, digoxin/caffeine

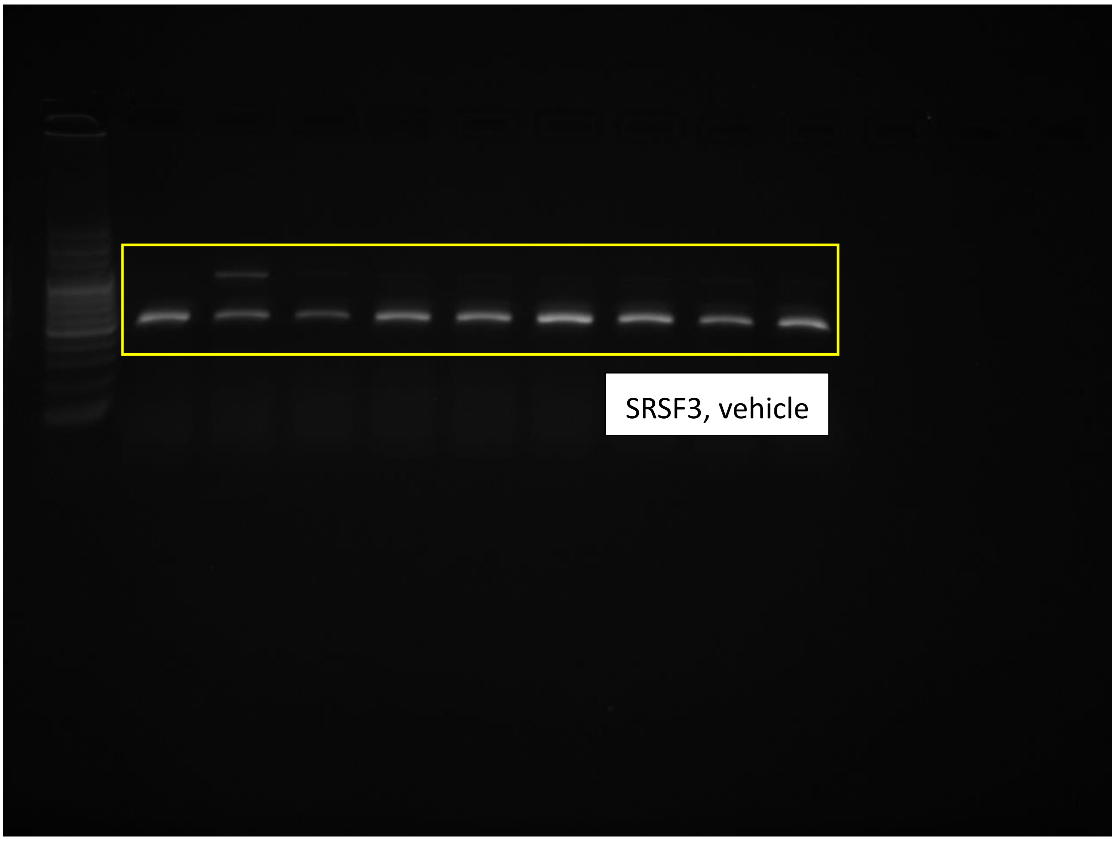
SRSF3, vehicle

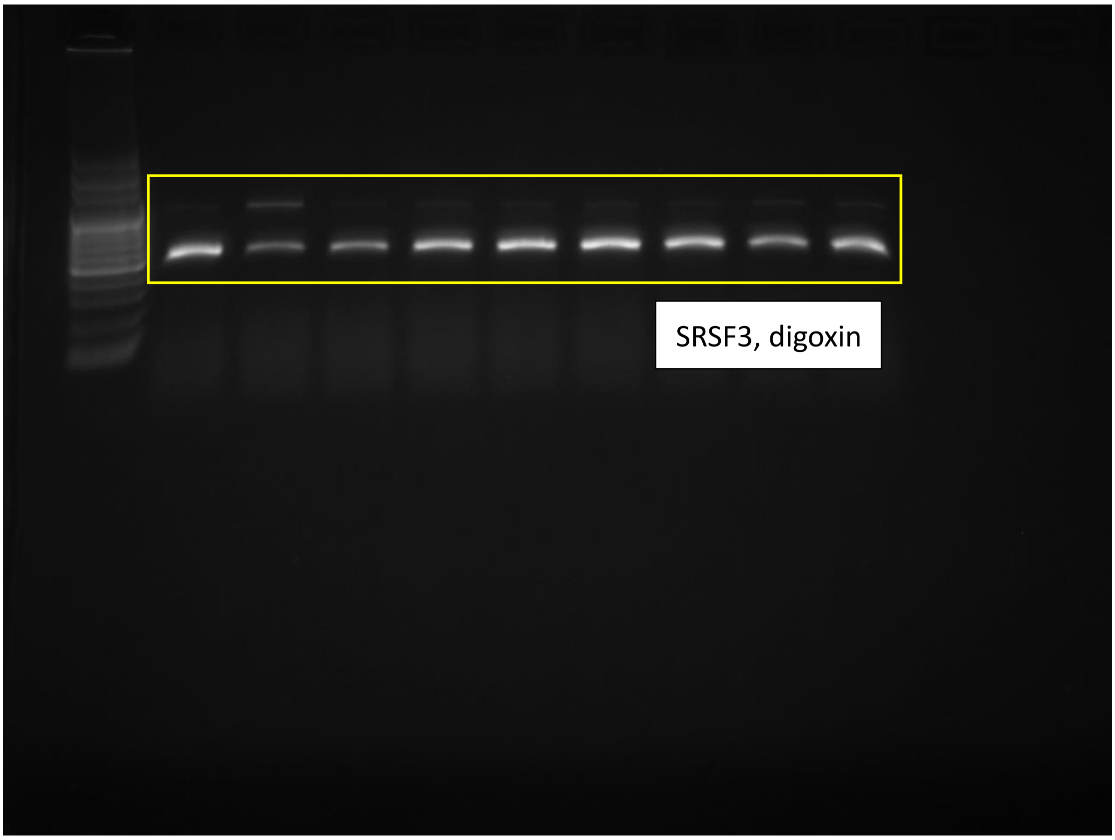
SRSF3, digoxin

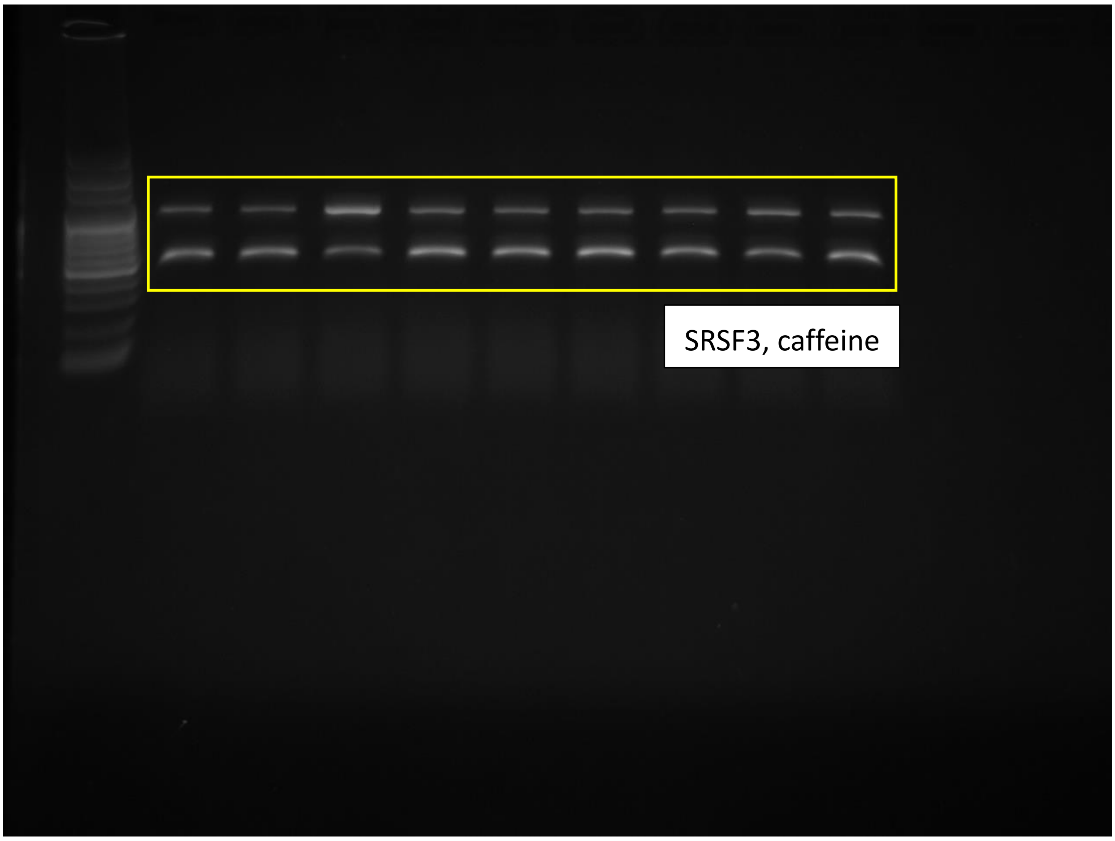
SRSF3, caffeine

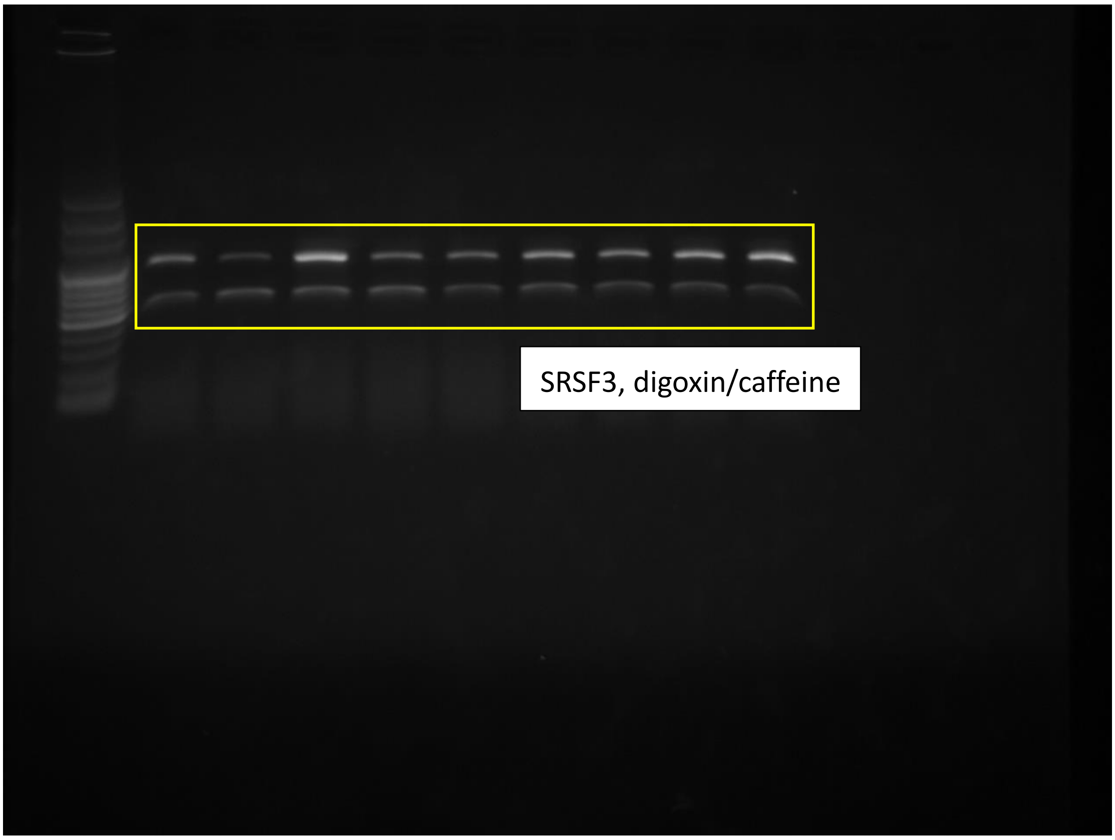
SRSF3, digoxin/caffeine

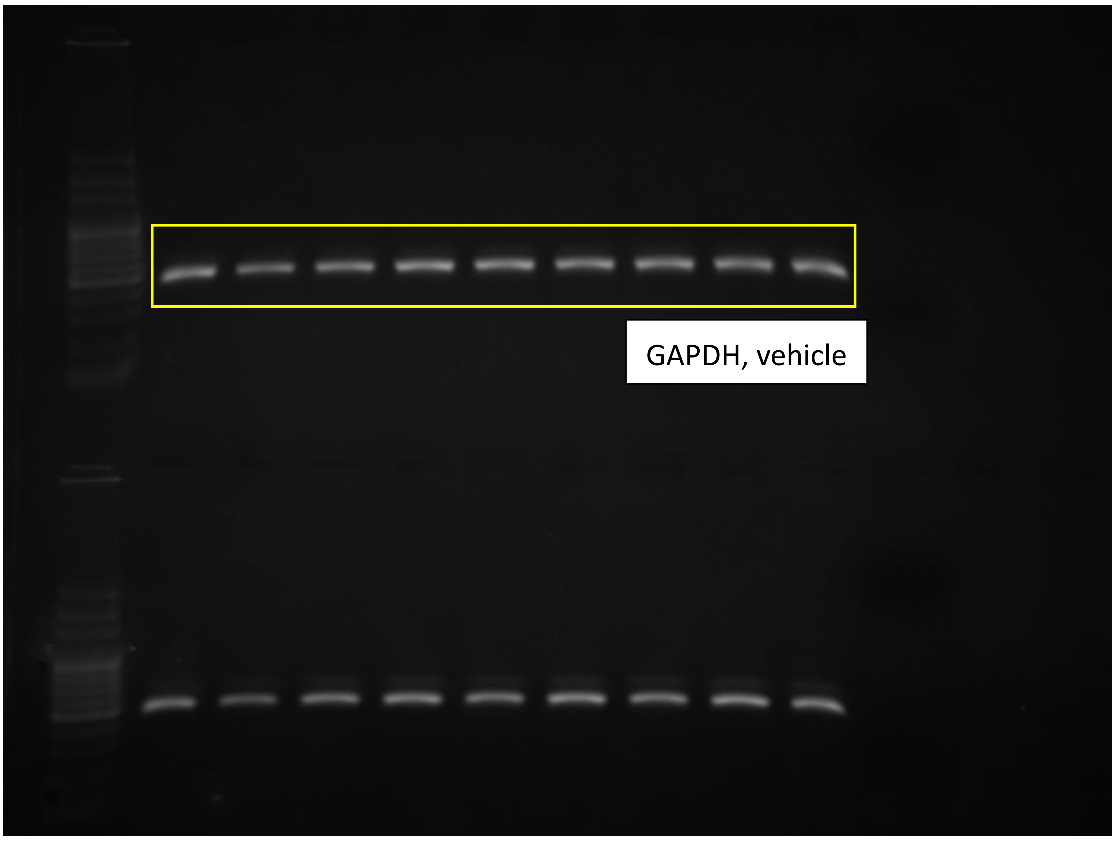
GAPDH, vehicle

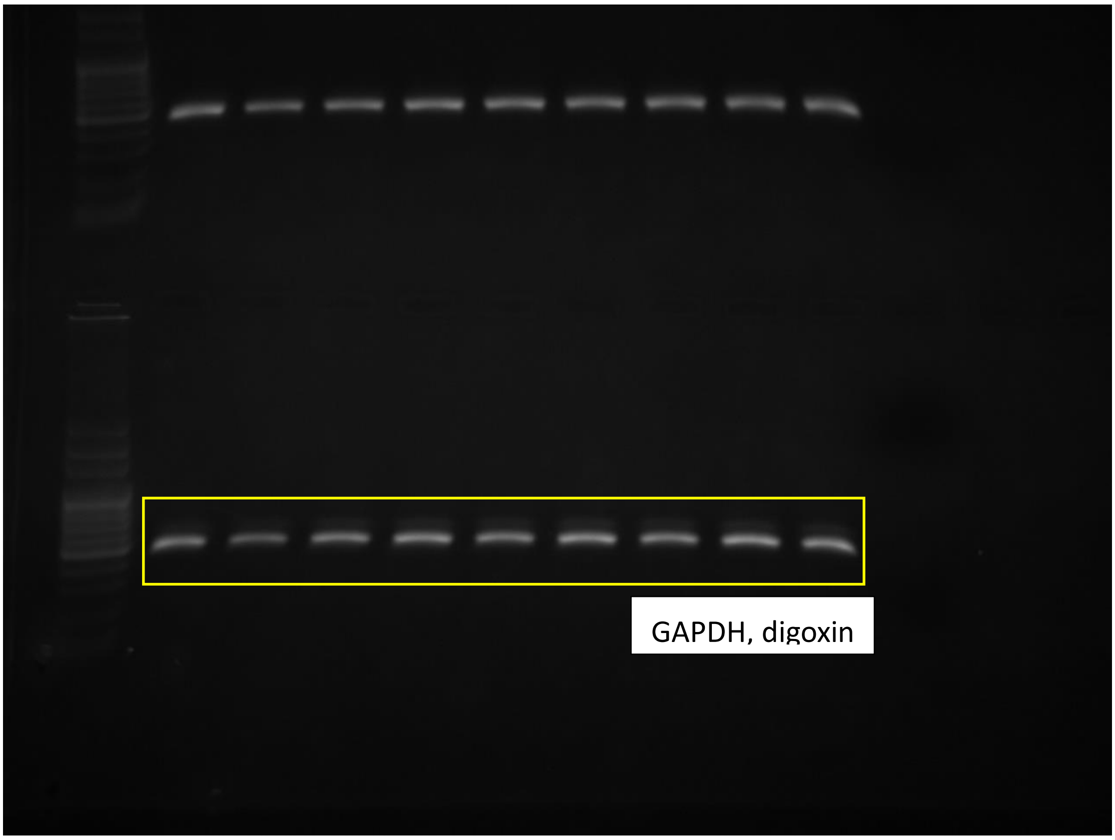
GAPDH, digoxin

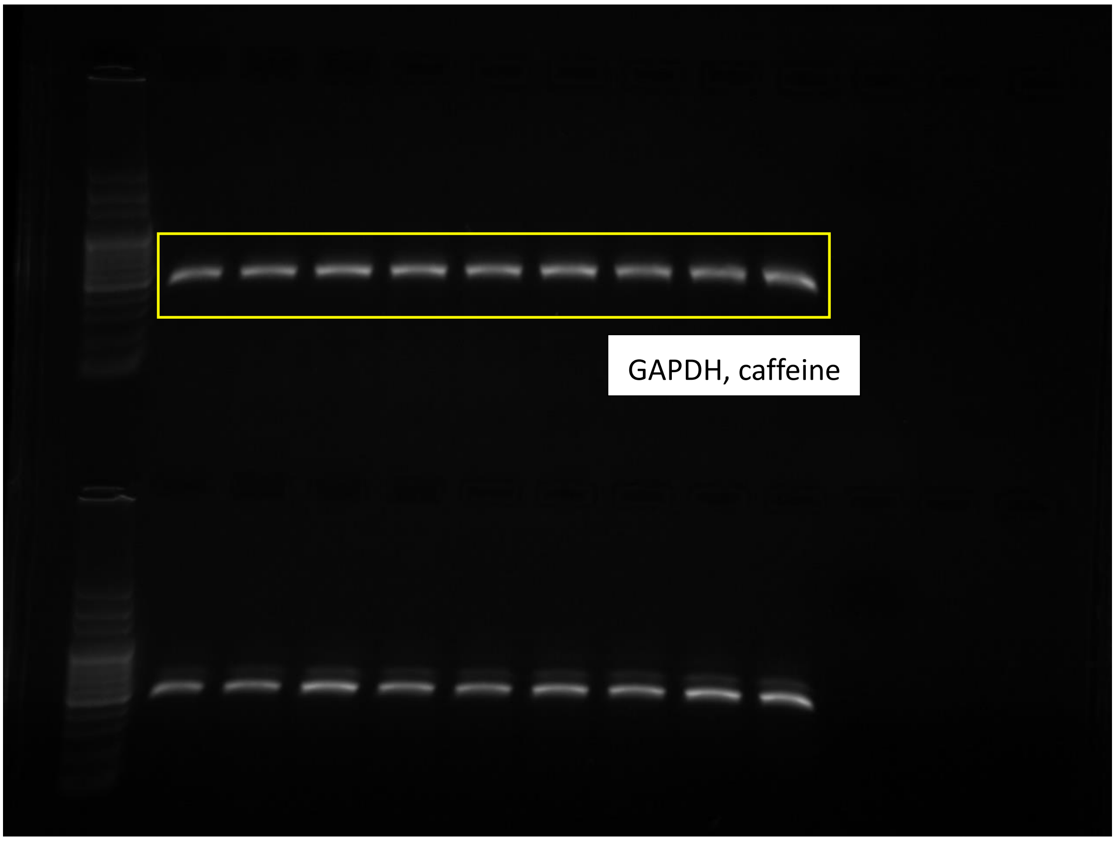
GAPDH, caffeine

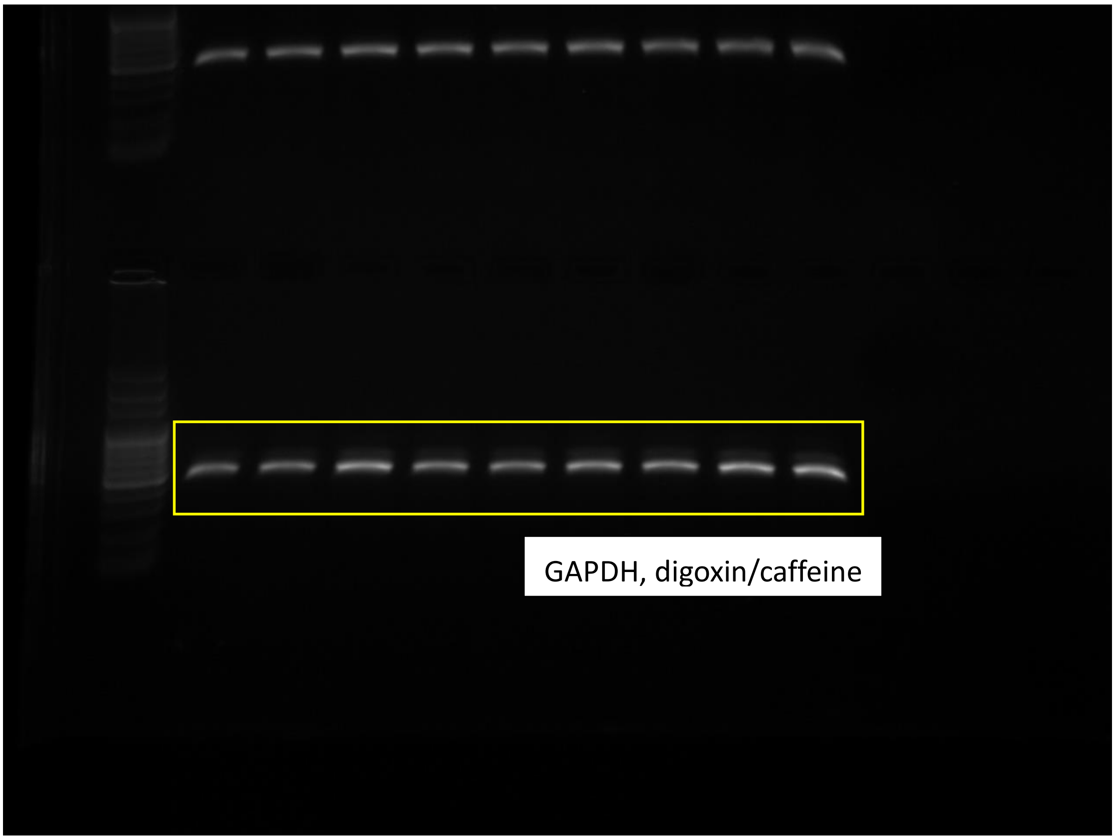
GAPDH, digoxin/caffeine

Figure 6B:

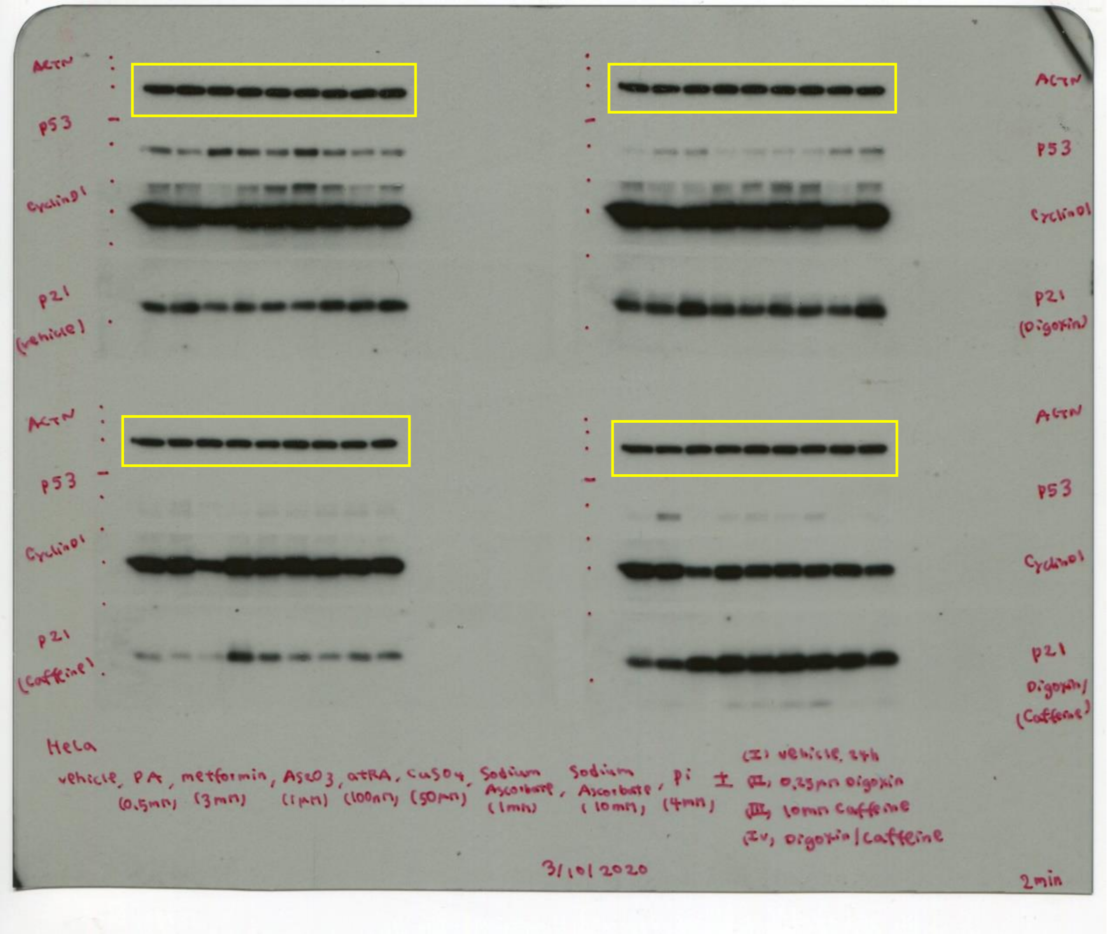
ACTN
P53
CyclinD1
P21
(vehicle)
P21
(Digoxin)
(Caffeine)
HeLa
vehicle, PA, metformin, As2O3, atRA, CuSO4, Sodium Ascorbate, Sodium Ascorbate, Pi
(0.5mM) (3mM) (1μM) (100nM) (50μM) (1mM) (10mM) (4mM)
(I) vehicle, 24h
(II) 0.25μM digoxin
(III) 10mM caffeine
(IV) digoxin/caffeine
3/10/2020
2min

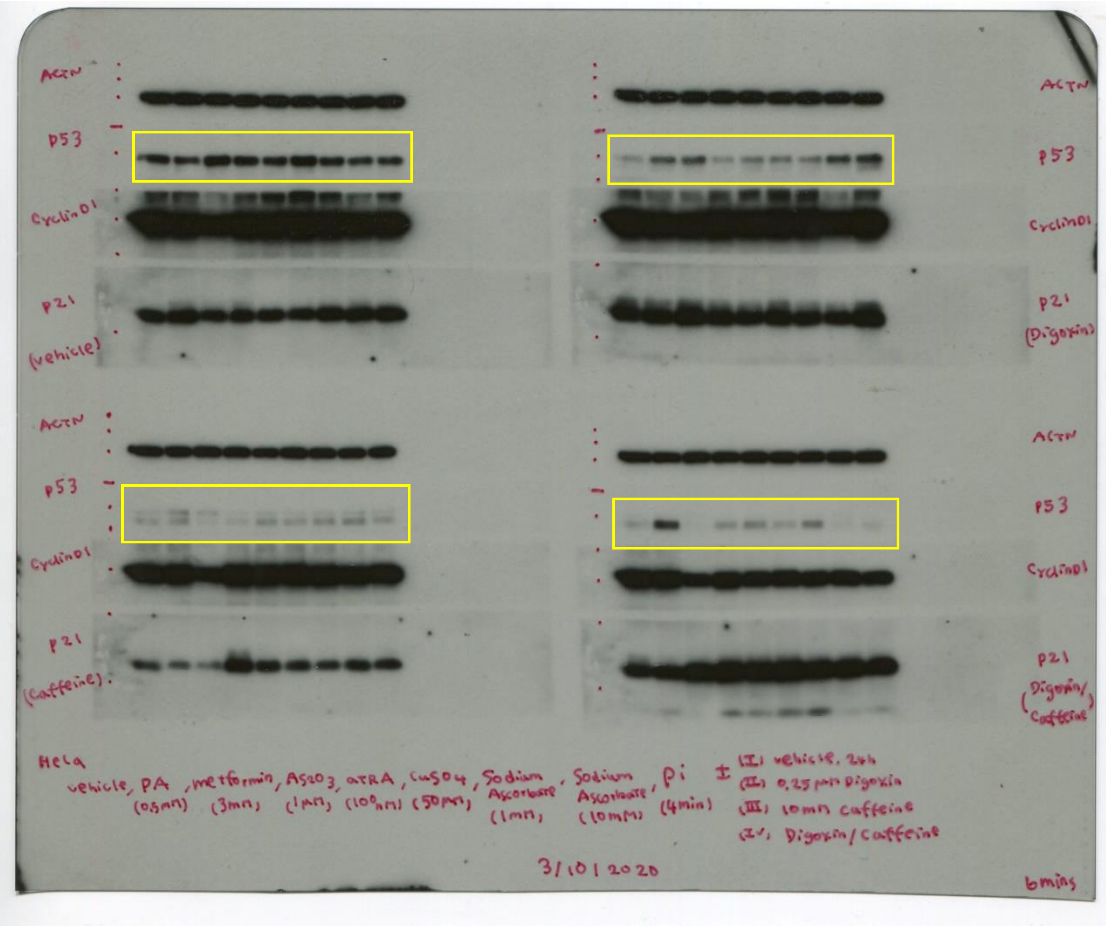
ACTN
p53
CyclinD1
p21
(vehicle)
ACTN
p53
CyclinD1
p21
(caffeine)
ACTN
p53
CyclinD1
p21
(Digoxin)
ACTN
p53
CyclinD1
p21
(Digoxin/Caffeine)
Hela
vehicle, PA (0.5mM), metformin (3mM), As2O3 (1μM), aTRA (100nM), CuSO4 (50μM), Sodium Ascorbate (1mM), Sodium Ascorbate (10mM), Pi (4mM)
I vehicle, 24h
II 0.25 μM Digoxin
III 10mM caffeine
IV Digoxin/Caffeine
3/10/2020
6mins

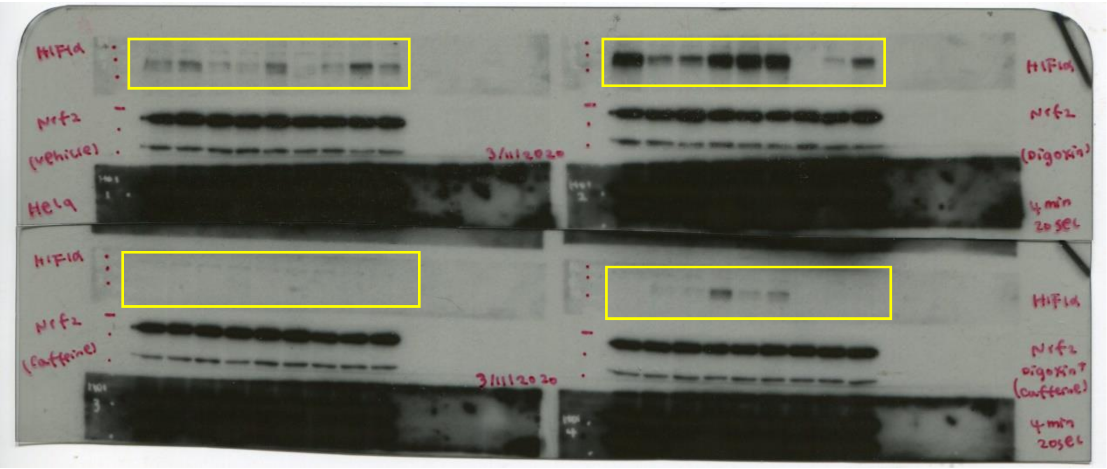
HIF1α
Nrf2
(vehicle)
Hela
3/11/2020
HIF1α
Nrf2
(Digoxin)
4min 20sec
HIF1α
Nrf2
(caffeine)
3/11/2020
HIF1α
Nrf2
Digoxin+ (caffeine)
4min 20sec

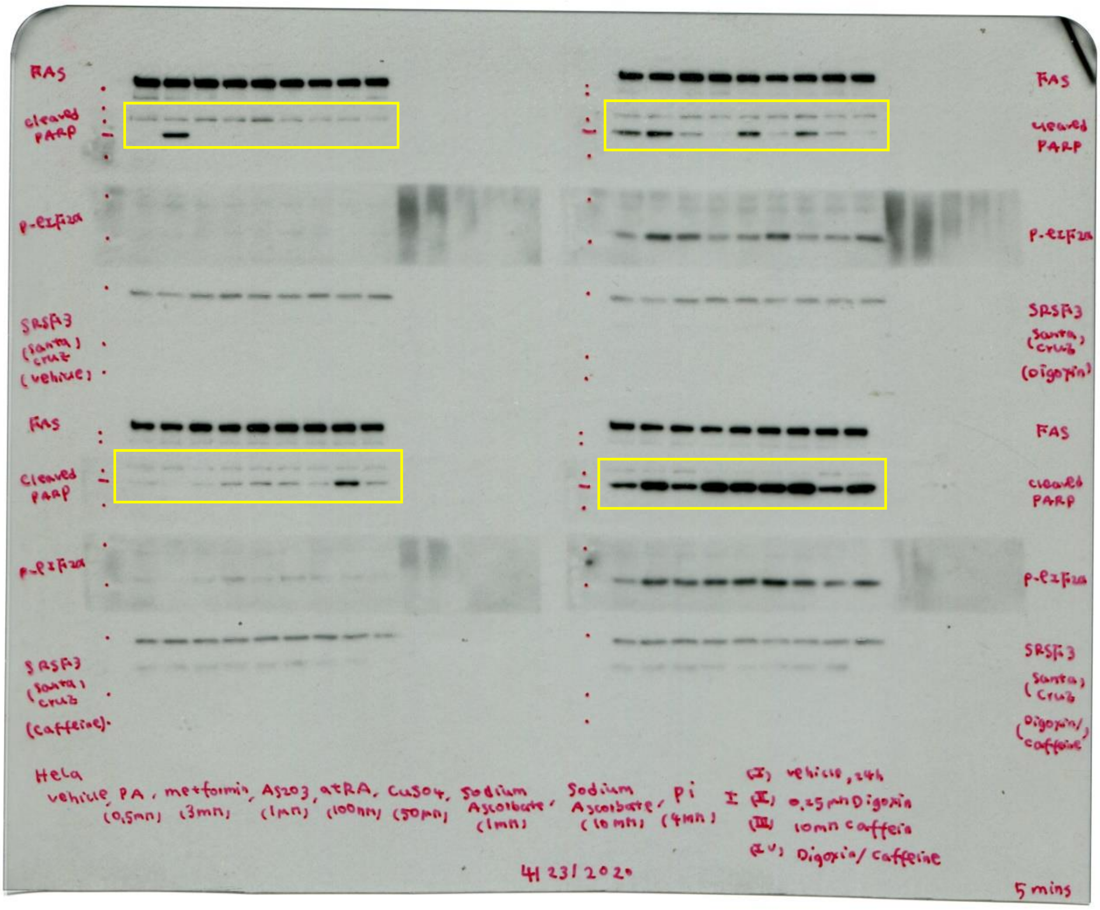
FAS
cleaved PARP
p-eIF2α
SRSF3 (santa cruz) (vehicle)
FAS
cleaved PARP
p-eIF2α
SRSF3 (santa cruz) (digoxin)
FAS
cleaved PARP
p-eIF2α
SRSF3 (santa cruz) (caffeine)
FAS
cleaved PARP
p-eIF2α
SRSF3 (santa cruz) (Digoxin/caffeine)
Hela
vehicle, PA (0.5mM), metformin (3mM), As2O3 (1mM), atRA (100nM), CuSO4 (50μM), Sodium Ascorbate (1mM), Sodium Ascorbate (10mM), Pi (4mM)
I
(I) vehicle, 24h
(II) 0.25μM Digoxin
(III) 10mM caffeine
(IV) Digoxin/caffeine
4/23/2020
5 mins

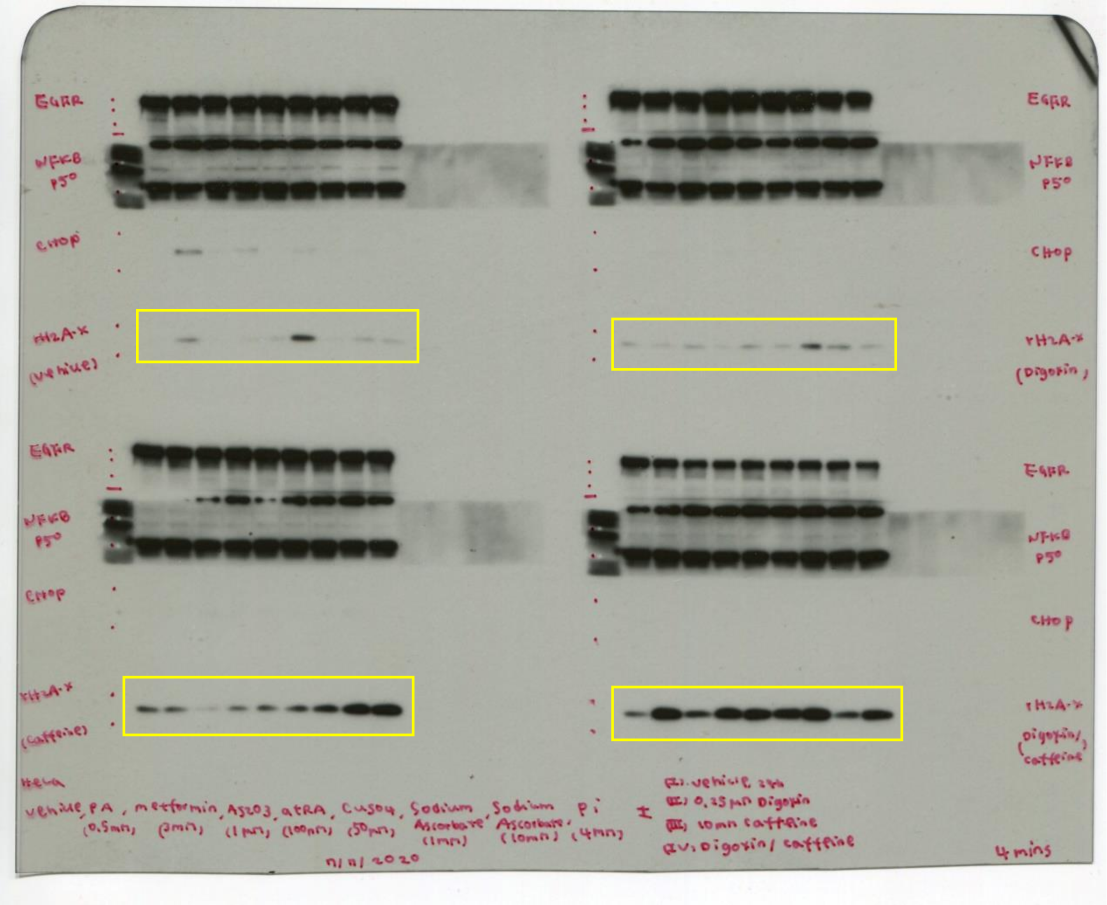
EGFR
NFKB p50
CHOP
γH2A-X (vehicle)
γH2A-X (Digoxin)
EGFR
NFKB p50
CHOP
γH2A-X (caffeine)
γH2A-X (digoxin/caffeine)
Hela
vehicle, PA (0.5mM), metformin (2mM), As2O3 (1μM), atRA (100nM), CuSO4 (50μM), Sodium Ascorbate (1mM), Sodium Ascorbate (10mM), Pi (4mM)
I: vehicle 24h
II: 0.25μM Digoxin
III: 10mM caffeine
IV: Digoxin/caffeine
11/8/2020
4 mins

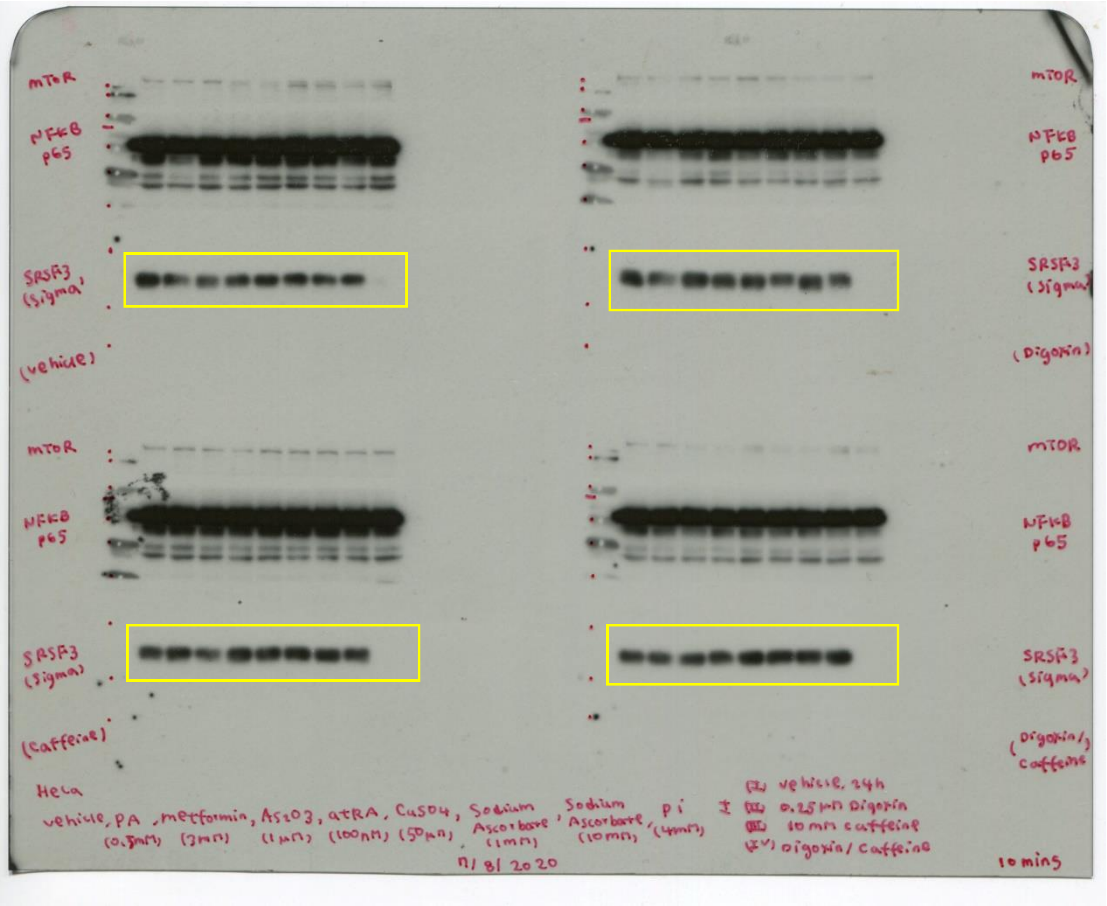
mTOR
NFKB p65
SRSF3 (Sigma)
(vehicle)
(Digoxin)
mTOR
NFKB p65
SRSF3 (Sigma)
(caffeine)
(Digoxin/caffeine)
Hela
vehicle, PA (0.5mM), metformin (2mM), As2O3 (1μM), atRA (100nM), CuSO4 (50μM), Sodium Ascorbate (1mM), Sodium Ascorbate (10mM), Pi (4mM)
I: vehicle 24h
II: 0.25μM Digoxin
III: 10mM caffeine
IV: Digoxin/caffeine
11/8/2020
10 mins

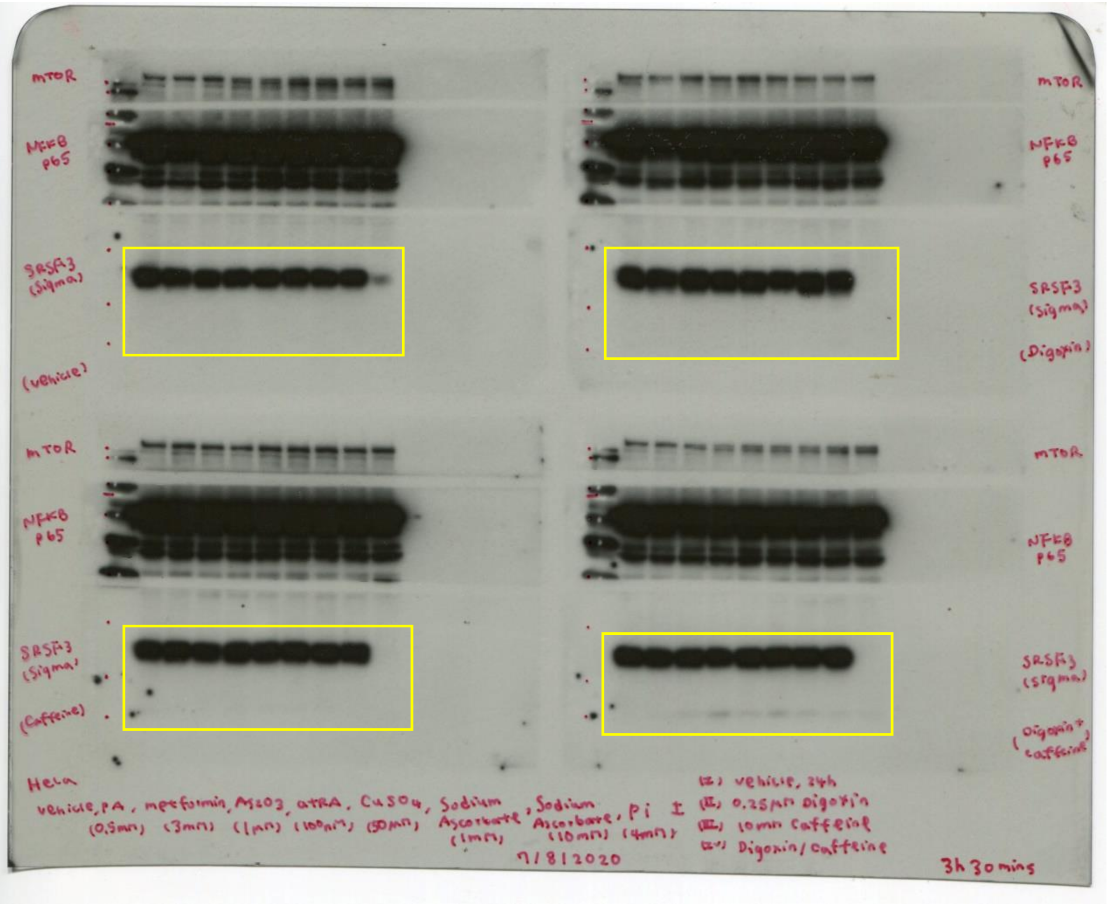
mTOR
NFKB p65
SRSF3 (Sigma)
(vehicle)
(Digoxin)
(Caffeine)
(Digoxin+caffeine)
Hela
vehicle, PA (0.5mM), metformin (3mM), As2O3 (1µM), atRA (100nM), CuSO4 (50µM), Sodium Ascorbate (1mM), Sodium Ascorbate (10mM), Pi (4mM)
I) vehicle, 24h
II) 0.25µM Digoxin
III) 10mM Caffeine
IV) Digoxin/caffeine
7/8/2020
3h 30mins

Figure 7A:

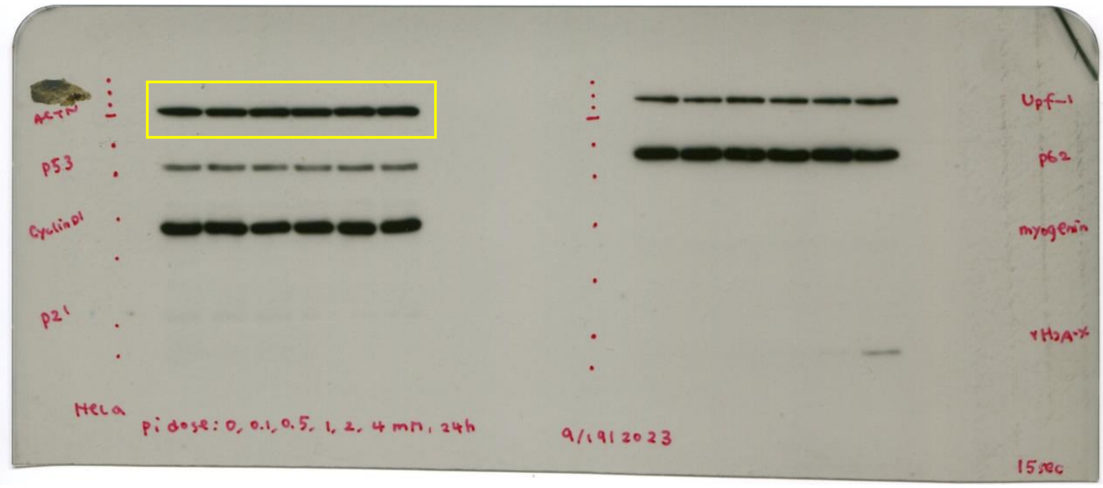
ACTN
p53
CyclinD1
p21
Upf-1
p62
myogenin
γH2A-X
Hela
Pi dose: 0, 0.1, 0.5, 1, 2, 4 mM, 24h
9/19/2023
15sec

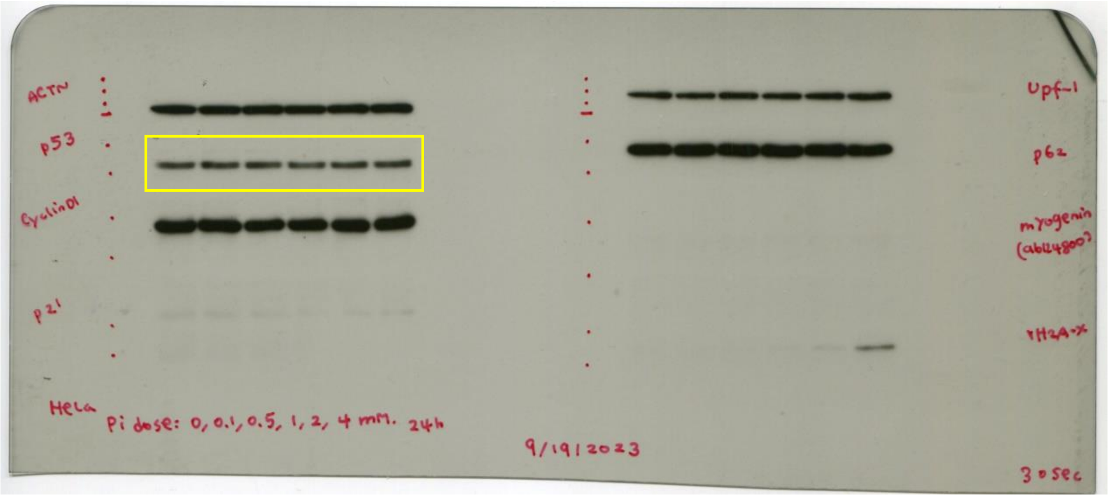
ACTN
p53
CyclinD1
p21
HeLa
Pi dose: 0, 0.1, 0.5, 1, 2, 4 mM. 24h
9/19/2023
Upf-1
p62
myogenin (ab124800)
γH2A.X
30sec

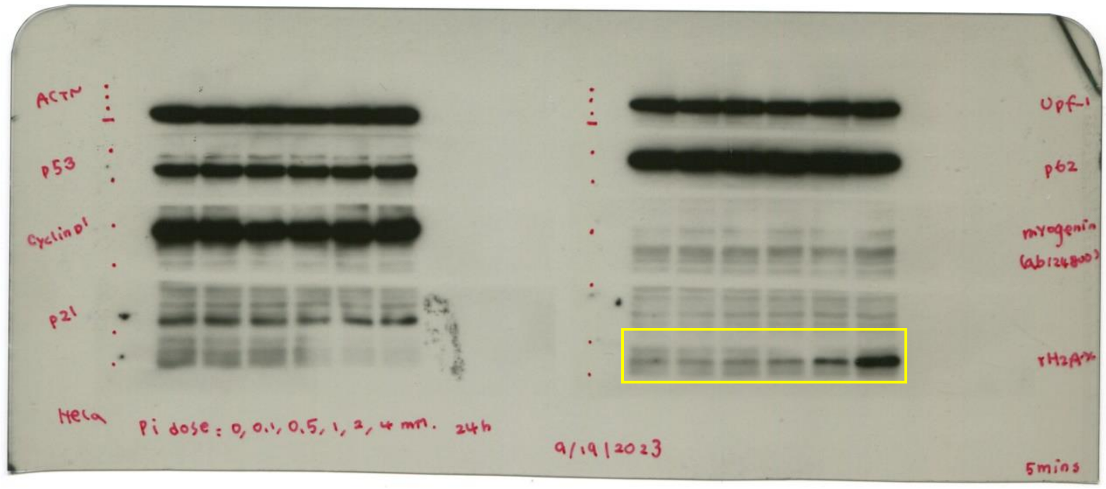
ACTN
p53
CyclinD1
p21
HeLa
Pi dose: 0, 0.1, 0.5, 1, 2, 4 mM. 24h
9/19/2023
Upf-1
p62
myogenin (ab124800)
γH2A.X
5mins

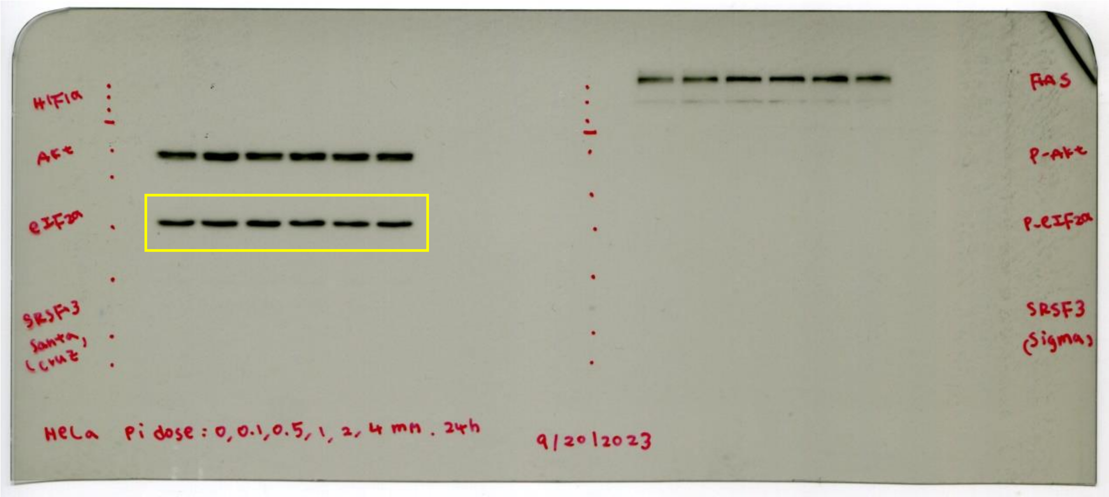
HIF1α
Akt
eIF2α
SRSF3 (Santa Cruz)
HeLa
Pi dose: 0, 0.1, 0.5, 1, 2, 4 mM. 24h
9/20/2023
FAS
P-Akt
P-eIF2α
SRSF3 (Sigma)

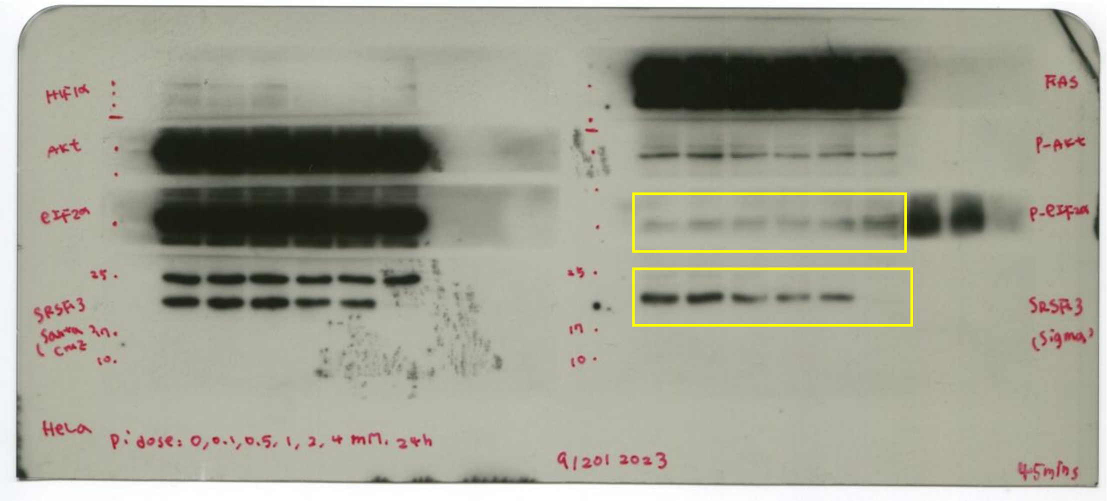
Akt
SRSF3
FAS
P-Akt
SRSF3
(Sigma)
HeLa
Pi dose: 0, 0.1, 0.5, 1, 2, 4 mM. 24h

Figure 7B:

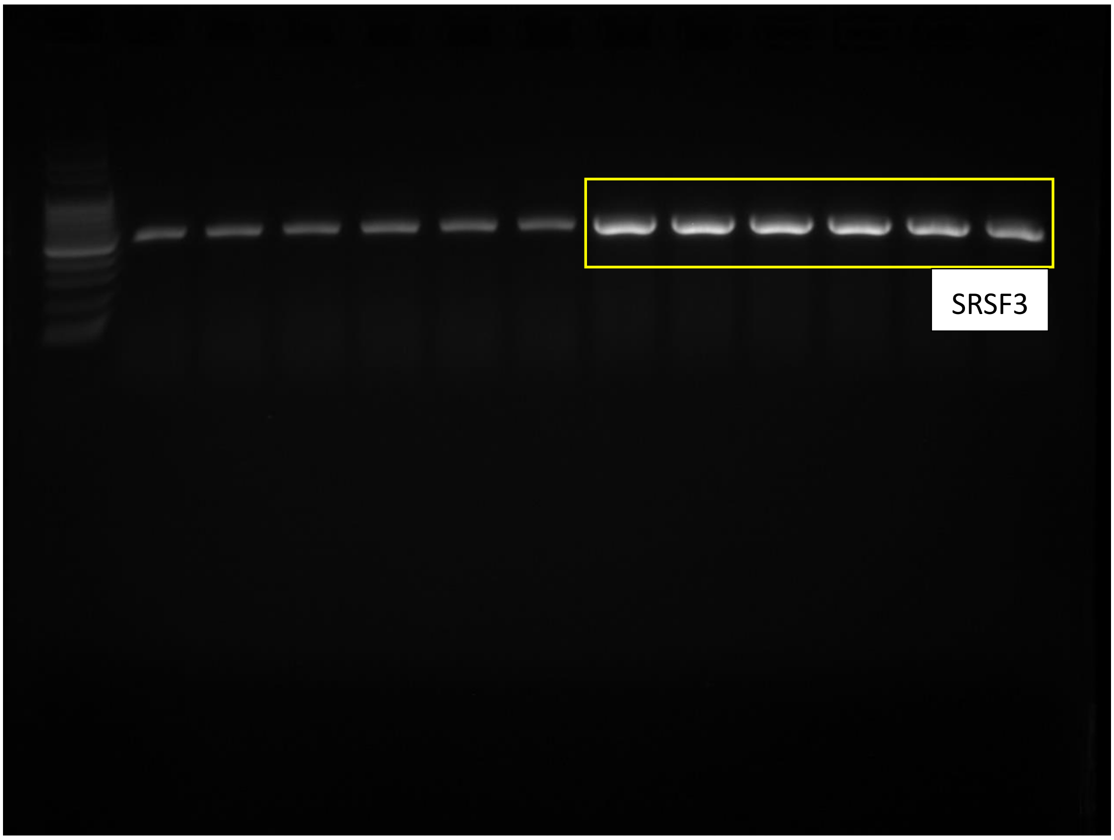
SRSF3

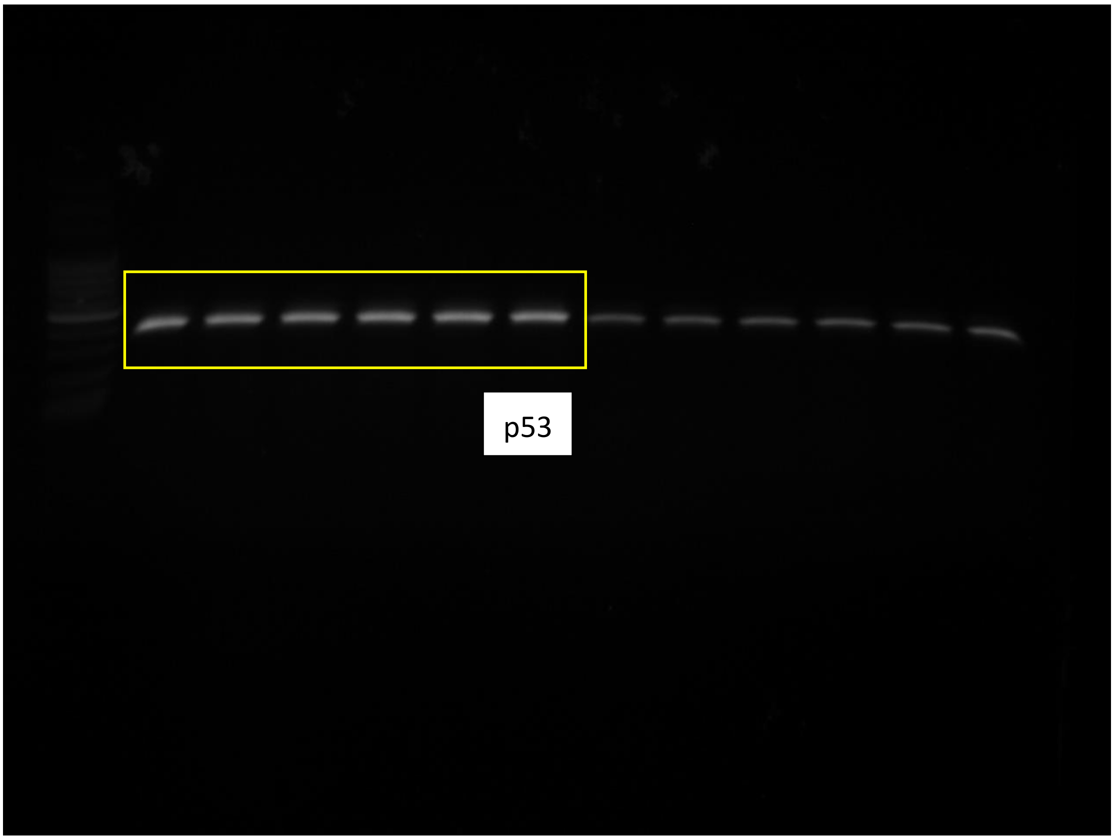
p53

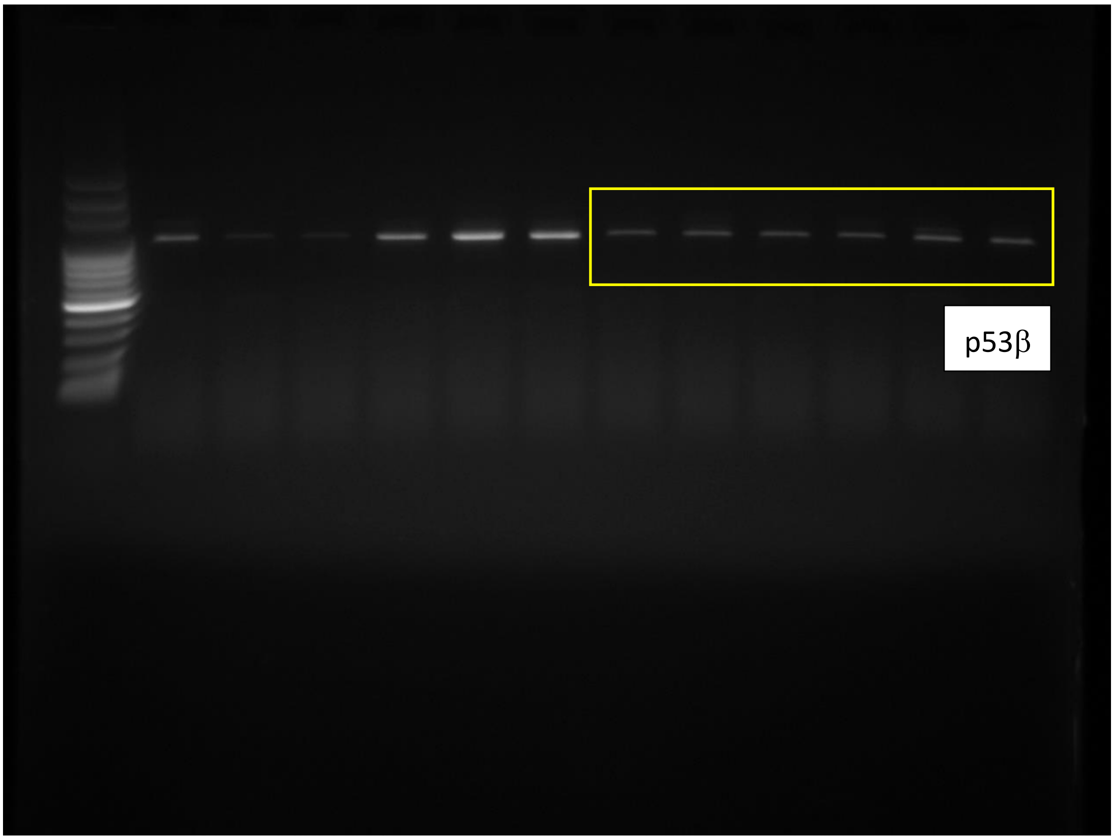
p53β

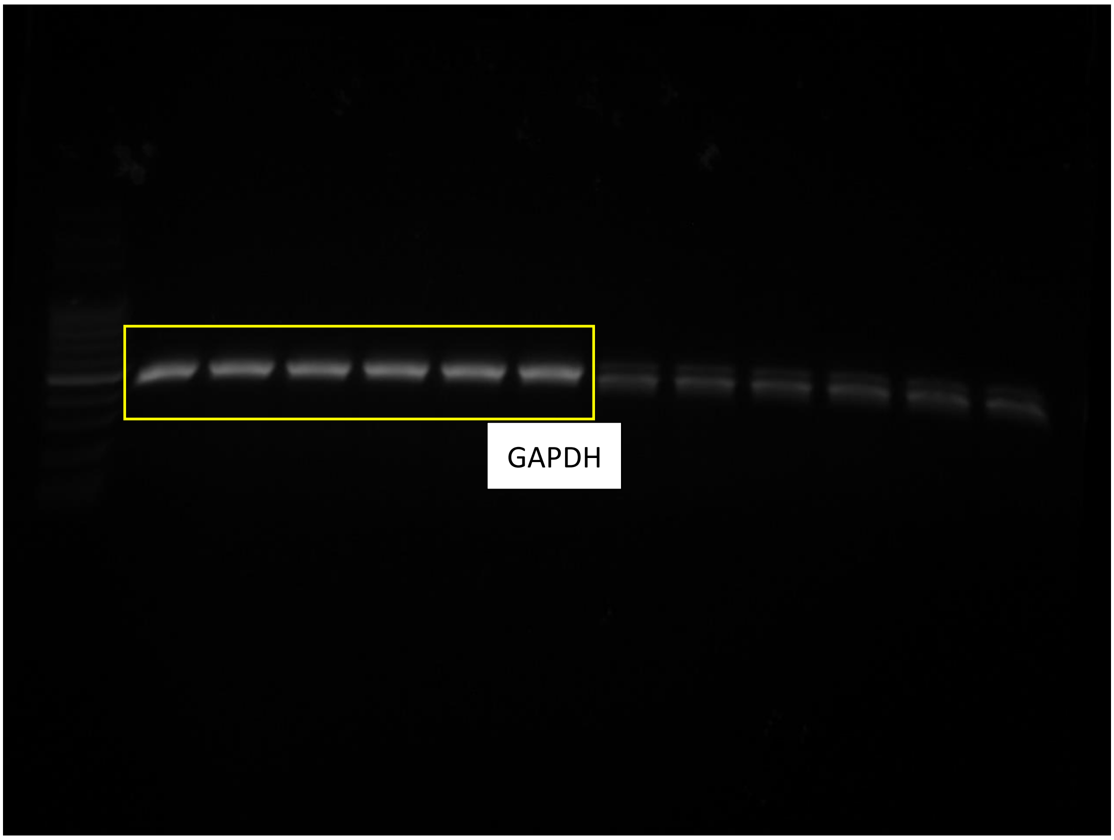

Figure 7C:

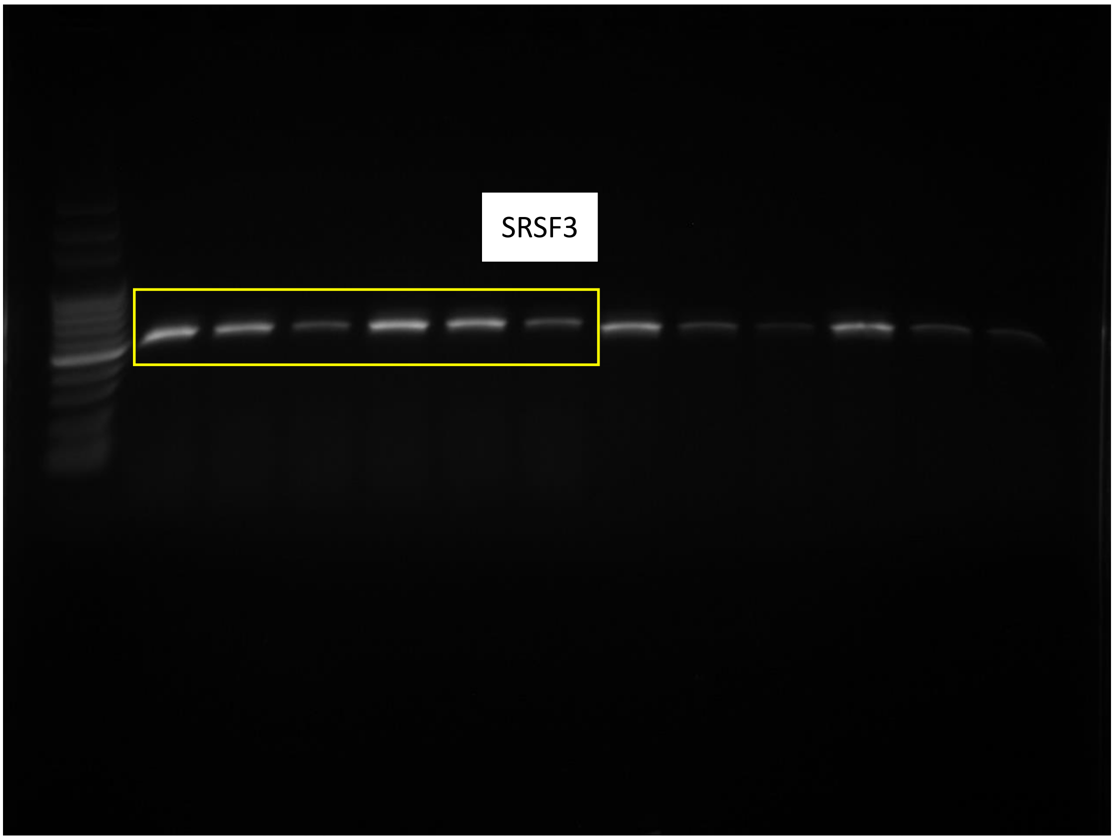

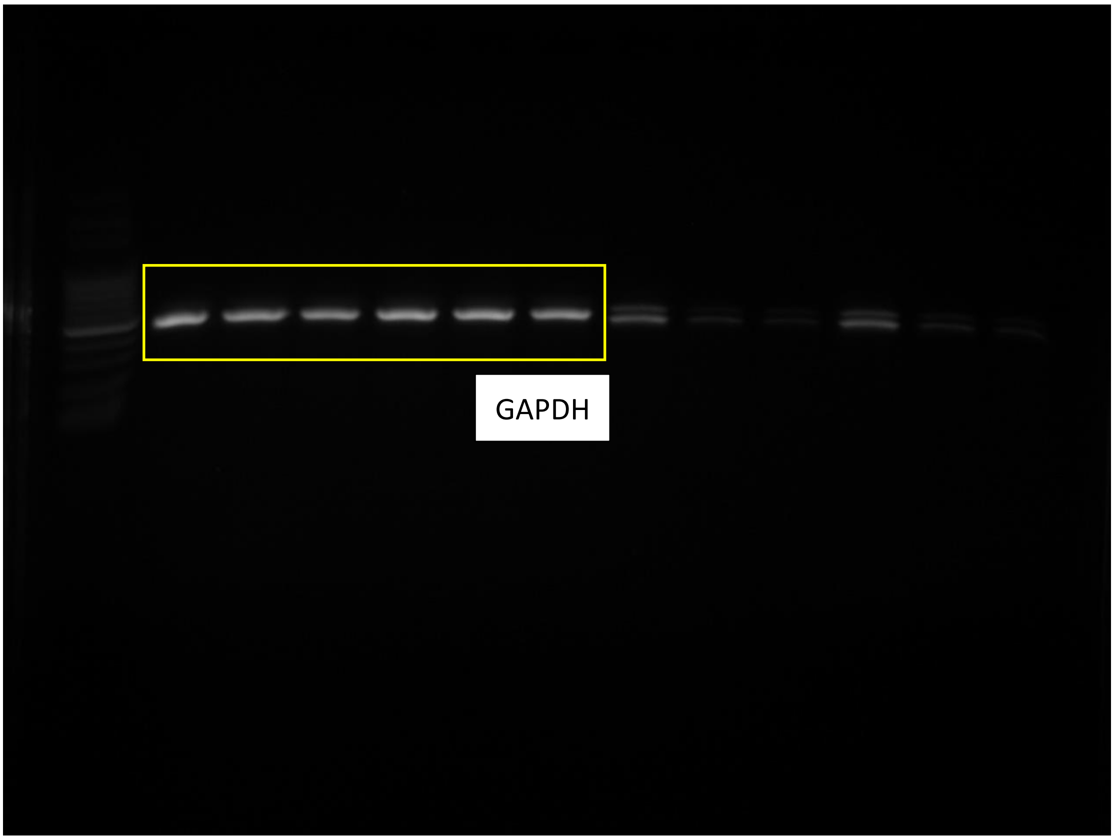
GAPDH

Figure 7D:

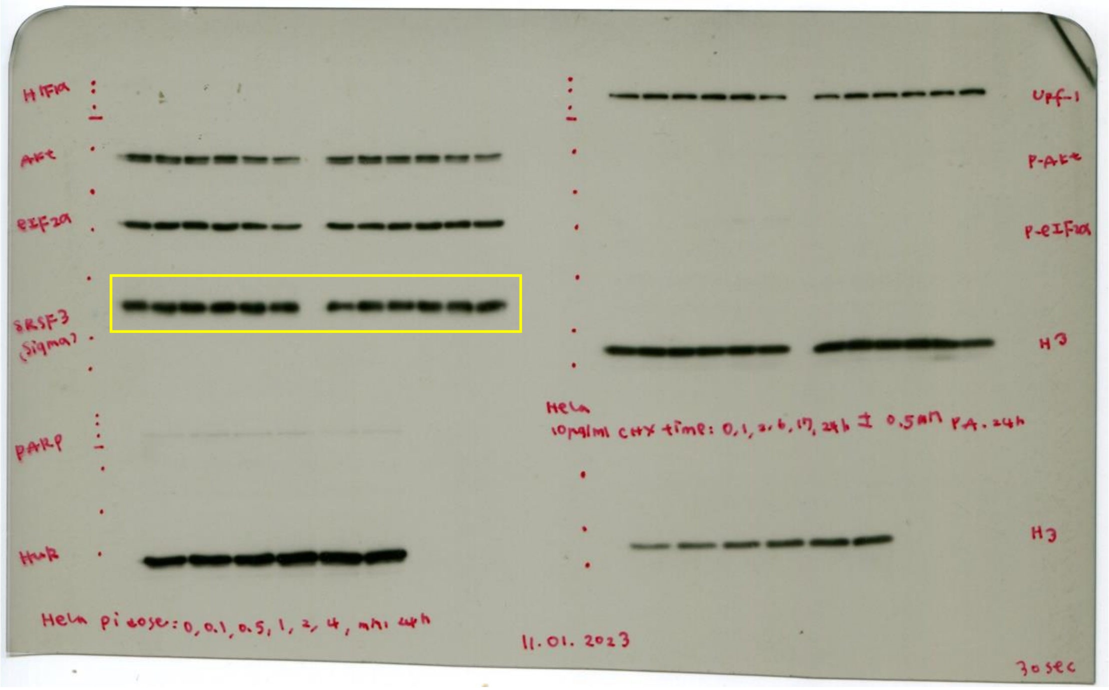
Akt
SRSF3
(Sigma)
HuR
Upf-1
P-Akt
H3
Hela
H3
11.01.2023
30sec

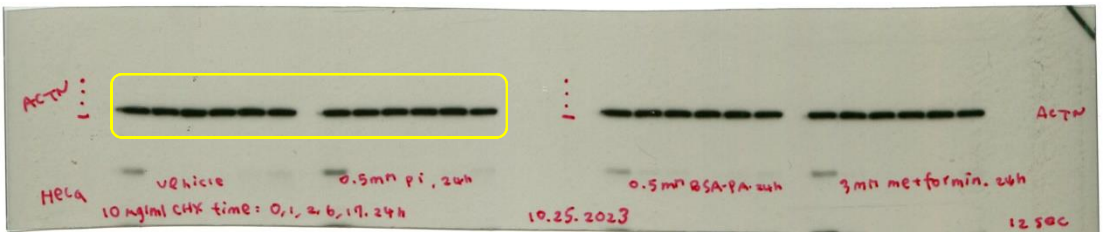
ACTN
HeLa
vehicle
0.5mM pi, 24h
10 ng/ml CHX time: 0, 1, 2, 6, 17, 24h
0.5mM BSA-PA. 24h
3mM metformin. 24h
10.25.2023
12 sec

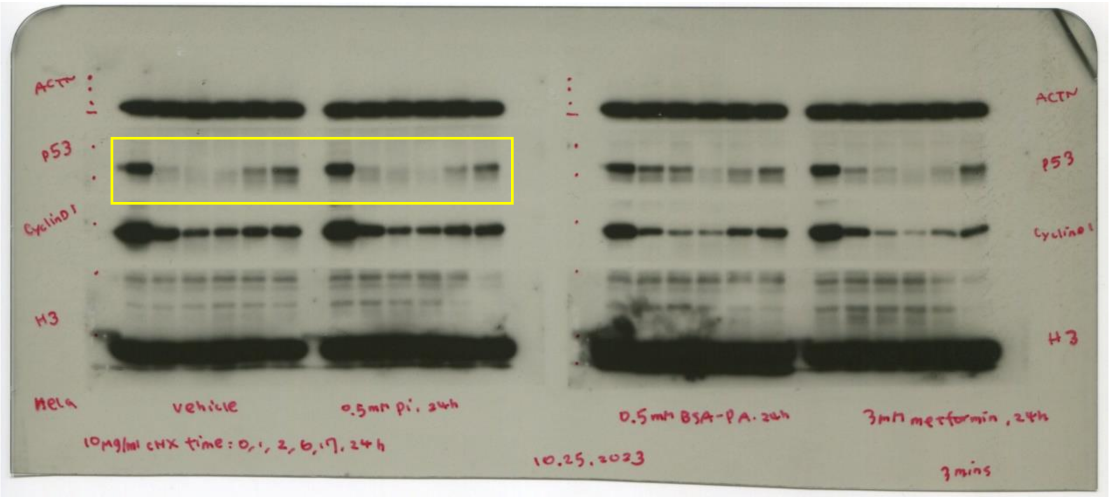
ACTN
p53
CyclinD1
H3
HeLa
vehicle
0.5mM pi. 24h
10mg/ml CHX time: 0, 1, 2, 6, 17, 24h
0.5mM BSA-PA. 24h
3mM metformin, 24h
10.25.2023
3 mins

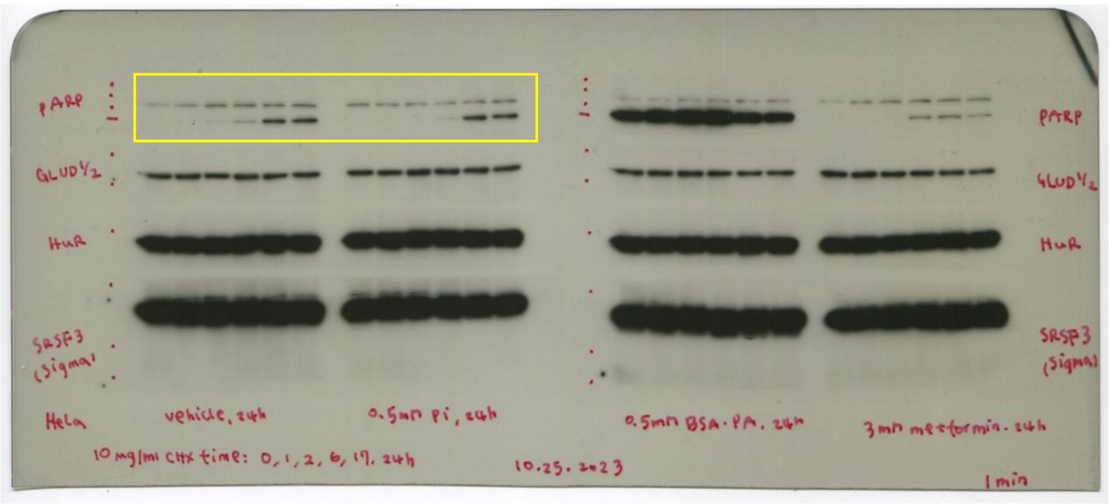
PARP
GLUD1/2
HuR
SRSF3 (sigma)
HeLa
vehicle, 24h
0.5mM pi, 24h
10 mg/ml CHX time: 0, 1, 2, 6, 17, 24h
0.5mM BSA-PA. 24h
3mM metformin. 24h
10.25.2023
1 min

Figure 7E:

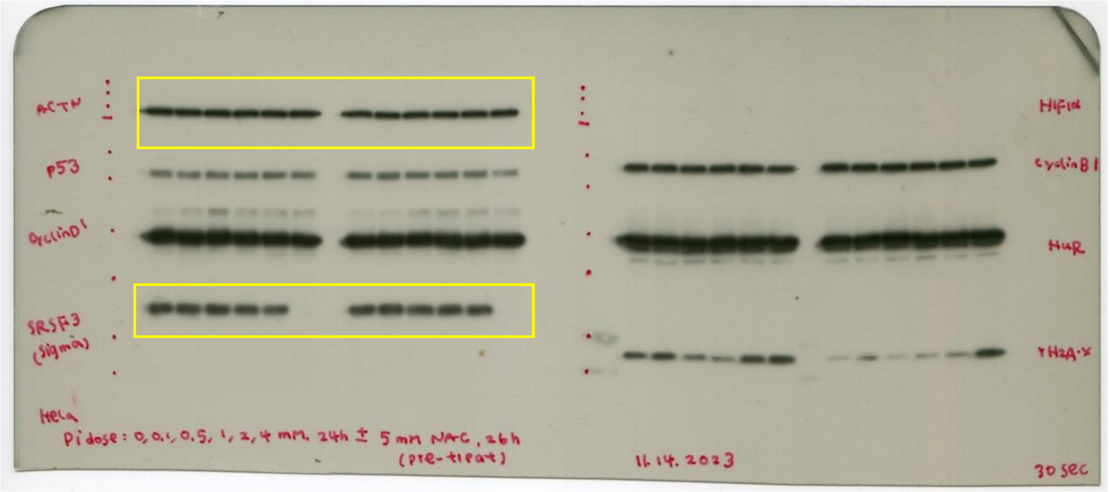
ACTN
p53
CyclinD1
SRSF3 (sigma)
HIF1α
cyclinB1
HuR
γH2A.X
HeLa
Pi dose: 0, 0.1, 0.5, 1, 2, 4 mM. 24h ± 5 mM NAC, 26h (pre-treat)
11.14.2023
30 sec

Figure 8A:

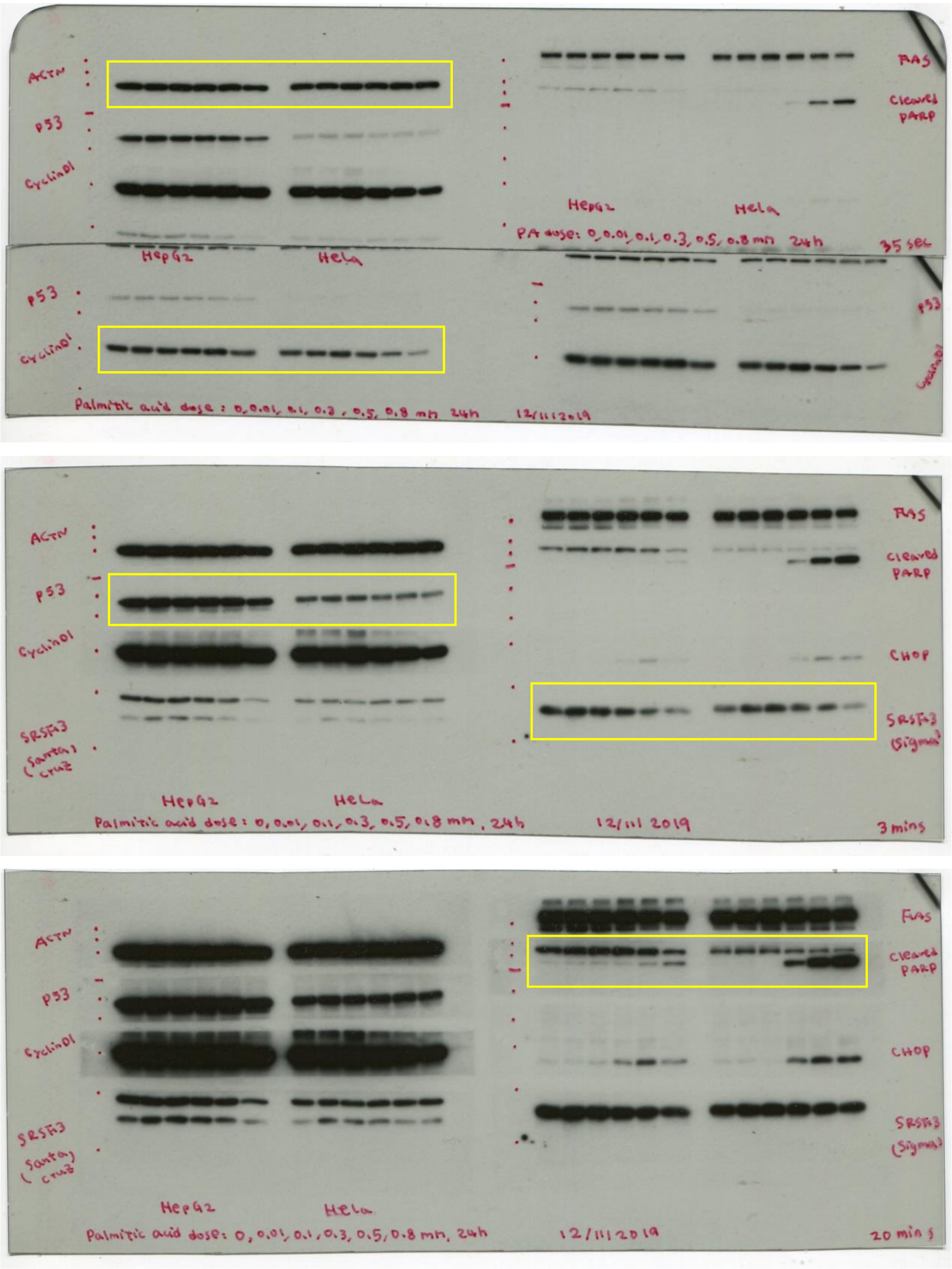
ACTN
p53
CyclinD1
HepG2
HeLa
FAS
Cleaved PARP
PA dose: 0, 0.01, 0.1, 0.3, 0.5, 0.8 mM 24h
35 sec
Palmitic acid dose: 0, 0.01, 0.1, 0.3, 0.5, 0.8 mM 24h
12/11/2019
CHOP
SRSF3
(Santa Cruz)
SRSF3
(Sigma)
3 mins
20 mins

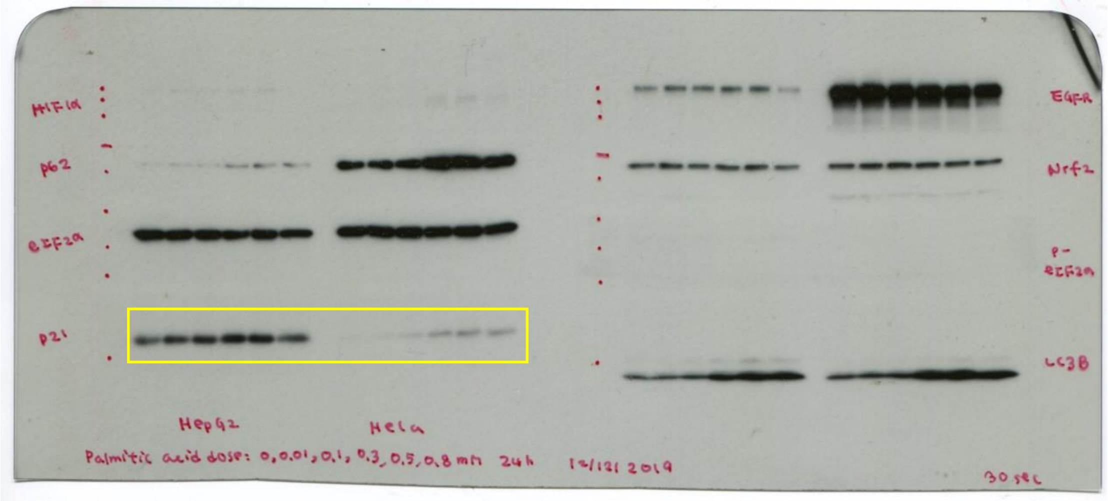
HIF1α
p62
eIF2α
p21
EGFR
Nrf2
p-eIF2α
LC3B
HepG2
HeLa
Palmitic acid dose: 0, 0.01, 0.1, 0.3, 0.5, 0.8 mM 24h
12/12/2019
30 sec

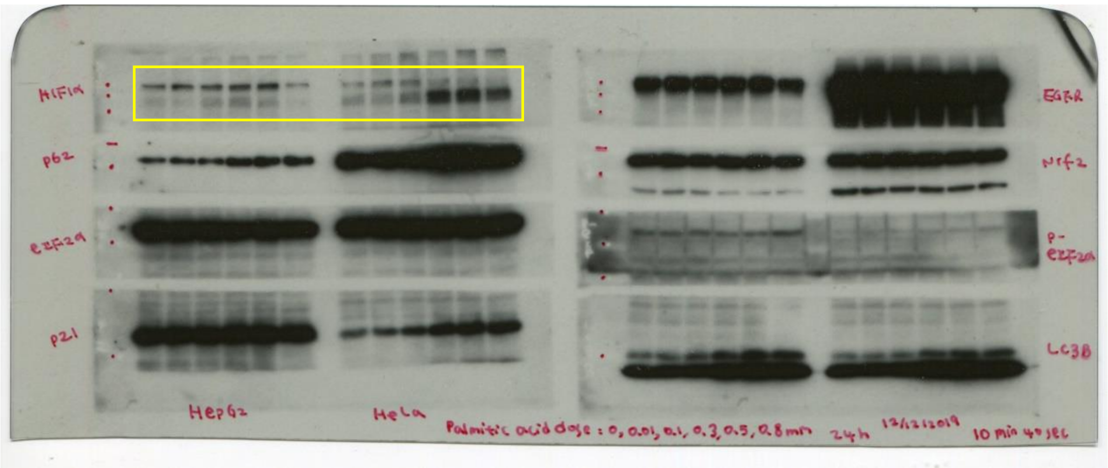
HIF1α
p62
eIF2α
p21
EGFR
Nrf2
p-eIF2α
LC3B
HepG2
HeLa
Palmitic acid dose: 0, 0.01, 0.1, 0.3, 0.5, 0.8 mM 24h
12/12/2019
10 min 40 sec

Figure 8B:

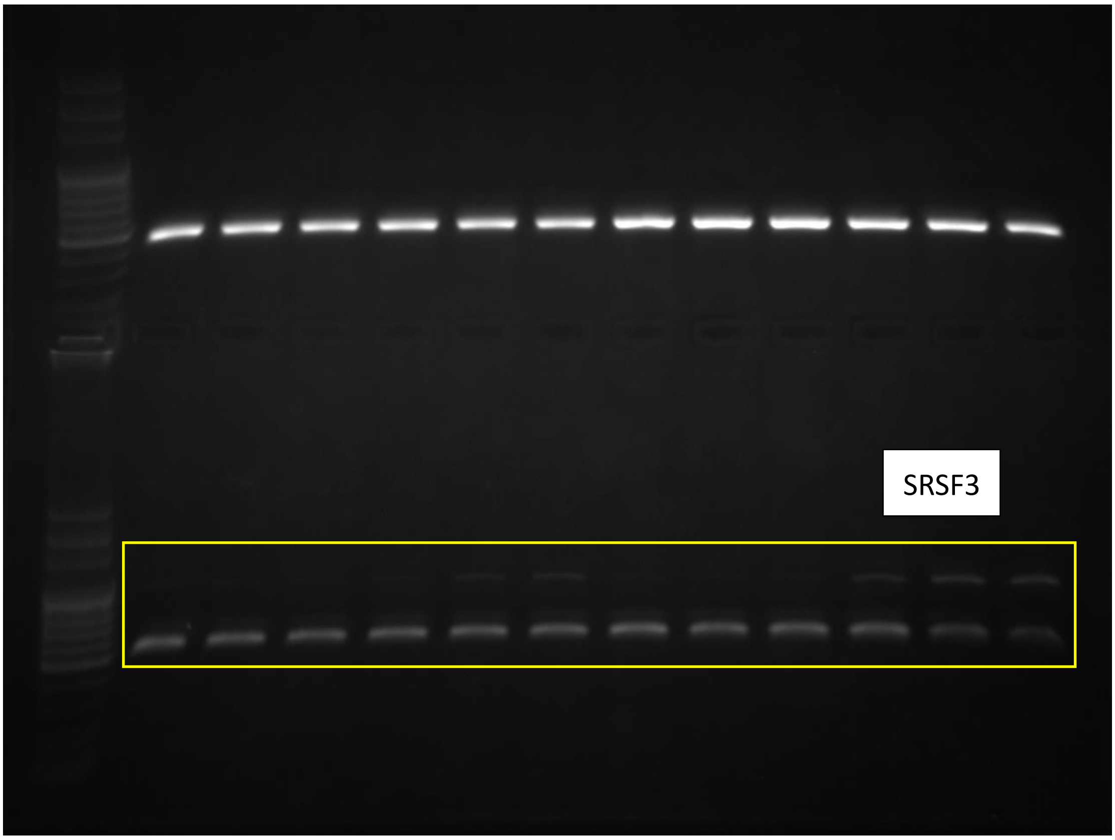
SRSF3

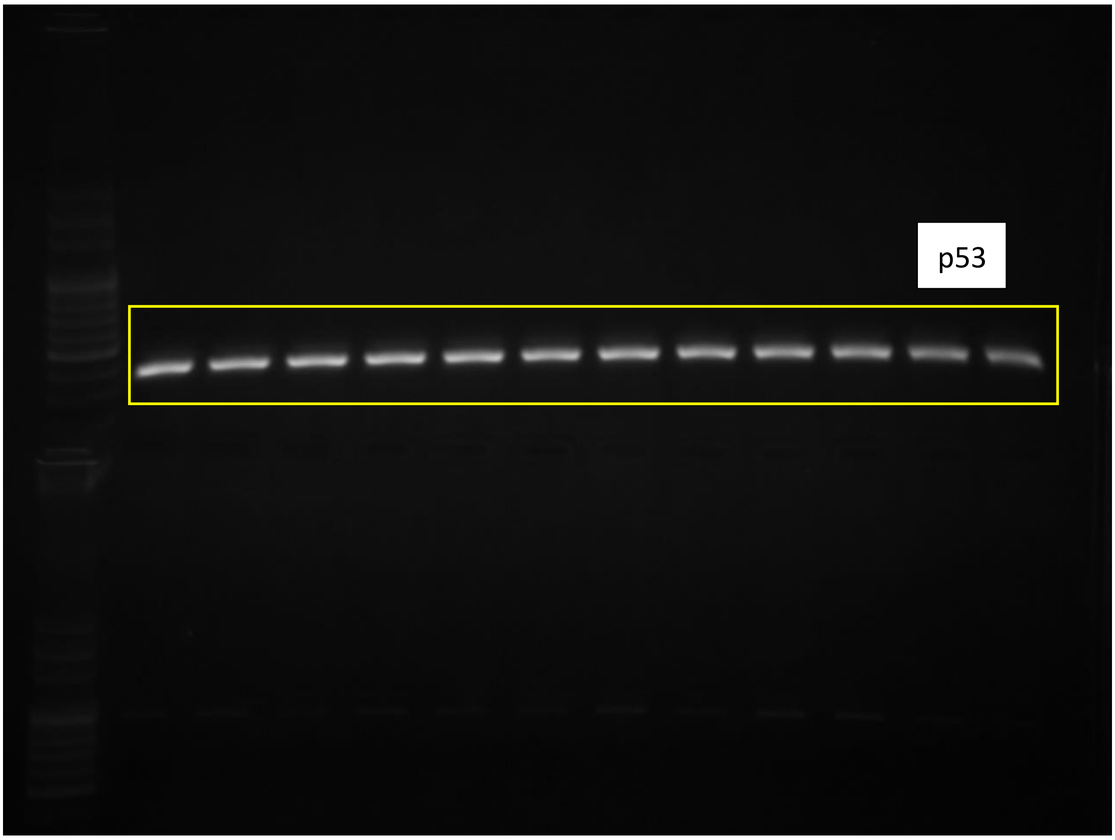
p53

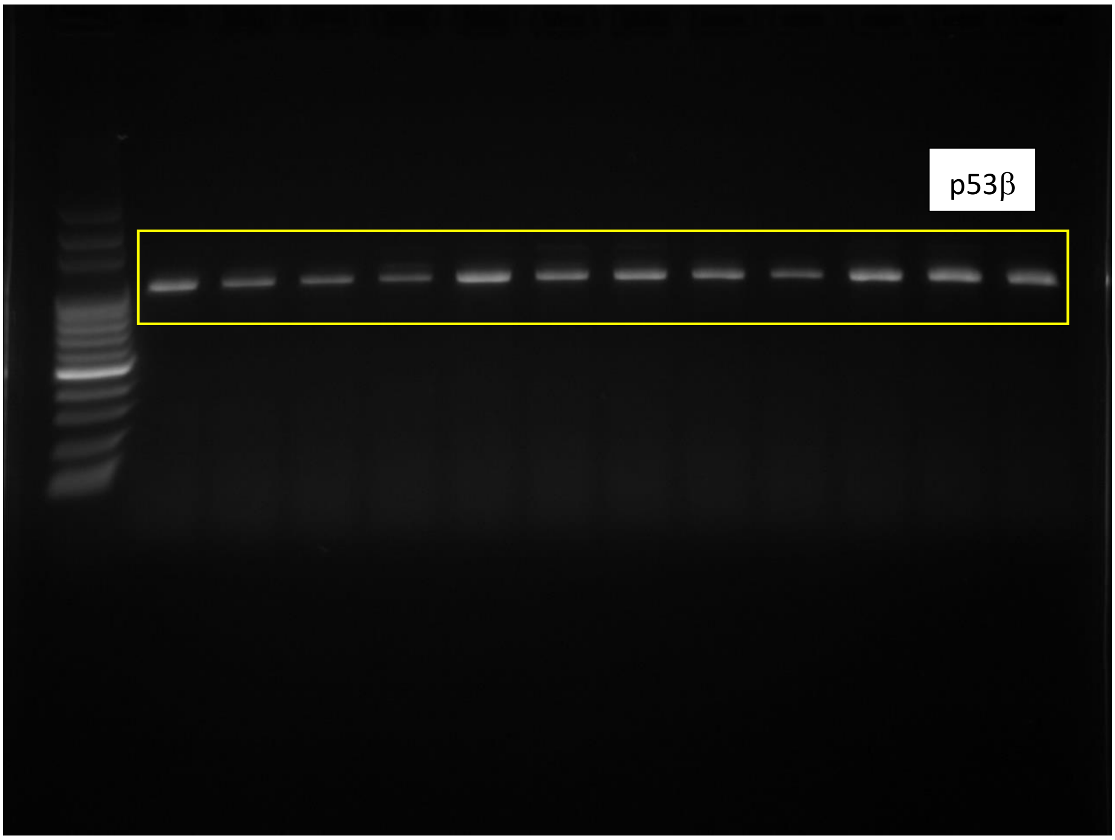
p53β

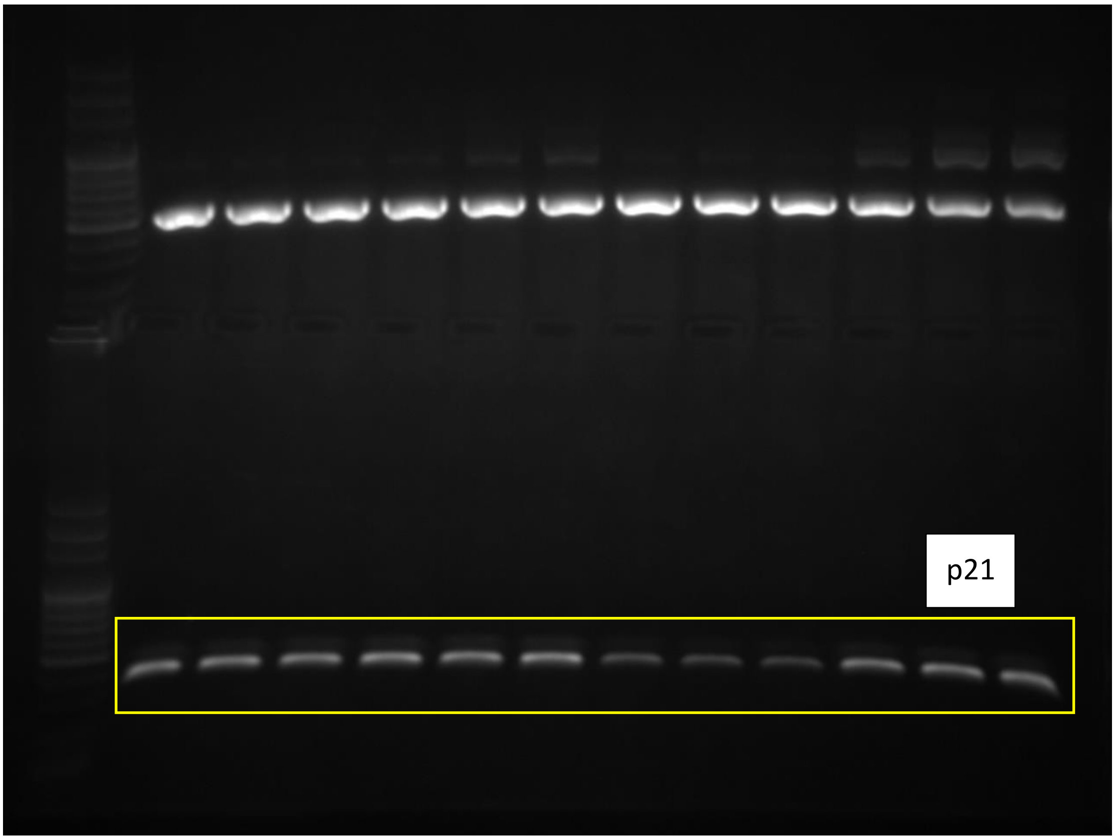
p21

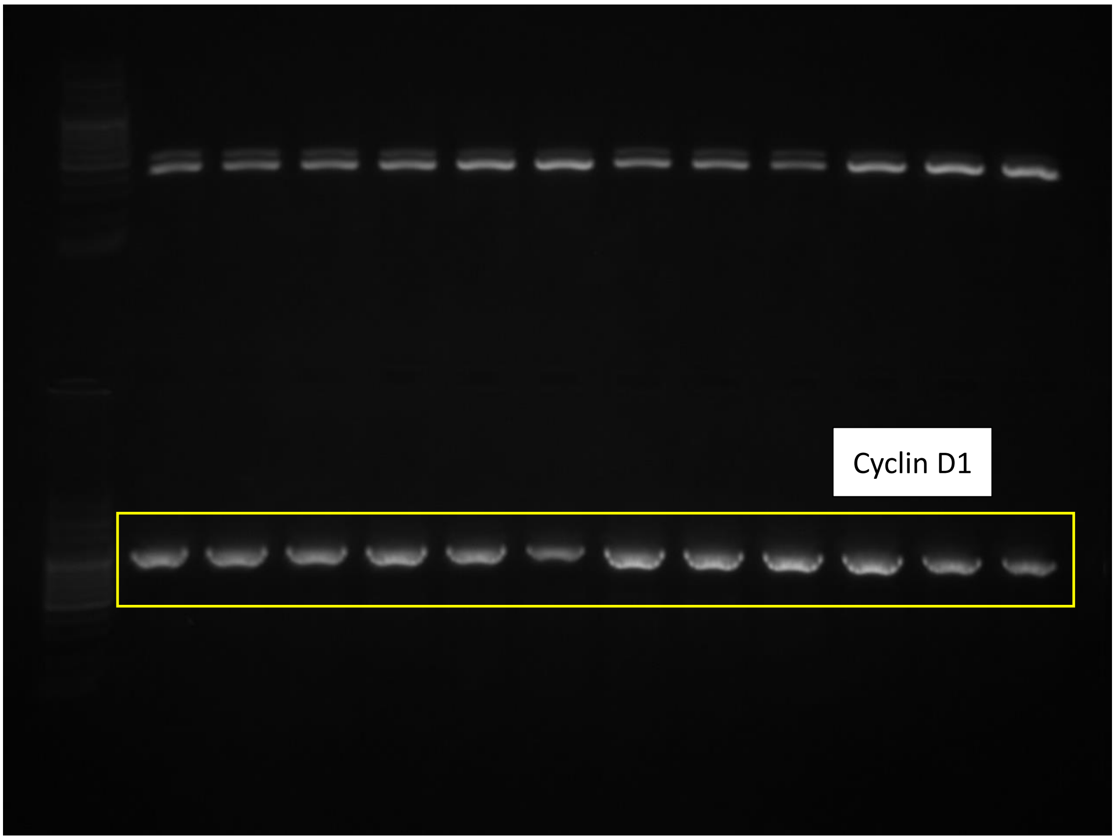
Cyclin D1

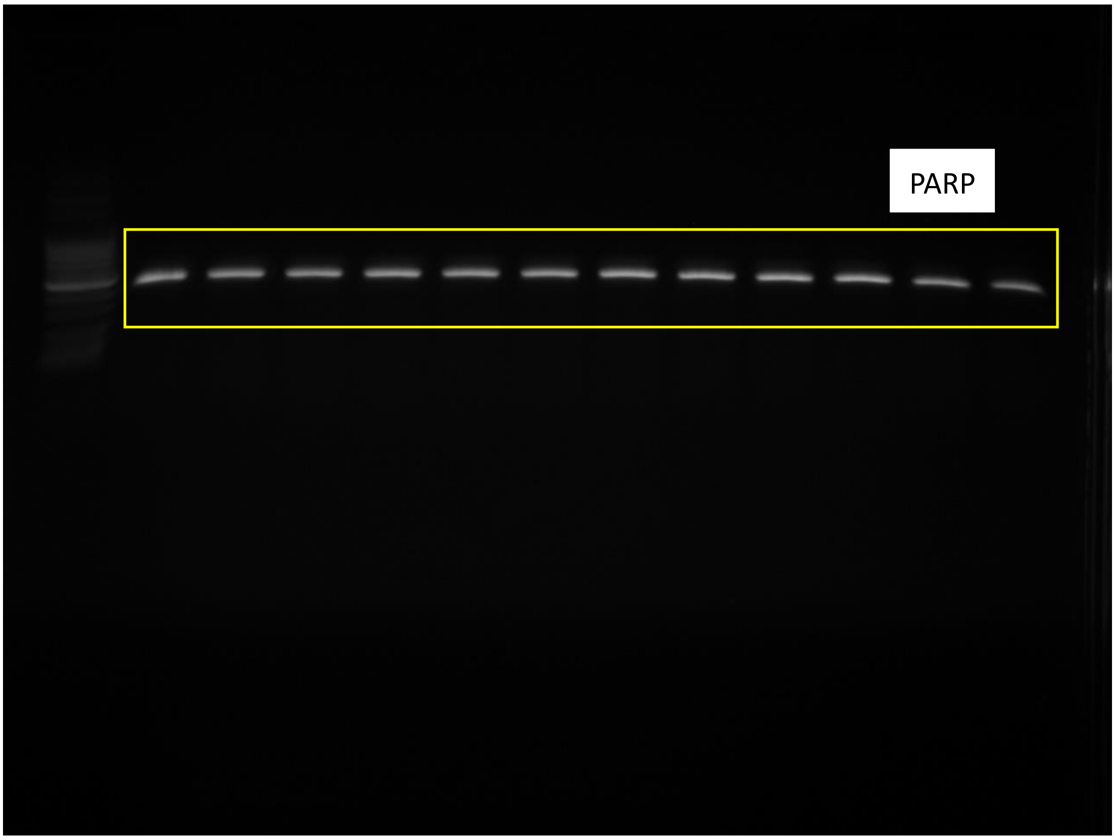
PARP

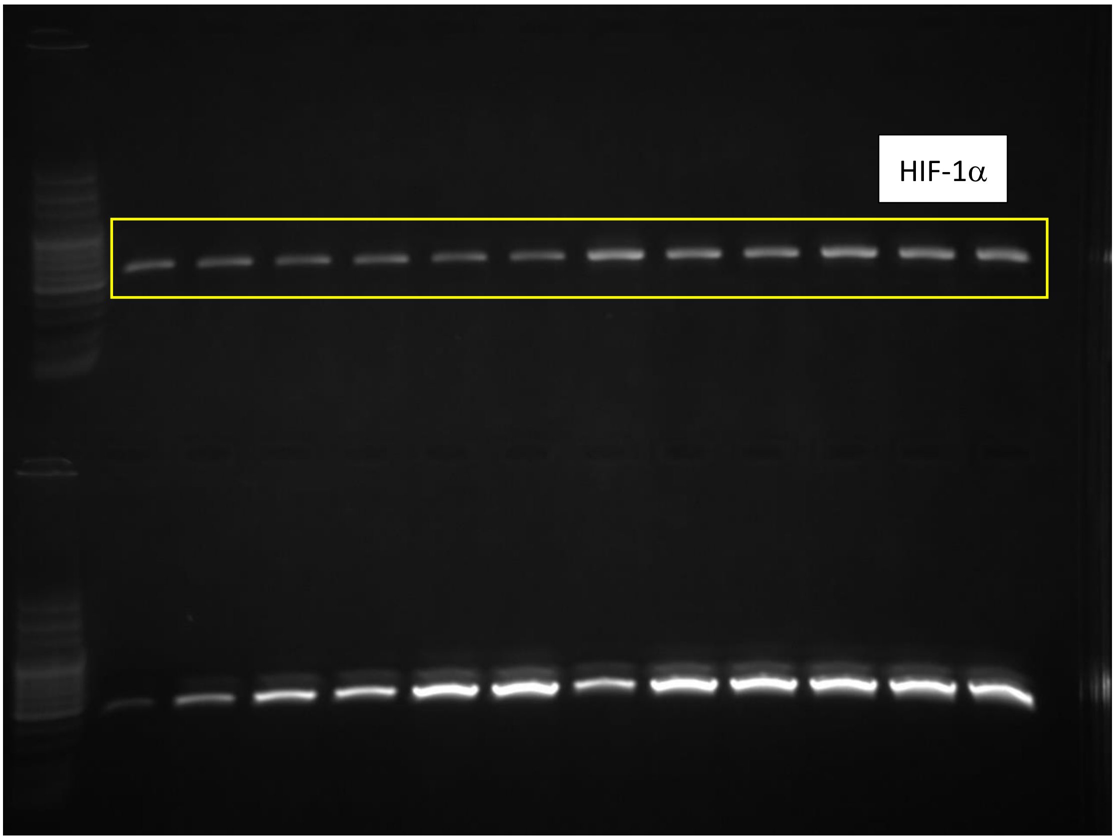
HIF-1α

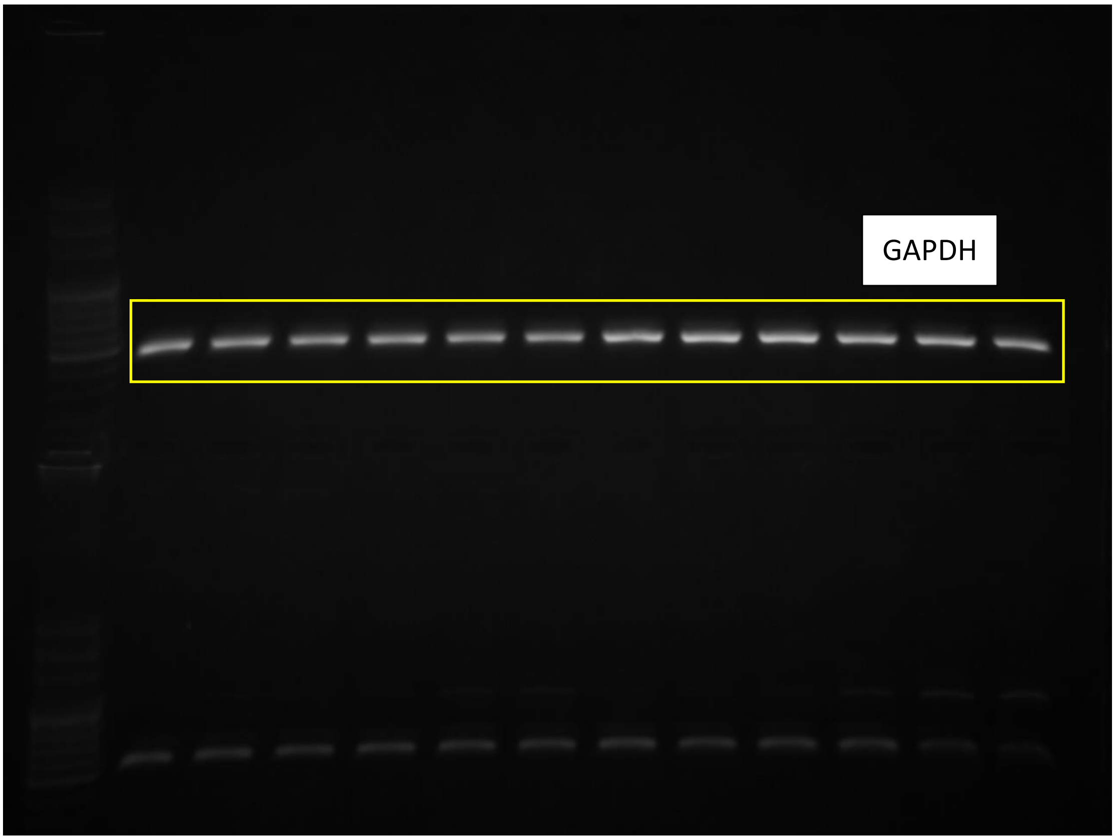
GAPDH
